# Supplementary material for: Genome-wide meta-analysis identifies 127 open-angle glaucoma loci with consistent effect across ancestries
Source: Nat Commun. 2021 Feb 24;12:1258. doi: 10.1038/s41467-020-20851-4 (PMC7904932; doi:10.1038/s41467-020-20851-4)
Supplement: Supplementary file 1 — Supplementary Information [file 41467_2020_20851_MOESM1_ESM.pdf]

Supplementary Information

Genome-wide meta-analysis identifies 127 open-angle glaucoma loci with consistent effect across ancestries

Supplementary Figures

Supplementary Figure 1. QQ and Manhattan plots. Q-Q plots (first plots on each panel) - the X-axis shows the expected distribution of -log10(P-values) under the null hypothesis of no association. The Y-axis shows the distribution of the observed -log10(P-values) in the meta-analyses. The red indicator lines show where X=Y. Manhattan plots (second plot on each panel) - each dot represents a SNP, the X-axis shows the chromosomes where each SNP is located, and the Y-axis shows -log10 P-value of the association of each SNP with POAG. The red horizontal line shows the genome-wide significant threshold (P-value=5e-8; -log10 P-value=7.30). The nearest gene to the most significant SNP in each locus has been labeled.

a) European meta-analysis (16,677 POAG cases vs 199,580 controls)

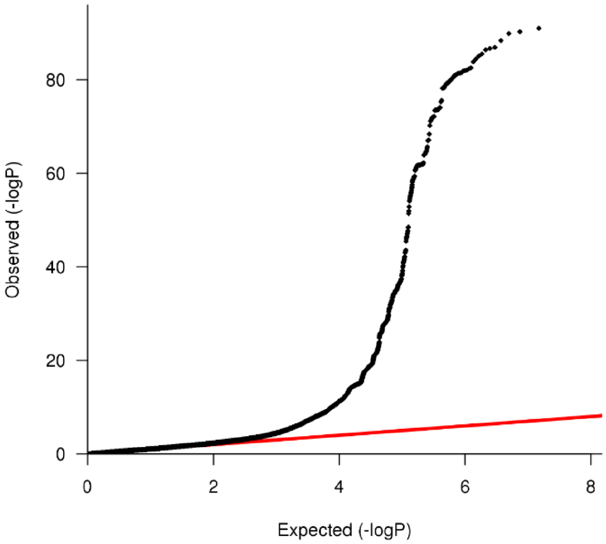

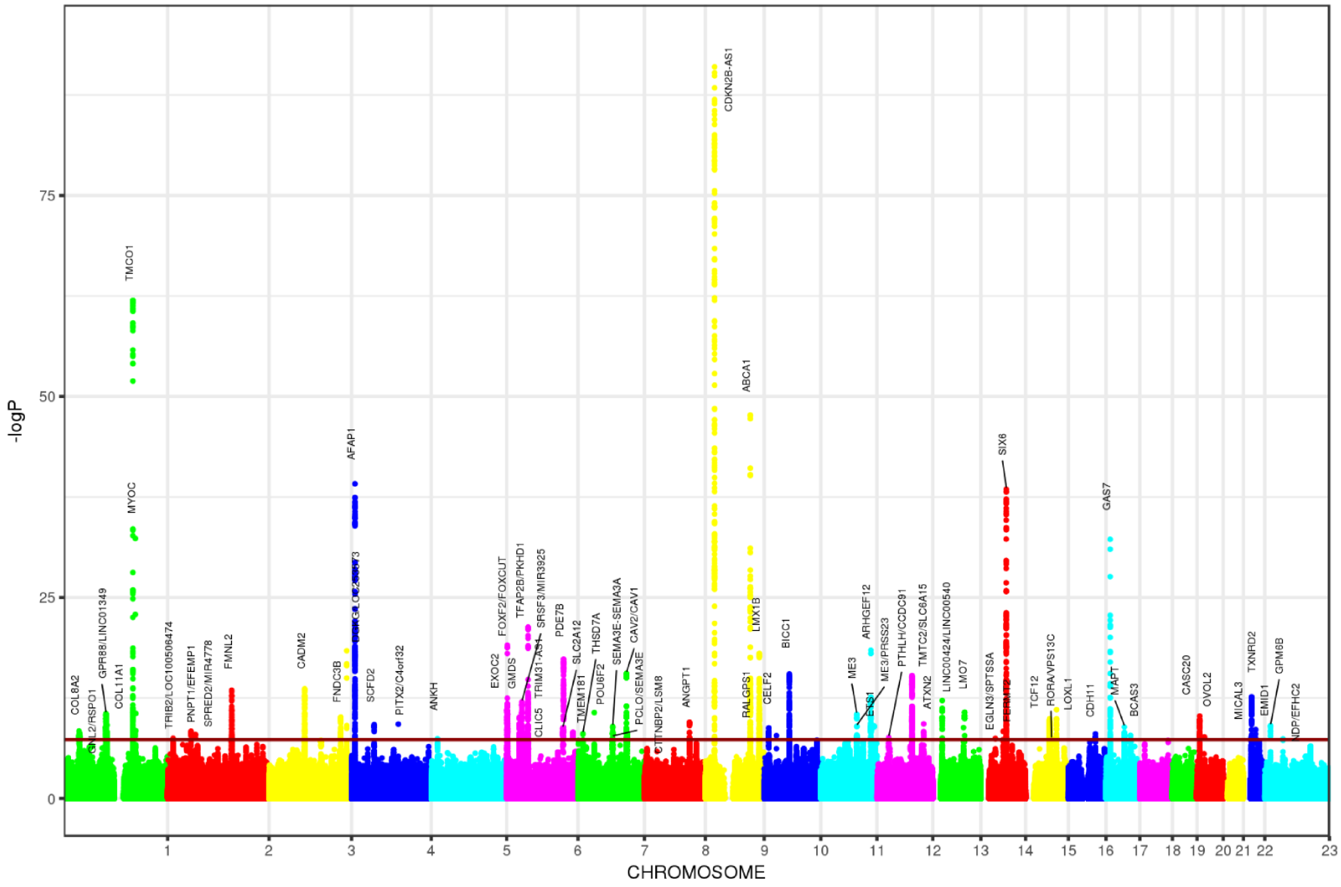

b) Cross-ancestry meta-analysis (34,179 cases vs 349,321 controls)

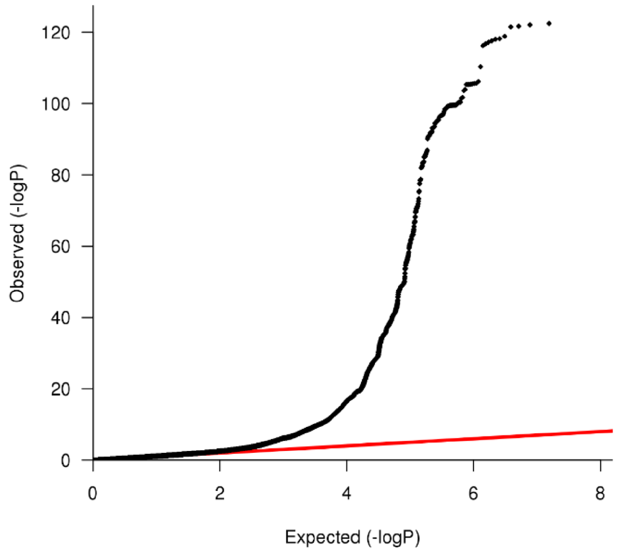

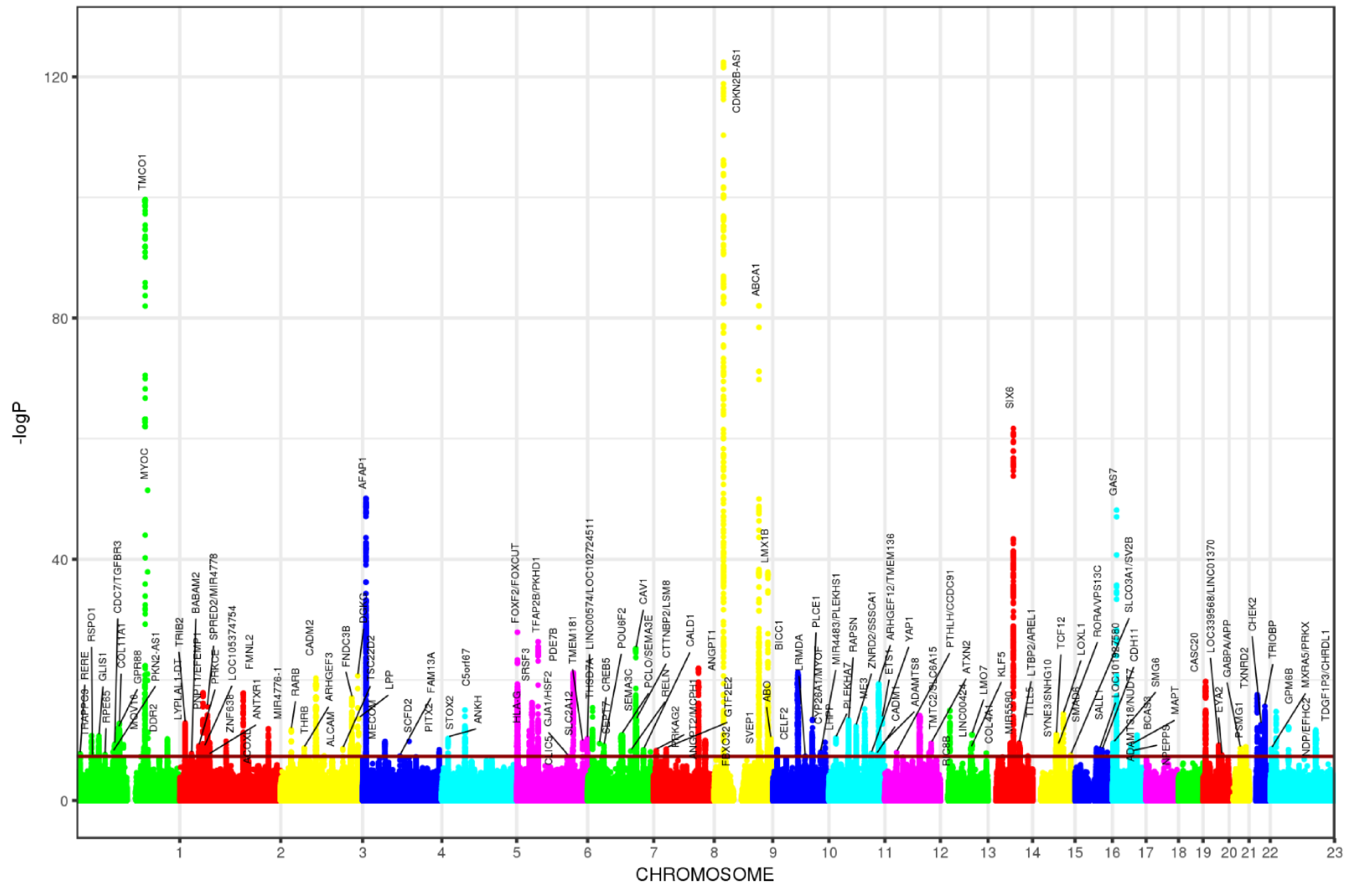

c) Male-stratified meta-analysis (10,775 cases vs 123,644 controls)

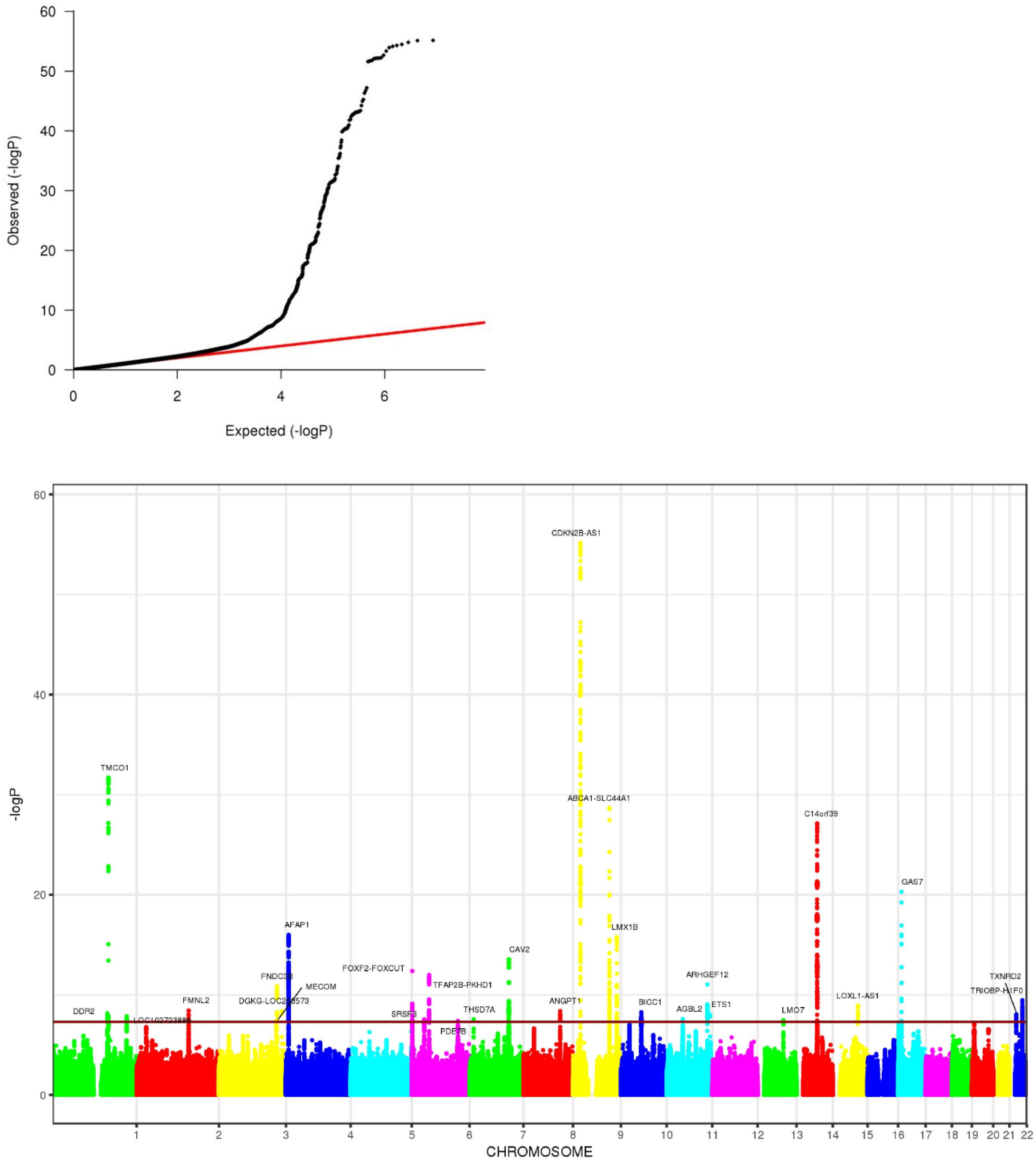

d) Female-stratified meta-analysis (10,977 cases vs 144,606 controls)

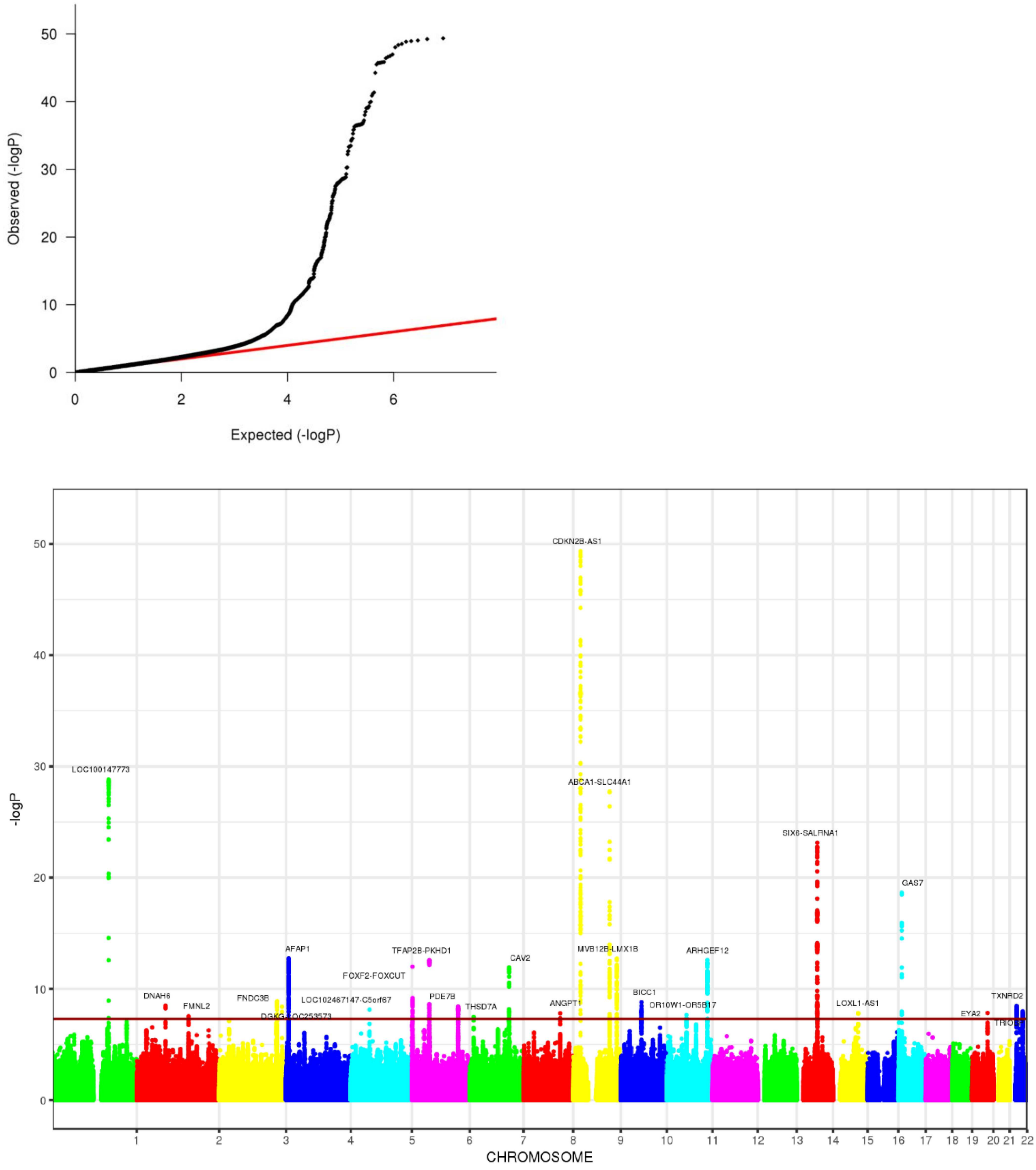

e) NTG-stratified meta-analysis (3,247 cases vs 47,997 controls)

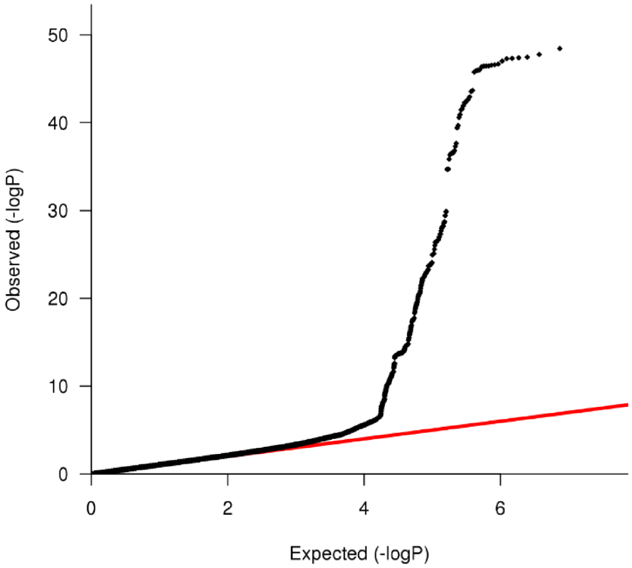

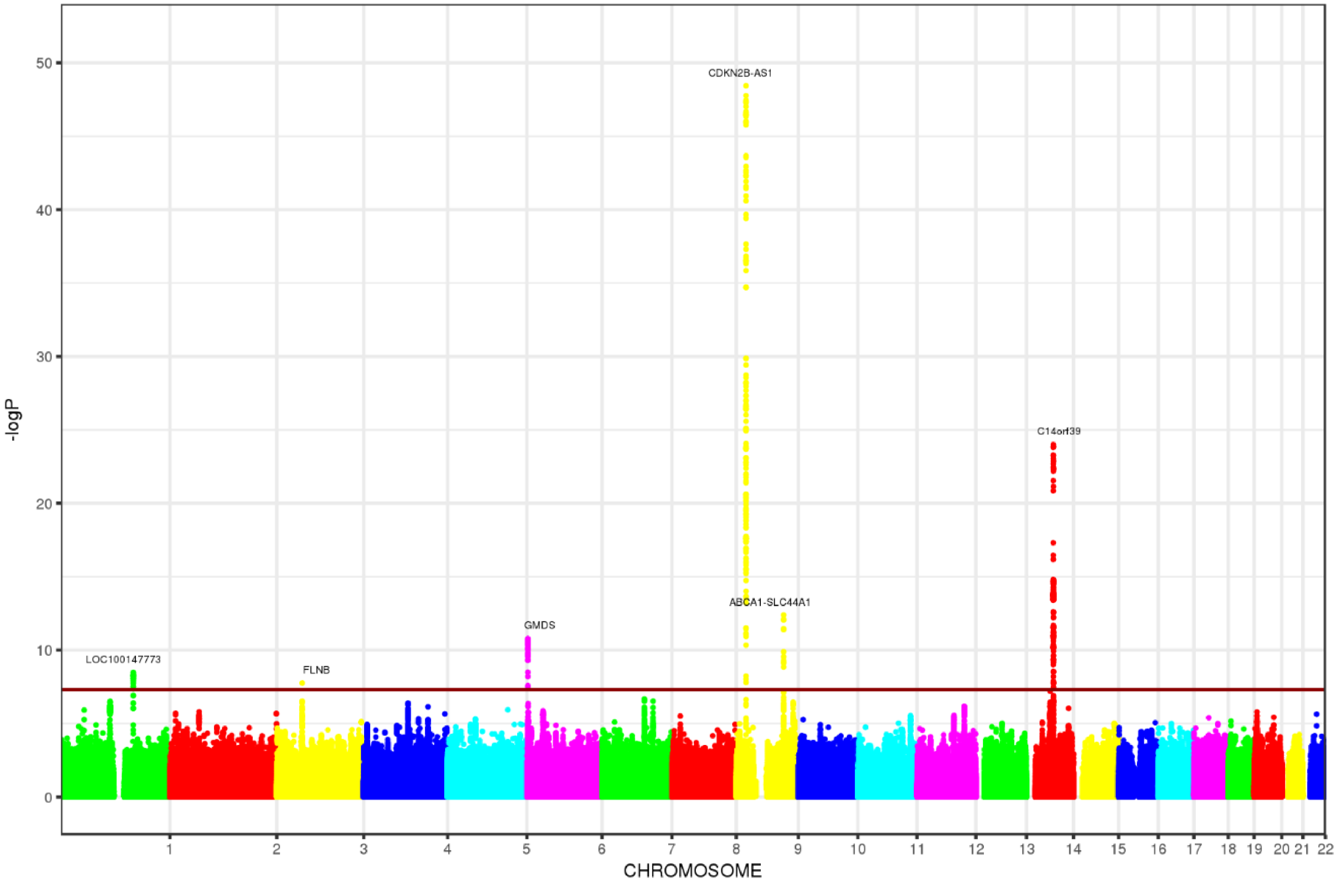

f) HTG-stratified meta-analysis (5,144 cases vs 47,997 controls)

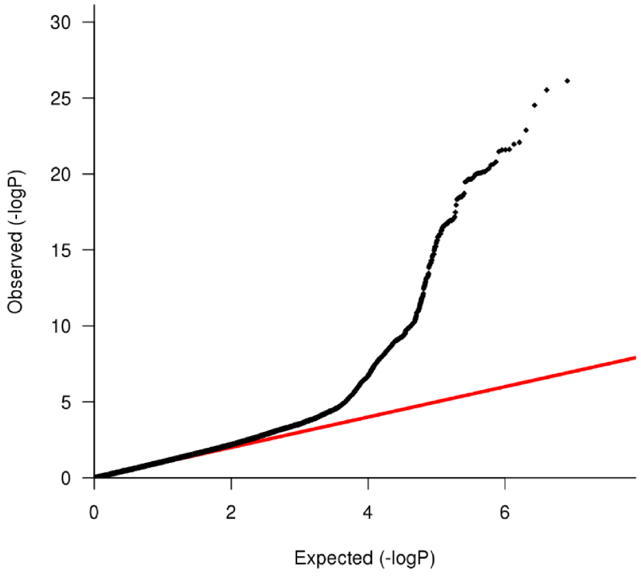

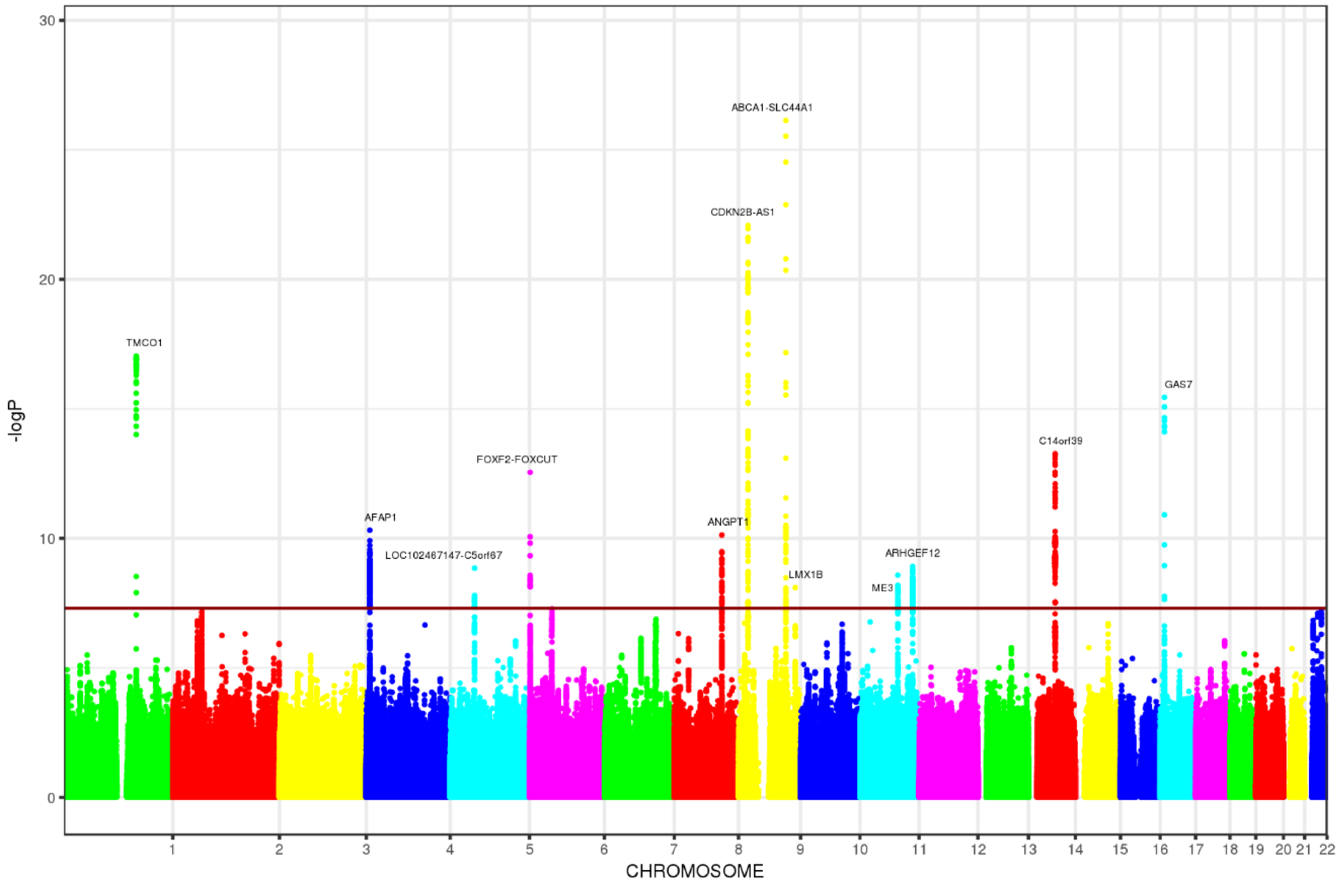

Supplementary Figure 2. Correlation of SNP effect estimates between European POAG meta-analysis and each of the replication datasets. The X-axis shows effect estimates in log(OR) scale for the European genome-wide significant SNPs (GWAS of 16,677 POAG cases vs 199,580 controls). The Y-axis shows estimates for the same SNPs in UKBB self-reports (GWAS of 7,286 cases vs 107,362 controls; panel A), Asians (GWAS of 6,935 POAG cases vs 39,588 controls; panel B), and Africans (GWAS of 3,281 POAG cases vs 2,791 controls; panel C). The previously known risk loci are shown in red and the previously unknown loci in blue. Horizontal grey bars on each dot represent the 95% confidence intervals (CIs; mean values +/- 1.96*SEM) for the effect estimates in Europeans, and vertical grey bars shows the 95% CIs in the replication datasets. The blue line is the linear regression line best fitting the data. The shaded area shows the 95% CIs on the repression line.

a) European POAG vs. UKBB self-reports

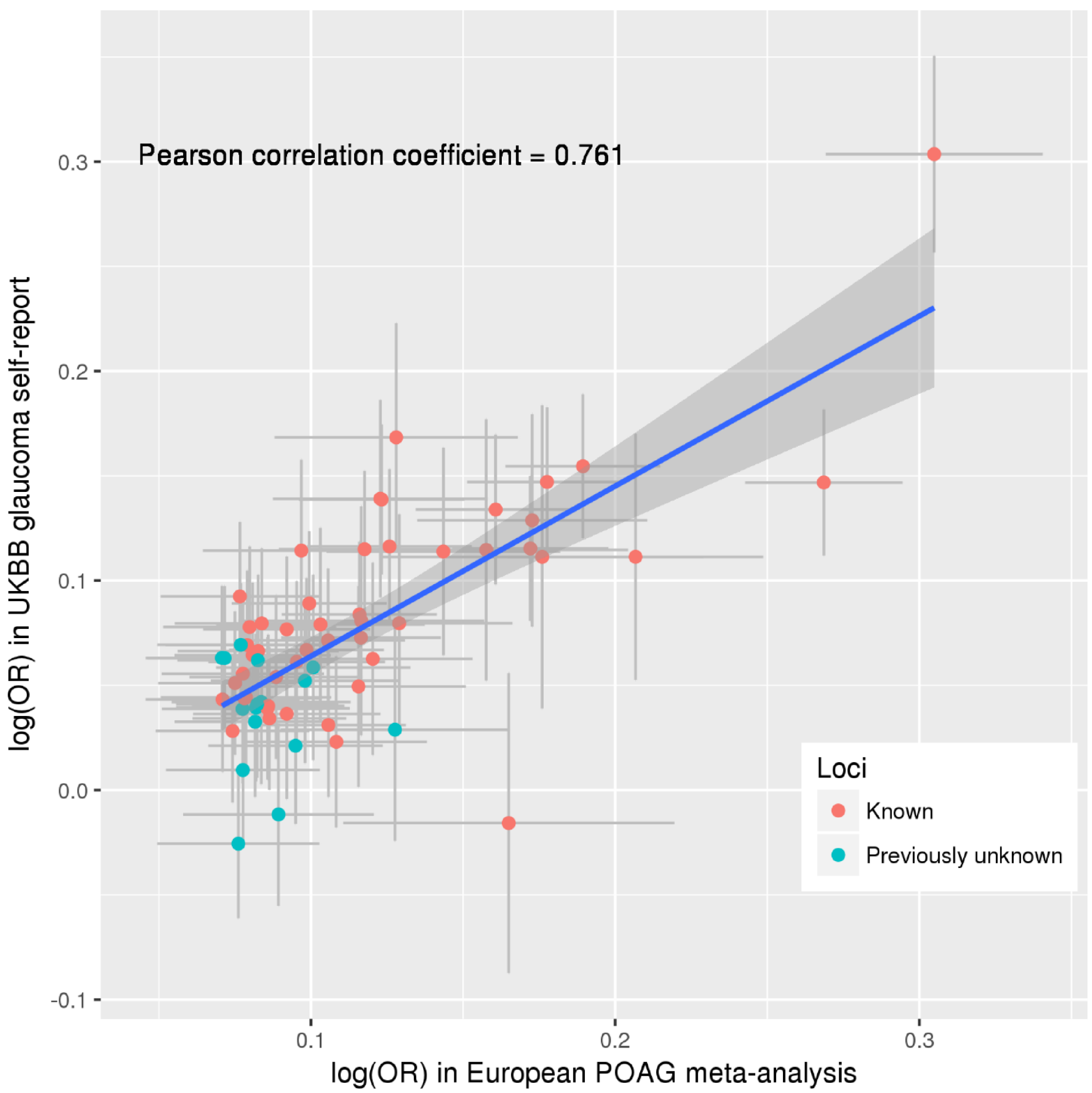

b) European POAG vs. Asians

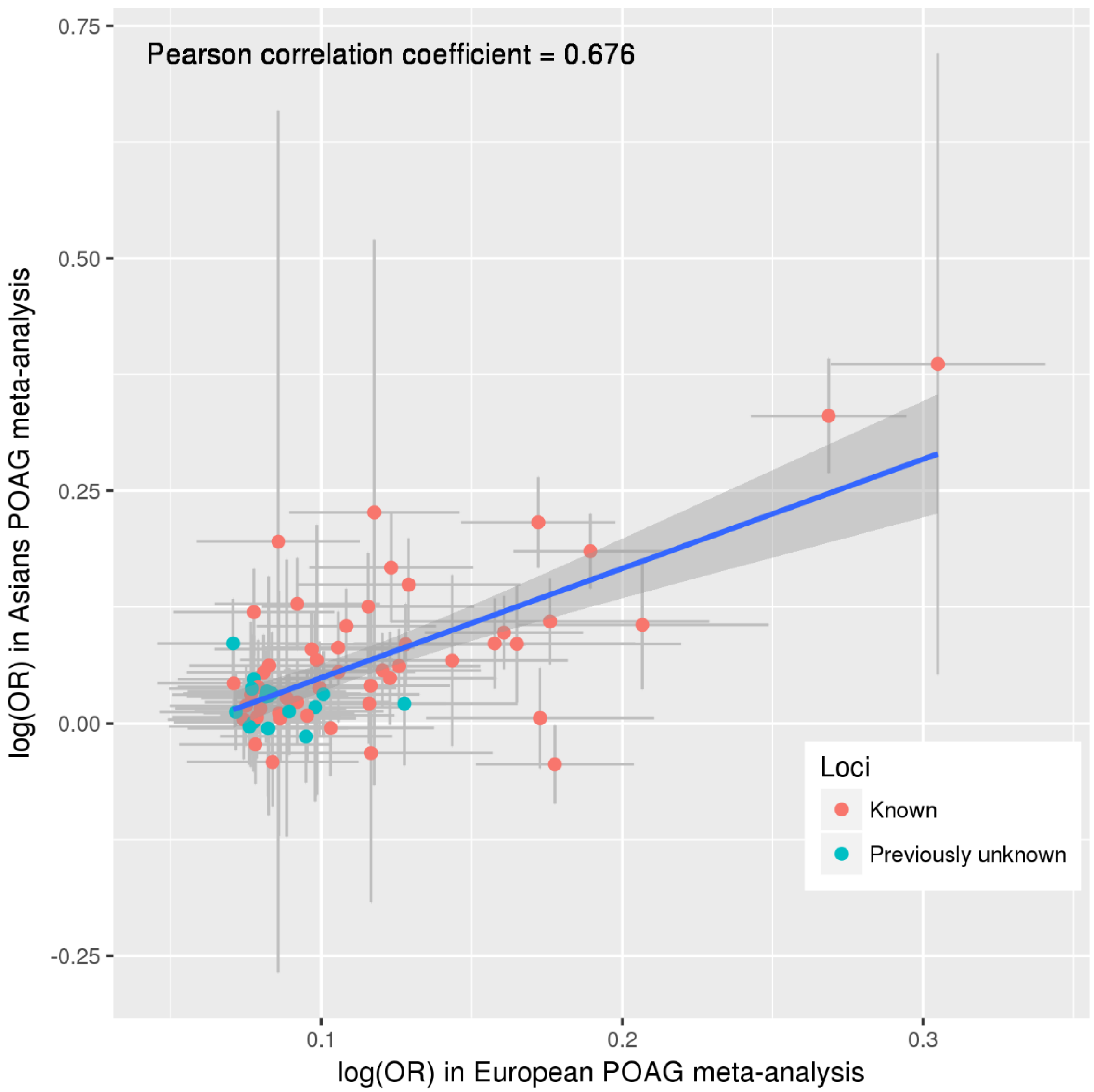

c) European POAG vs. Africans

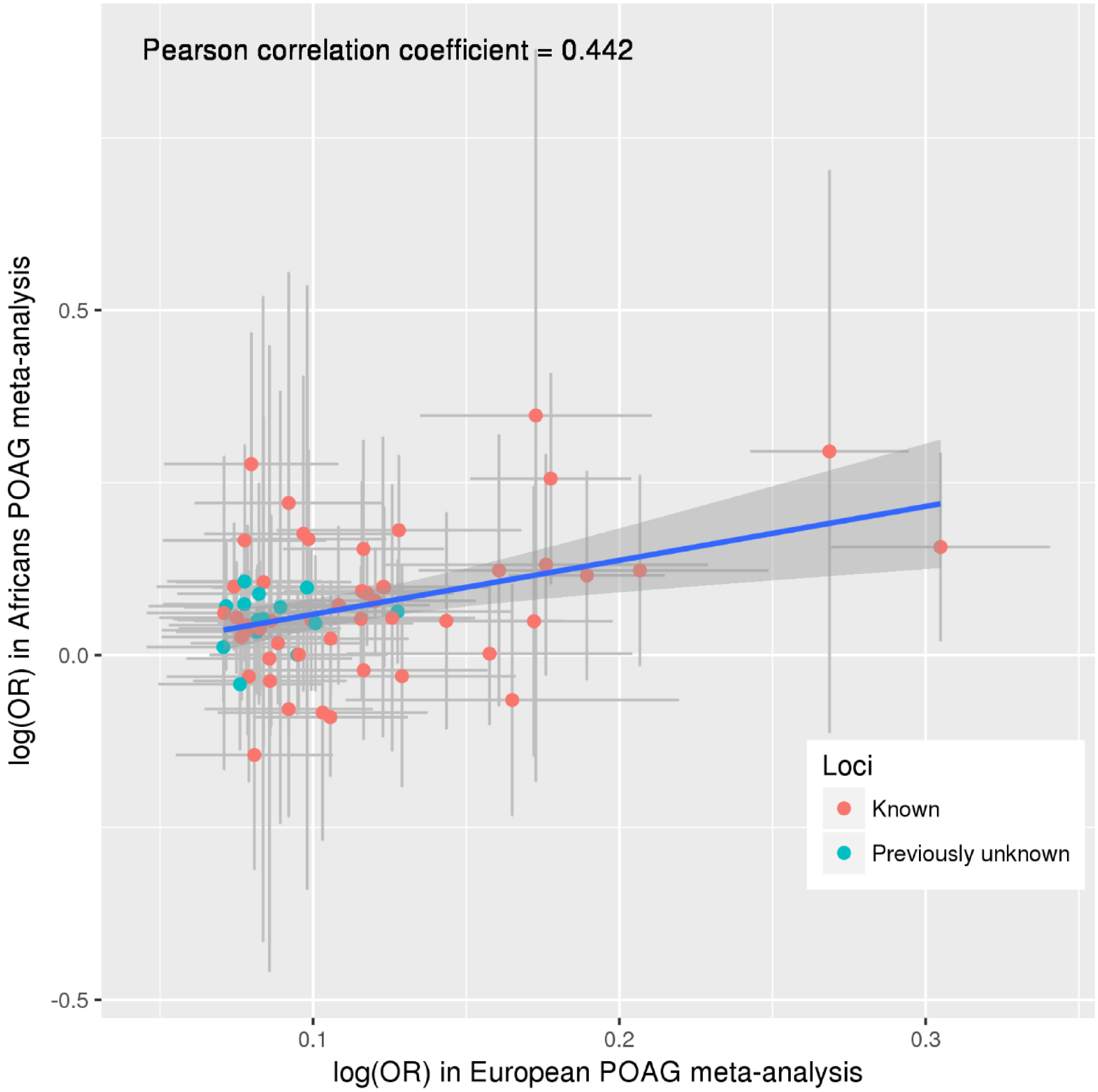

Supplementary Figure 3. LocusZoom plots for the genome-wide significant SNPs from the cross-ancestry meta-analysis. On each row, the plots show the same region in Europeans (first plot from the right), Asians (second plot), and Africans (third plot from the right). There are only two plots per row for the last four rows (X chromosome loci) because X chromosome data was not available for the African studies. The index SNPs are the most significant SNPs in the cross-ancestry meta-analysis while P-value of associations and LD structures are shown for each ancestry separately.

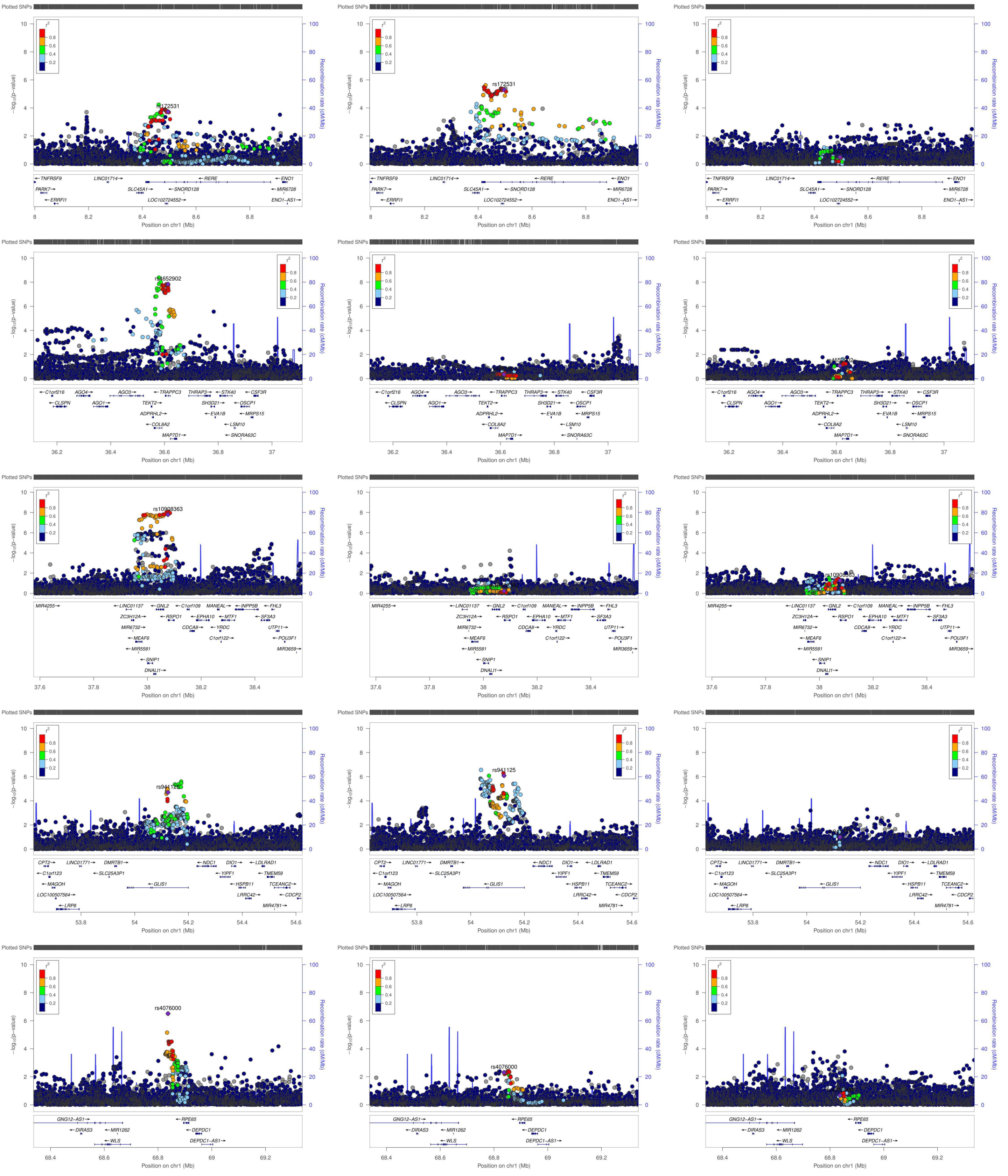

Plotted SNPs
−log10(p−value)
Recombination rate (cM/Mb)
r2
0.8
0.6
0.4
0.2
rs172531
TNFRSF9
PARK7
ERRFI1
LINC01714
SLC45A1
RERE
SNORD128
LOC102724552
ENO1
MIR6728
ENO1−AS1
Position on chr1 (Mb)
rs4652902
C1orf216
CLSPN
AGO4
AGO1
AGO3
TEKT2
ADPRHL2
COL8A2
TRAPPC3
MAP7D1
THRAP3
SH3D21
EVA1B
STK40
LSM10
OSCP1
MRPS15
CSF3R
SNORA63C
rs10908363
MIR4255
LINC01137
ZC3H12A
MIR6732
MEAF6
MIR5581
SNIP1
DNALI1
GNL2
RSPO1
C1orf109
CDCA8
EPHA10
MANEAL
YRDC
C1orf122
MTF1
INPP5B
SF3A3
FHL3
UTP11
POU3F1
MIR3659
rs941125
CPT2
C1orf123
MAGOH
LOC100507564
LRP8
LINC01771
DMRTB1
SLC25A3P1
GLIS1
NDC1
YIPF1
DIO1
HSPB11
LRRC42
LDLRAD1
TMEM59
TCEANC2
CDCP2
MIR4781
rs4076000
GNG12−AS1
DIRAS3
MIR1262
WLS
RPE65
DEPDC1
DEPDC1−AS1

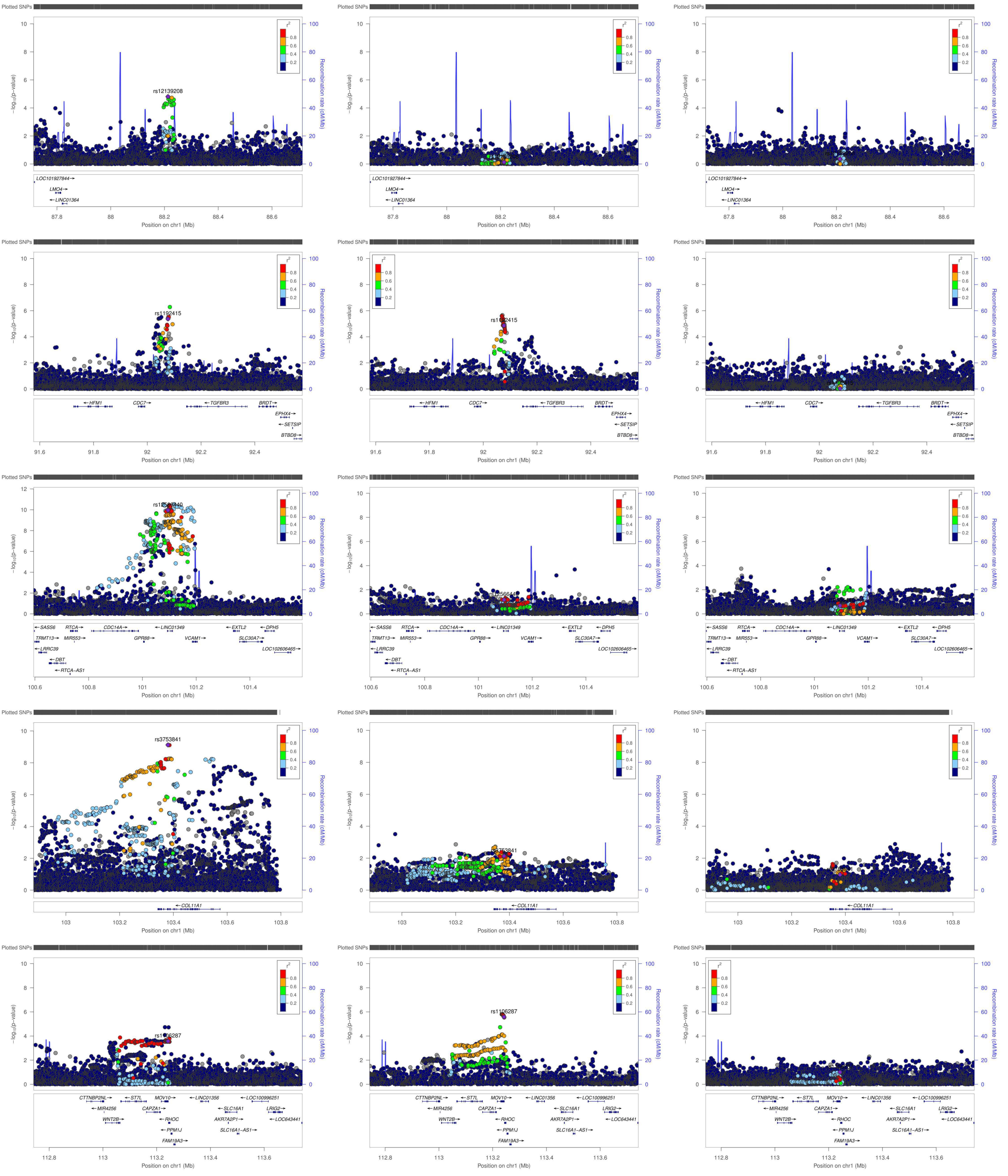

Plotted SNPs
−log10(p−value)
Recombination rate (cM/Mb)
r2
0.8
0.6
0.4
0.2
rs12139208
LOC101927844→
LMO4→
←LINC01364
Position on chr1 (Mb)
rs1192415
←HFM1
CDC7→
←TGFBR3
BRDT→
EPHX4→
←SETSIP
BTBD8→
rs12566440
←SASS6
RTCA→
CDC14A→
←LINC01349
←EXTL2
←DPH5
TRMT13→
MIR553→
GPR88→
VCAM1→
SLC30A7→
←LRRC39
LOC102606465→
←DBT
←RTCA−AS1
rs3753841
←COL11A1
rs1106287
CTTNBP2NL→
←ST7L
MOV10→
←LINC01356
←LOC100996251
←MIR4256
CAPZA1→
←SLC16A1
LRIG2→
WNT2B→
←RHOC
AKR7A2P1→
←LOC643441
←PPM1J
SLC16A1−AS1→
FAM19A3→

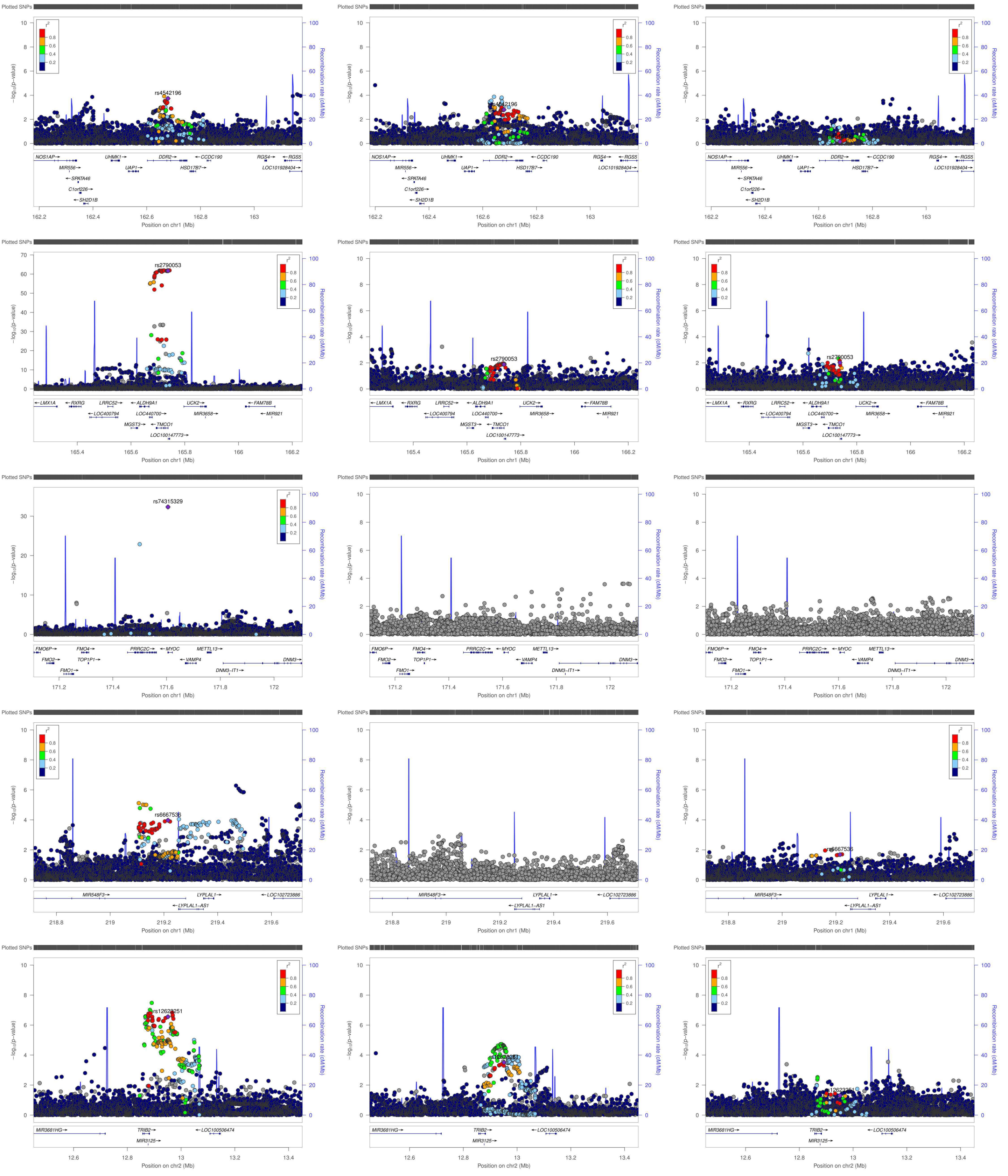

Plotted SNPs
−log10(p-value)
Recombination rate (cM/Mb)
r2
0.8
0.6
0.4
0.2
rs4542196
NOS1AP→
UHMK1→
DDR2→
←CCDC190
RGS4→
←RGS5
MIR556→
UAP1→
HSD17B7→
LOC101928404→
←SPATA46
C1orf226→
←SH2D1B
162.2
162.4
162.6
162.8
163
Position on chr1 (Mb)
rs2790053
←LMX1A
←RXRG
LRRC52→
←ALDH9A1
UCK2→
←FAM78B
←LOC400794
LOC440700→
MIR3658→
←MIR921
MGST3→
←TMCO1
LOC100147773→
165.4
165.6
165.8
166
166.2
rs74315329
FMO6P→
FMO4→
PRRC2C→
←MYOC
METTL13→
FMO2→
TOP1P1→
←VAMP4
DNM3→
FMO1→
DNM3−IT1→
171.2
171.4
171.6
171.8
172
rs6667536
MIR548F3→
LYPLAL1→
←LOC102723886
←LYPLAL1−AS1
218.8
219
219.2
219.4
219.6
rs12623251
MIR3681HG→
TRIB2→
←LOC100506474
MIR3125→
12.6
12.8
13
13.2
13.4
Position on chr2 (Mb)

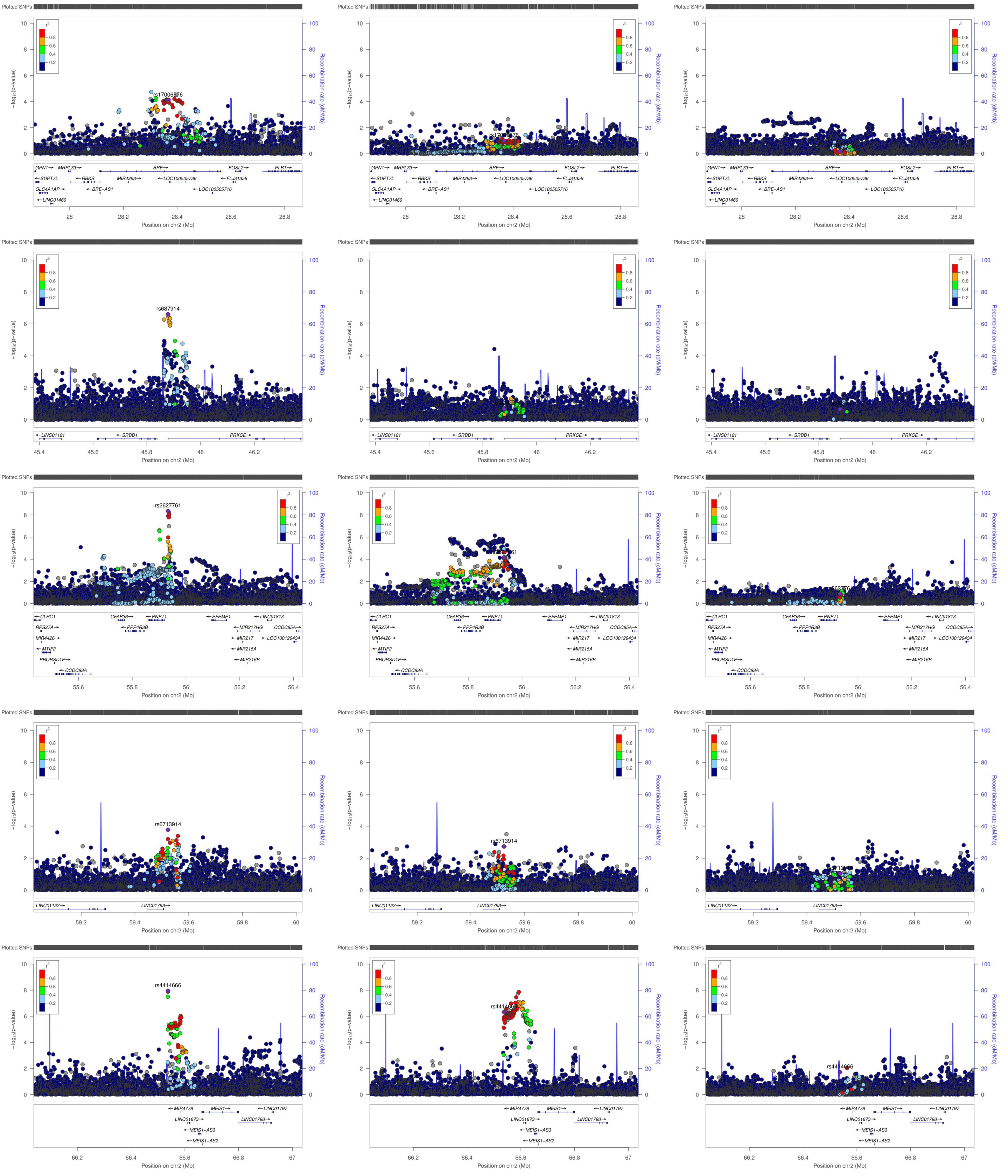

Plotted SNPs
−log10(p−value)
Recombination rate (cM/Mb)
r2
0.8
0.6
0.4
0.2
rs17006576
GPN1
MRPL33
BRE
FOSL2
PLB1
SUPT7L
RBKS
MIR4263
LOC100505736
FLJ31356
SLC4A1AP
BRE−AS1
LOC100505716
LINC01460
Position on chr2 (Mb)
rs687914
LINC01121
SRBD1
PRKCE
rs2627761
CLHC1
CFAP36
PNPT1
EFEMP1
LINC01813
RPS27A
PPP4R3B
MIR217HG
CCDC85A
MIR4426
MIR217
LOC100129434
MTIF2
MIR216A
PRORSD1P
MIR216B
CCDC88A
rs6713914
LINC01122
LINC01793
rs4414666
MIR4778
MEIS1
LINC01797
LINC01873
LINC01798
MEIS1−AS3
MEIS1−AS2

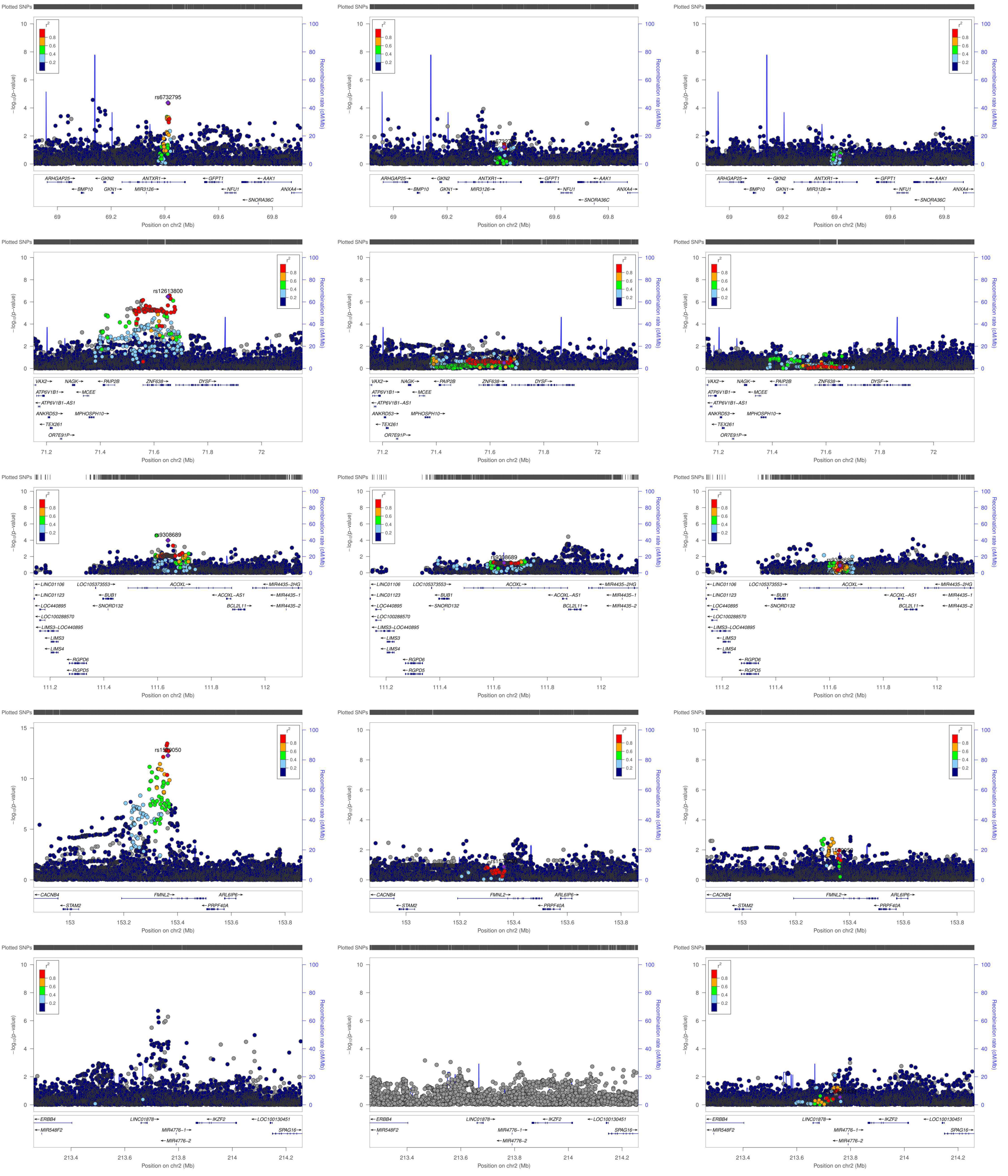

Plotted SNPs
−log10(p−value)
Recombination rate (cM/Mb)
r2
0.8
0.6
0.4
0.2
rs6732795
ARHGAP25
GKN2
ANTXR1
GFPT1
AAK1
BMP10
GKN1
MIR3126
NFU1
ANXA4
SNORA36C
Position on chr2 (Mb)
rs12613800
VAX2
NAGK
PAIP2B
ZNF638
DYSF
ATP6V1B1
MCEE
ATP6V1B1−AS1
ANKRD53
MPHOSPH10
TEX261
OR7E91P
rs9308689
LINC01106
LOC105373553
ACOXL
MIR4435−2HG
LINC01123
BUB1
ACOXL−AS1
MIR4435−1
LOC440895
SNORD132
BCL2L11
MIR4435−2
LOC100288570
LIMS3−LOC440895
LIMS3
LIMS4
RGPD6
RGPD5
rs1579050
CACNB4
FMNL2
ARL6IP6
STAM2
PRPF40A
ERBB4
LINC01878
IKZF2
LOC100130451
MIR548F2
MIR4776−1
SPAG16
MIR4776−2

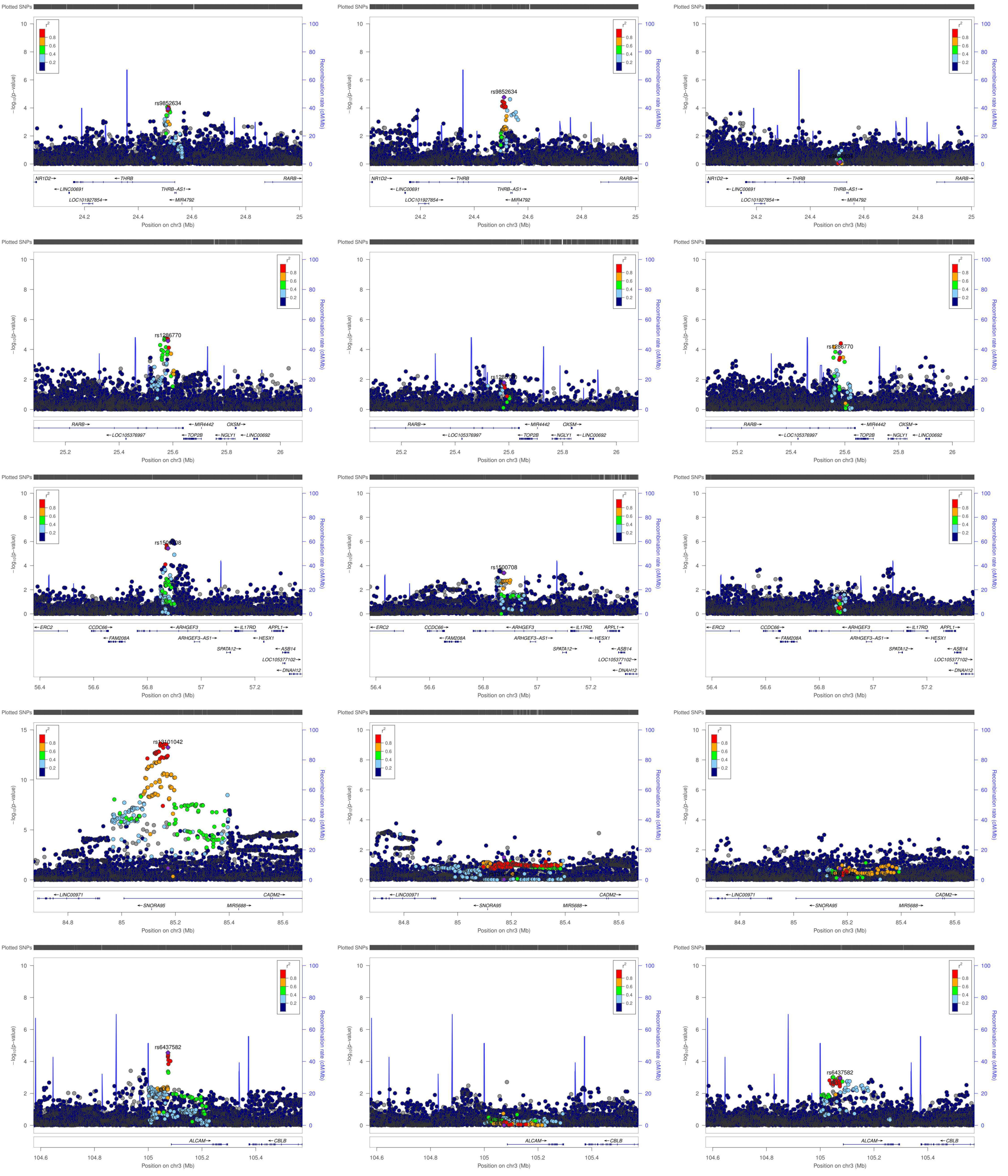

Plotted SNPs
−log10(p−value)
Recombination rate (cM/Mb)
r2
0.8
0.6
0.4
0.2
rs9852634
NR1D2→
←THRB
RARB→
←LINC00691
THRB−AS1→
LOC101927854→
←MIR4792
Position on chr3 (Mb)
rs1286770
RARB→
←MIR4442
OXSM→
←LOC105376997
←TOP2B
←NGLY1
←LINC00692
rs1500708
←ERC2
CCDC66→
←ARHGEF3
←IL17RD
APPL1→
←FAM208A
ARHGEF3−AS1→
←HESX1
SPATA12→
←ASB14
LOC105377102→
←DNAH12
rs13101042
←LINC00971
CADM2→
←SNORA95
MIR5688→
rs6437582
ALCAM→
←CBLB

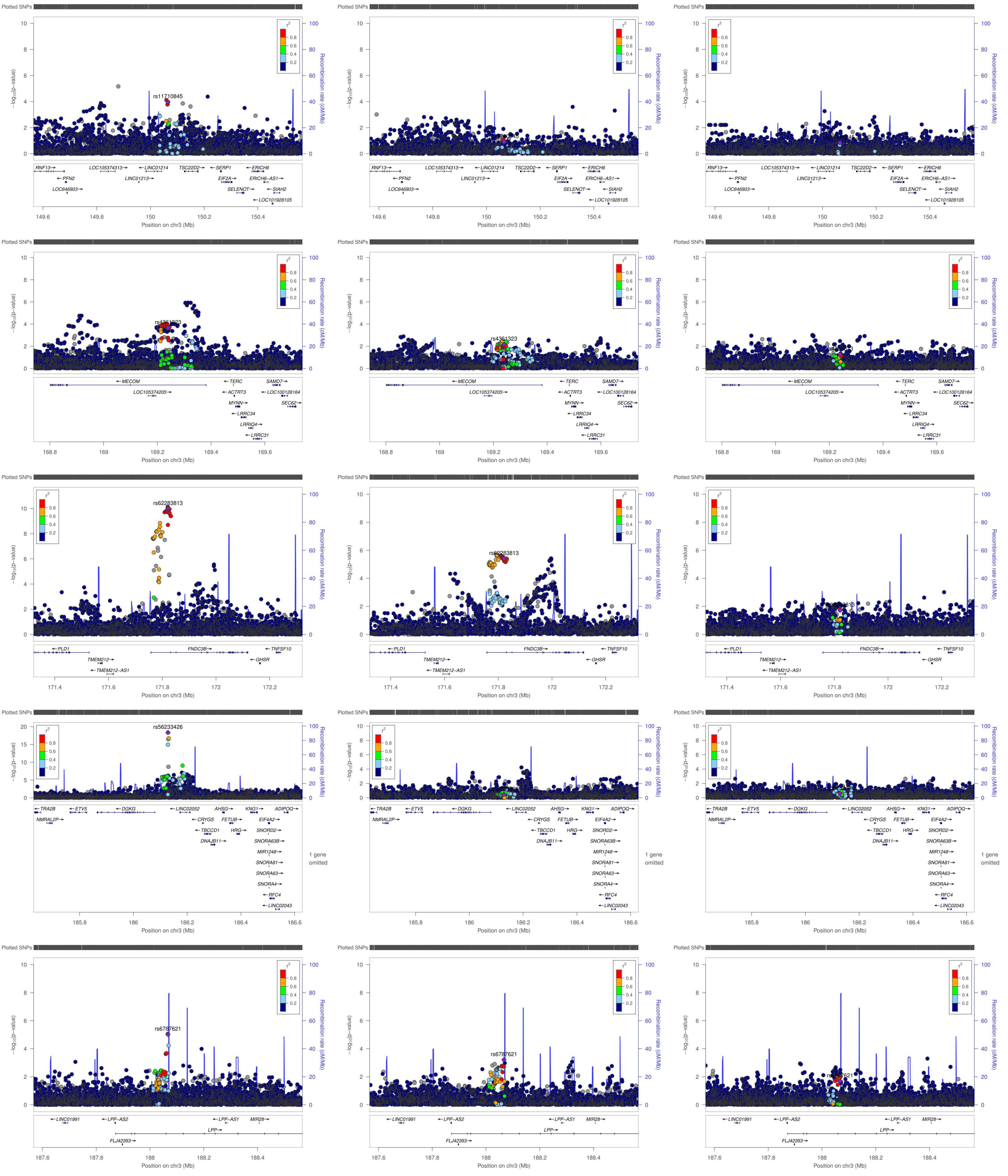

Plotted SNPs
−log10(p-value)
Recombination rate (cM/Mb)
r2
0.8
0.6
0.4
0.2
rs11710845
RNF13→
PFN2
LOC646903→
LOC105374313→
LINC01213→
LINC01214
TSC22D2→
SERP1
EIF2A→
SELENOT→
ERICH6
ERICH6−AS1→
SIAH2
LOC101928105
149.6
149.8
150
150.2
150.4
Position on chr3 (Mb)
rs4361323
MECOM
LOC105374205→
TERC
ACTRT3
MYNN→
LRRC34
LRRIQ4→
LRRC31
SAMD7→
LOC100128164
SEC62→
168.8
169
169.2
169.4
169.6
Position on chr3 (Mb)
rs62283813
PLD1
TMEM212→
TMEM212−AS1
FNDC3B→
GHSR
TNFSF10
171.4
171.6
171.8
172
172.2
Position on chr3 (Mb)
rs56233426
TRA2B
NMRAL2P→
ETV5
DGKG
LINC02052
CRYGS
TBCCD1
DNAJB11→
AHSG→
FETUB→
HRG→
KNG1→
EIF4A2→
SNORD2→
SNORA63B→
MIR1248→
SNORA81→
SNORA63→
SNORA4→
RFC4
LINC02043
ADIPOQ→
1 gene omitted
185.8
186
186.2
186.4
186.6
Position on chr3 (Mb)
rs6787621
LINC01991
LPP−AS2
FLJ42393→
LPP→
LPP−AS1
MIR28→
187.6
187.8
188
188.2
188.4
Position on chr3 (Mb)

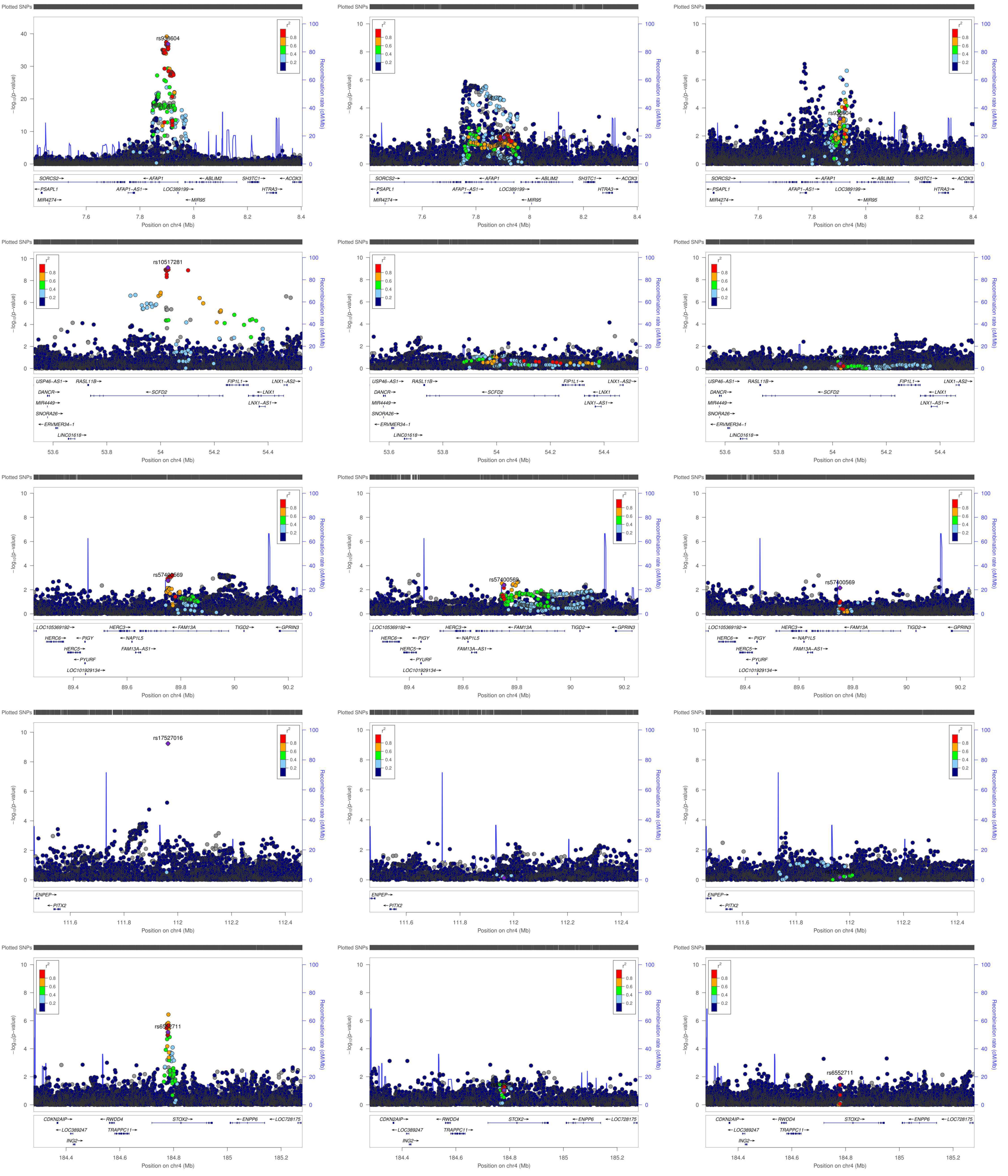

Plotted SNPs
−log10(p−value)
Recombination rate (cM/Mb)
r2
0.8
0.6
0.4
0.2
rs938604
SORCS2→
←AFAP1
←ABLIM2
SH3TC1→
←ACOX3
←PSAPL1
AFAP1−AS1→
LOC389199→
HTRA3→
MIR4274→
←MIR95
Position on chr4 (Mb)
rs10517281
USP46−AS1→
RASL11B→
FIP1L1→
LNX1−AS2→
DANCR→
←SCFD2
←LNX1
MIR4449→
LNX1−AS1→
SNORA26→
←ERVMER34−1
LINC01618→
rs57400569
LOC105369192→
HERC3→
←FAM13A
TIGD2→
←GPRIN3
HERC6→
←PIGY
←NAP1L5
HERC5→
FAM13A−AS1→
←PYURF
LOC101929134→
rs17527016
ENPEP→
←PITX2
rs6552711
CDKN2AIP→
←RWDD4
STOX2→
←ENPP6
←LOC728175
←LOC389247
TRAPPC11→
ING2→

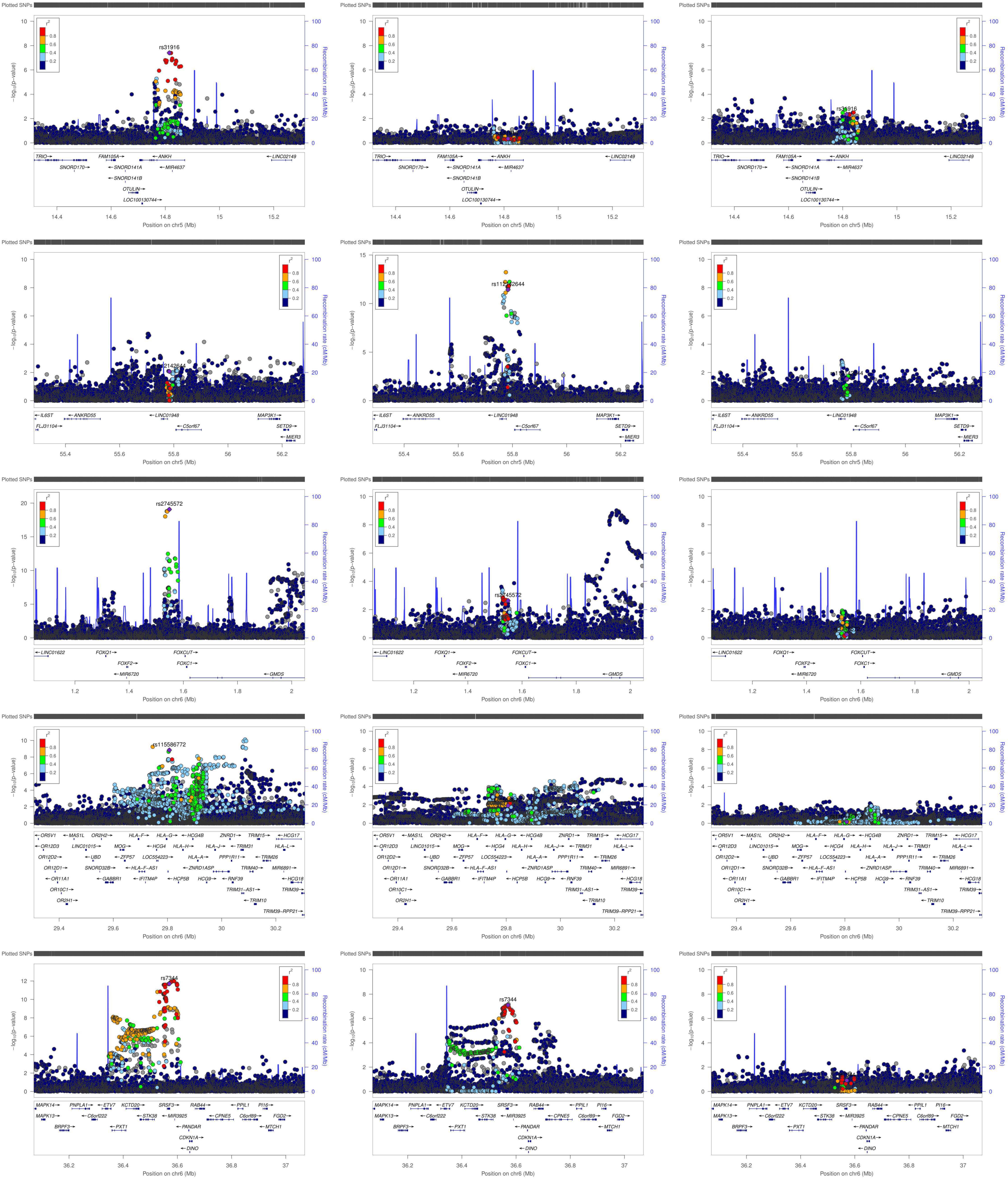

Plotted SNPs
r2
0.8
0.6
0.4
0.2
-log10(p-value)
Recombination rate (cM/Mb)
rs31916
TRIO
FAM105A
ANKH
LINC02149
SNORD170
SNORD141A
MIR4637
SNORD141B
OTULIN
LOC100130744
Position on chr5 (Mb)
rs112142644
IL6ST
ANKRD55
LINC01948
MAP3K1
FLJ31104
C5orf67
SETD9
MIER3
rs2745572
LINC01622
FOXQ1
FOXCUT
FOXF2
FOXC1
MIR6720
GMDS
Position on chr6 (Mb)
rs115586772
OR5V1
MAS1L
OR2H2
HLA-F
HLA-G
HCG4B
ZNRD1
TRIM15
HCG17
OR12D3
LINC01015
MOG
HCG4
HLA-H
HLA-J
TRIM31
HLA-L
OR12D2
UBD
ZFP57
LOC554223
HLA-A
PPP1R11
TRIM26
OR12D1
SNORD32B
HLA-F-AS1
ZNRD1ASP
TRIM40
MIR6891
OR11A1
GABBR1
IFITM4P
HCP5B
HCG9
RNF39
HCG18
OR10C1
TRIM31-AS1
TRIM39
OR2H1
TRIM10
TRIM39-RPP21
rs7344
MAPK14
PNPLA1
ETV7
KCTD20
SRSF3
RAB44
PPIL1
PI16
MAPK13
C6orf222
STK38
MIR3925
CPNE5
C6orf89
FGD2
BRPF3
PXT1
PANDAR
MTCH1
CDKN1A
DINO

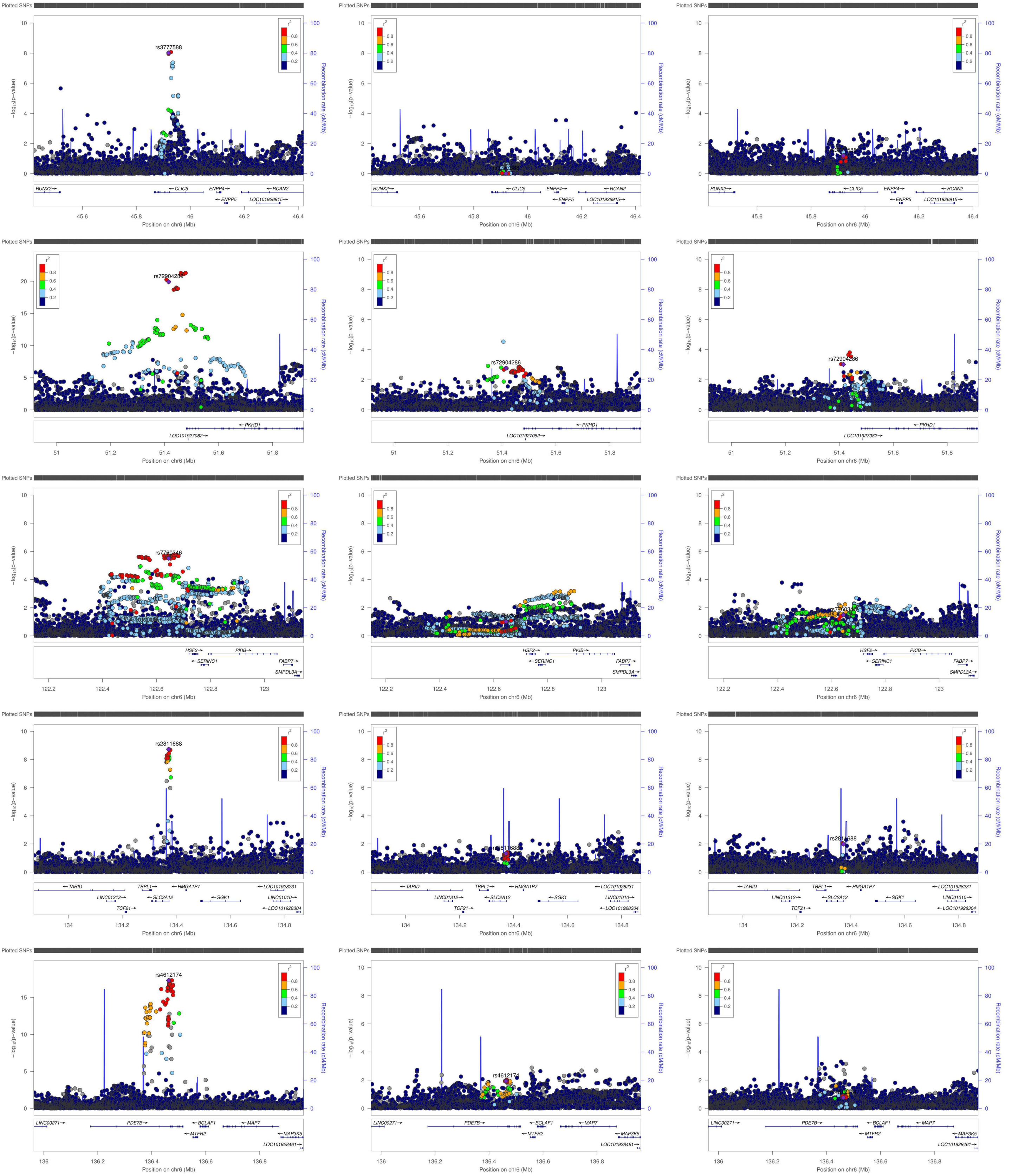
Plotted SNPs
−log10(p−value)
Recombination rate (cM/Mb)
r2
0.8
0.6
0.4
0.2
rs3777588
RUNX2
CLIC5
ENPP4
RCAN2
ENPP5
LOC101926915
Position on chr6 (Mb)
rs72904286
PKHD1
LOC101927082
rs7760346
HSF2
PKIB
SERINC1
FABP7
SMPDL3A
rs2811688
TARID
TBPL1
HMGA1P7
LOC101928231
LINC01312
SLC2A12
SGK1
LINC01010
TCF21
LOC101928304
rs4612174
LINC00271
PDE7B
BCLAF1
MAP7
MTFR2
MAP3K5
LOC101928461

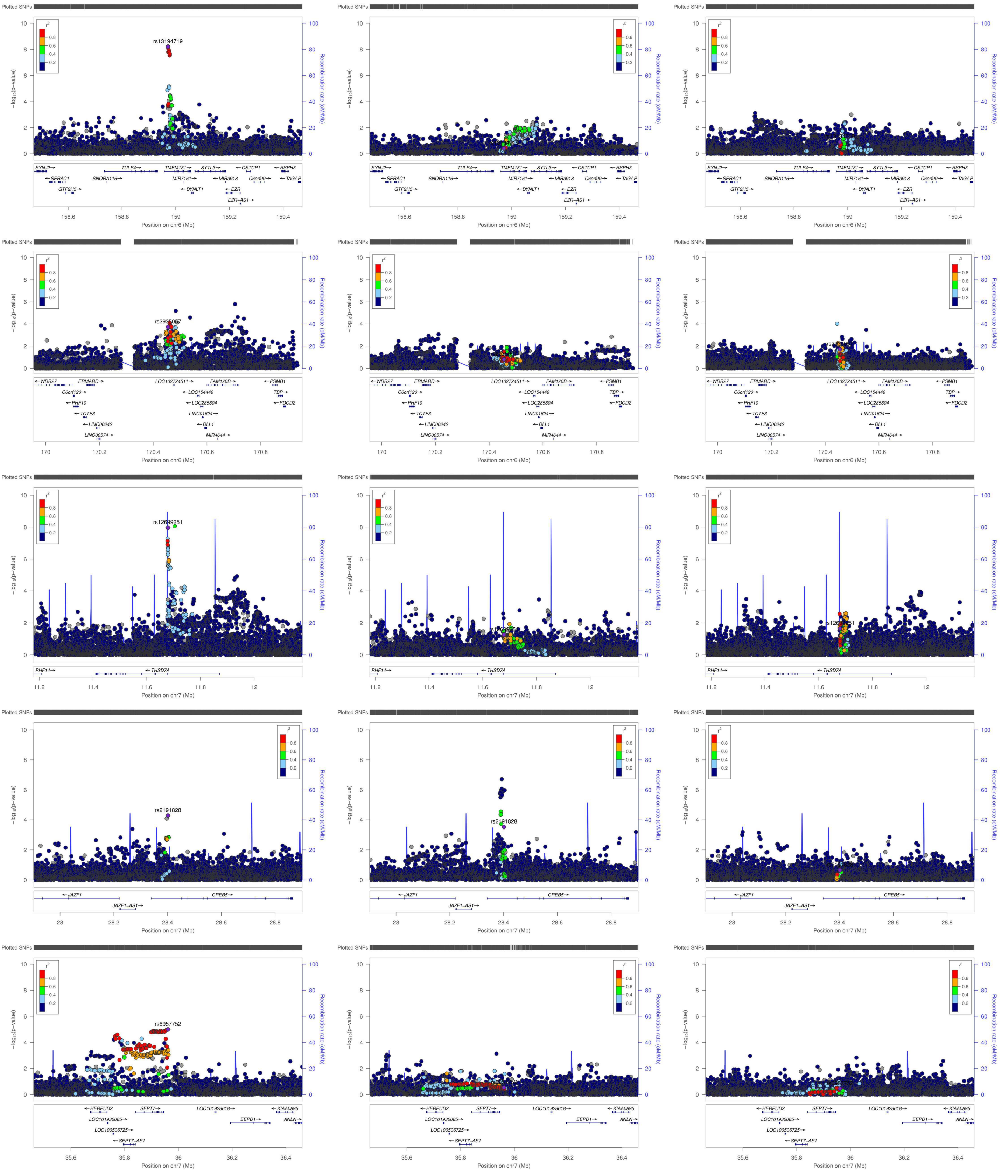
Plotted SNPs
−log10(p−value)
Recombination rate (cM/Mb)
r2
0.8
0.6
0.4
0.2
rs13194719
SYNJ2 SERAC1 GTF2H5 TULP4 SNORA116 TMEM181 MIR7161 DYNLT1 SYTL3 MIR3918 OSTCP1 EZR EZR−AS1 C6orf99 RSPH3 TAGAP
Position on chr6 (Mb)
rs2935057
WDR27 ERMARD C6orf120 PHF10 TCTE3 LINC00242 LINC00574 LOC102724511 LOC154449 LOC285804 LINC01624 DLL1 MIR4644 FAM120B PSMB1 TBP PDCD2
Position on chr6 (Mb)
rs12699251
PHF14 THSD7A
Position on chr7 (Mb)
rs2191828
JAZF1 JAZF1−AS1 CREB5
Position on chr7 (Mb)
rs6957752
HERPUD2 LOC101930085 LOC100506725 SEPT7 SEPT7−AS1 LOC101928618 EEPD1 KIAA0895 ANLN
Position on chr7 (Mb)

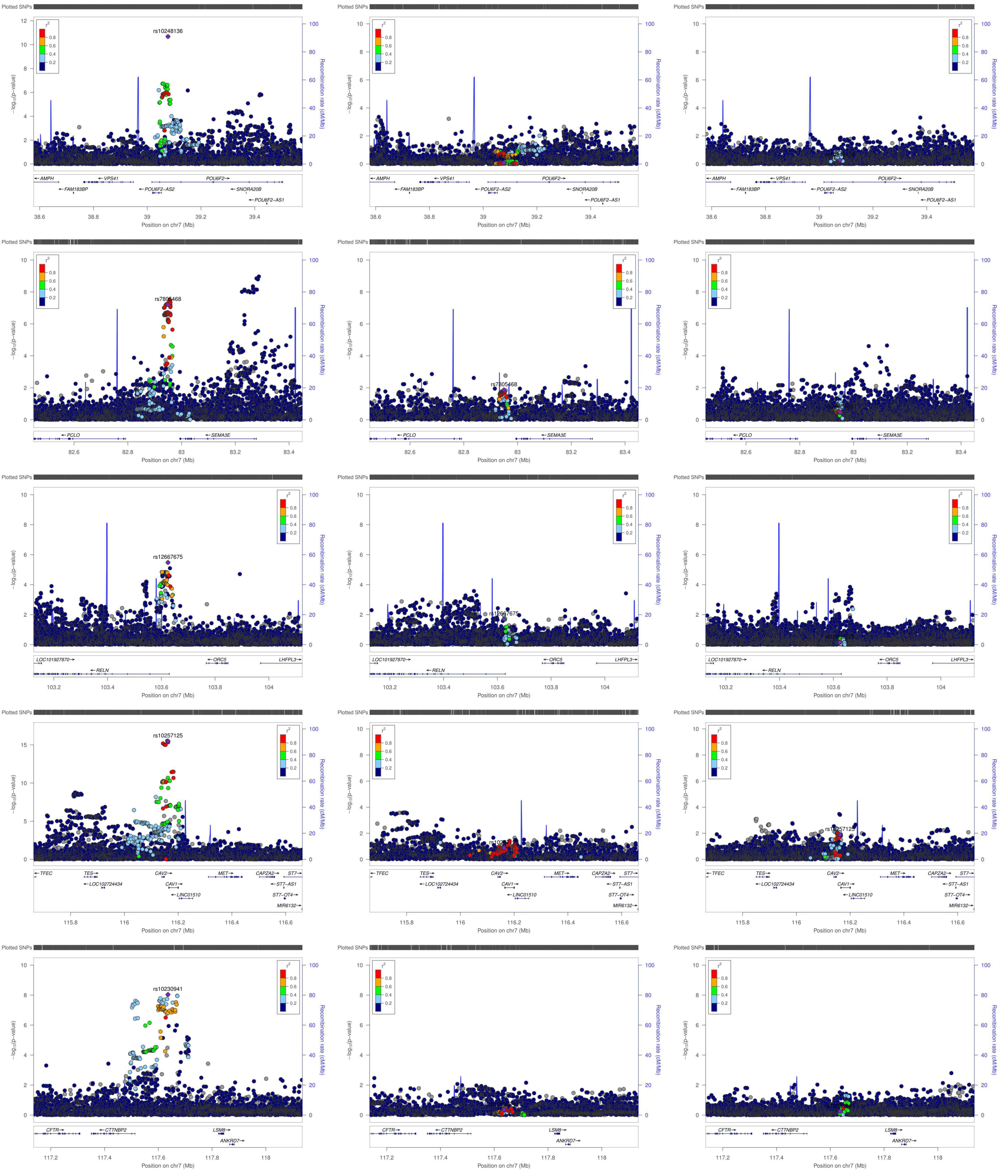

Plotted SNPs
rs10248136
rs7805468
rs12667675
rs10257125
rs10230941
−log10(p-value)
Recombination rate (cM/Mb)
r2
Position on chr7 (Mb)
AMPH
VPS41
POU6F2
FAM183BP
POU6F2-AS2
SNORA20B
POU6F2-AS1
PCLO
SEMA3E
LOC101927870
RELN
ORC5
LHFPL3
TFEC
TES
CAV2
MET
CAPZA2
ST7
LOC102724434
CAV1
ST7-AS1
LINC01510
ST7-OT4
MIR6132
CFTR
CTTNBP2
LSM8
ANKRD7

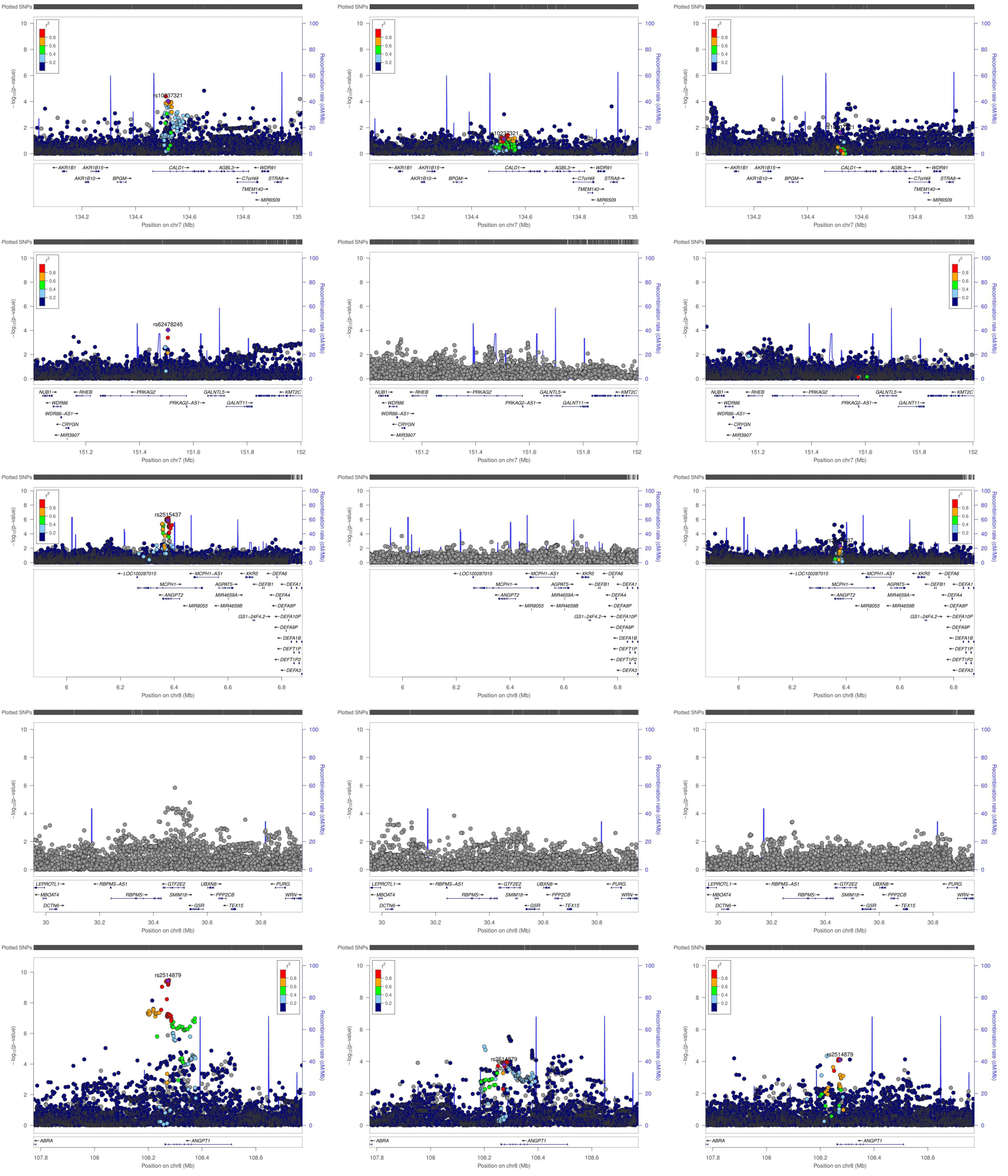

Plotted SNPs
−log10(p−value)
Recombination rate (cM/Mb)
r2
0.8
0.6
0.4
0.2
rs10237321
AKR1B1
AKR1B15
AKR1B10
BPGM
CALD1
AGBL3
WDR91
C7orf49
STRA8
TMEM140
MIR6509
134.2
134.4
134.6
134.8
135
Position on chr7 (Mb)
rs62478245
NUB1
RHEB
PRKAG2
GALNTL5
KMT2C
WDR86
PRKAG2−AS1
GALNT11
WDR86−AS1
CRYGN
MIR3907
151.2
151.4
151.6
151.8
152
Position on chr7 (Mb)
rs2515437
LOC100287015
MCPH1−AS1
XKR5
DEFA6
MCPH1
AGPAT5
DEFB1
DEFA1
ANGPT2
MIR4659A
DEFA4
MIR8055
MIR4659B
DEFA8P
GS1−24F4.2
DEFA10P
DEFA9P
DEFA1B
DEFT1P
DEFT1P2
DEFA3
6
6.2
6.4
6.6
6.8
Position on chr8 (Mb)
LEPROTL1
RBPMS−AS1
GTF2E2
UBXN8
PURG
MBOAT4
RBPMS
SMIM18
PPP2CB
WRN
DCTN6
GSR
TEX15
30
30.2
30.4
30.6
30.8
Position on chr8 (Mb)
rs2514879
ABRA
ANGPT1
107.8
108
108.2
108.4
108.6
Position on chr8 (Mb)

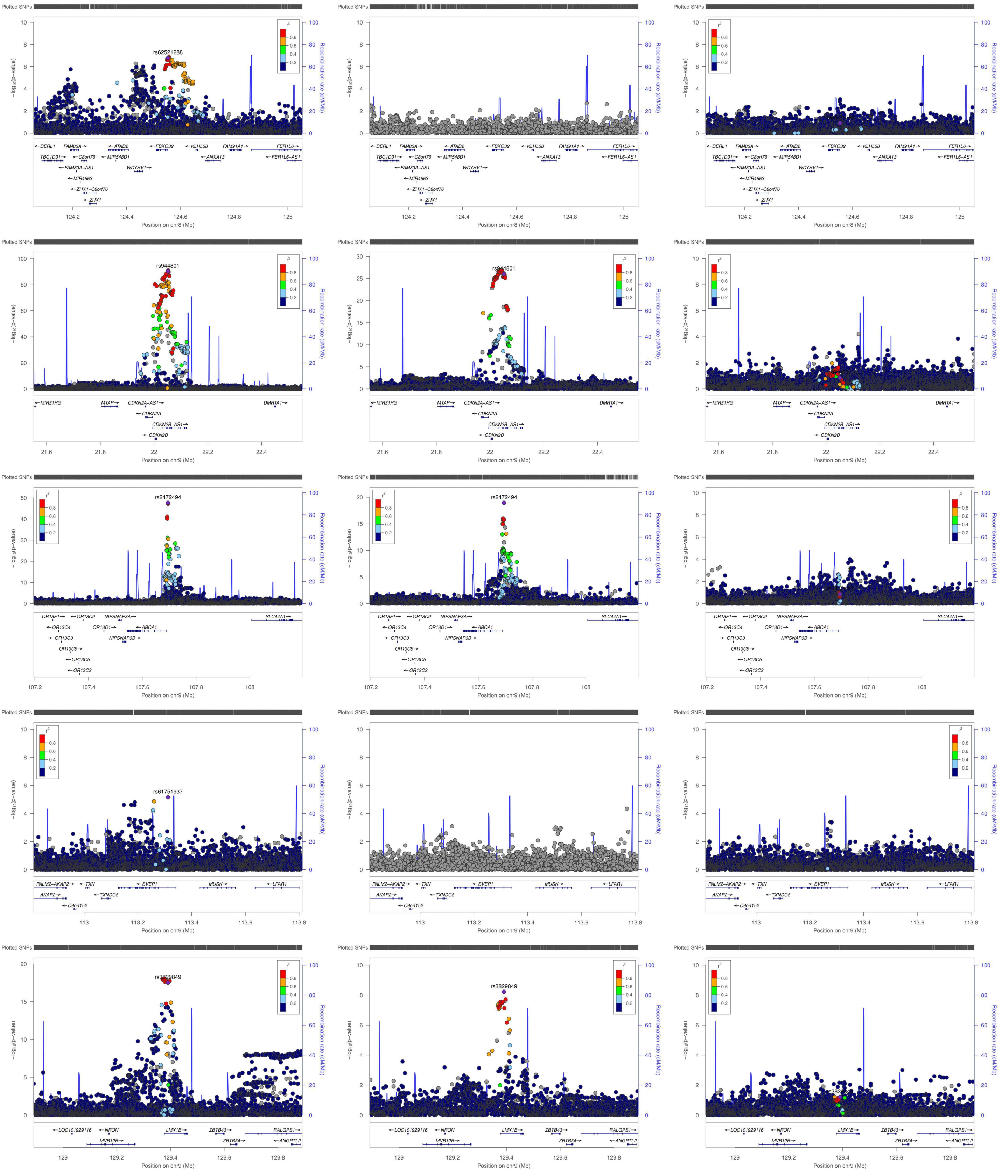
Plotted SNPs
−log10(p-value)
Recombination rate (cM/Mb)
r2
0.8
0.6
0.4
0.2
rs62521288
DERL1
FAM83A
ATAD2
FBXO32
KLHL38
FAM91A1
FER1L6
TBC1D31
C8orf76
MIR548D1
ANXA13
FER1L6-AS1
FAM83A-AS1
WDYHV1
MIR4663
ZHX1-C8orf76
ZHX1
Position on chr8 (Mb)
rs944801
MIR31HG
MTAP
CDKN2A-AS1
DMRTA1
CDKN2A
CDKN2B-AS1
CDKN2B
Position on chr9 (Mb)
rs2472494
OR13F1
OR13C9
NIPSNAP3A
SLC44A1
OR13C4
OR13D1
ABCA1
OR13C3
NIPSNAP3B
OR13C8
OR13C5
OR13C2
rs61751937
PALM2-AKAP2
TXN
SVEP1
MUSK
LPAR1
AKAP2
TXNDC8
C9orf152
rs3829849
LOC101929116
NRON
LMX1B
ZBTB43
RALGPS1
MVB12B
ZBTB34
ANGPTL2

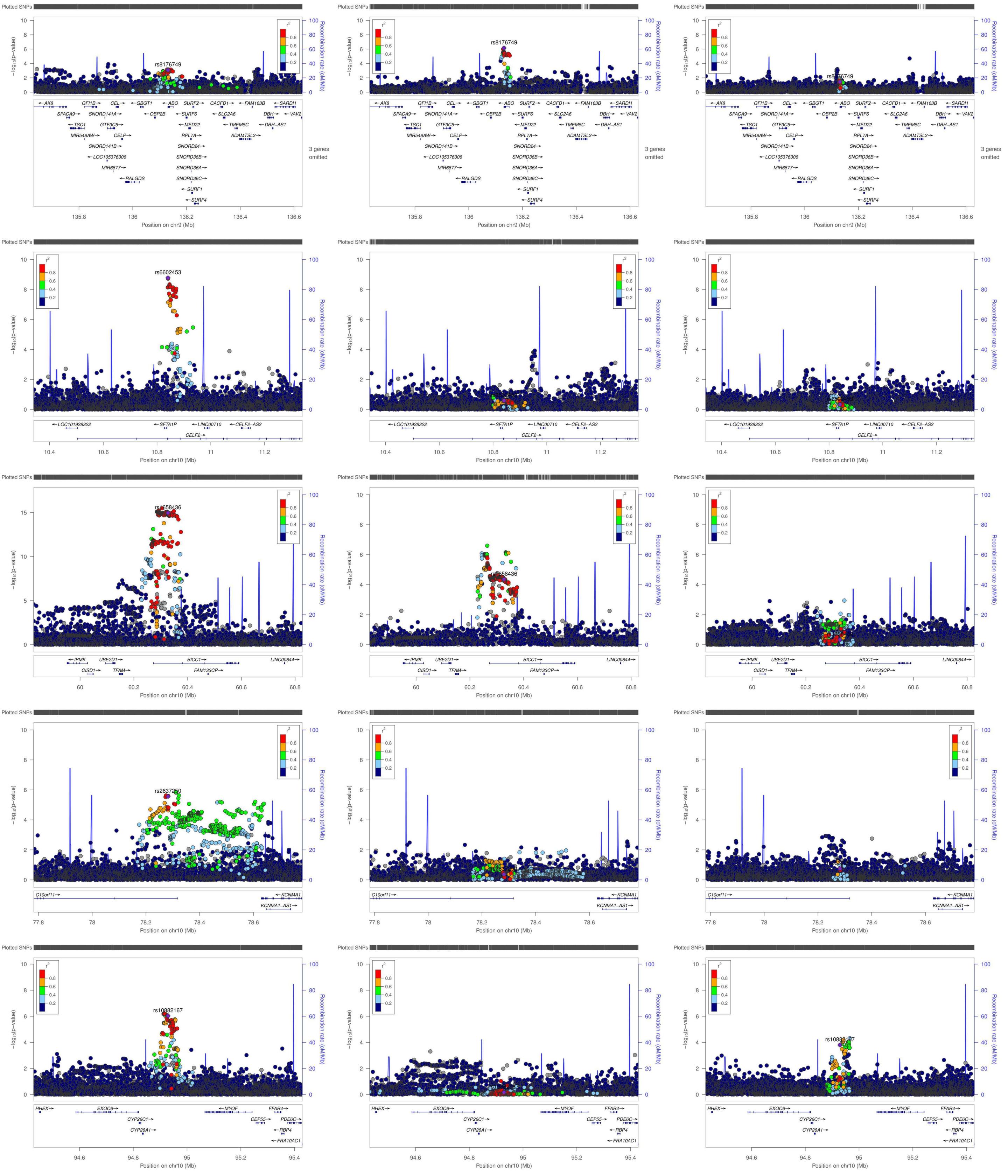

Plotted SNPs
−log10(p−value)
Recombination rate (cM/Mb)
r2
0.8
0.6
0.4
0.2
rs8176749
AK8
GFI1B
CEL
GBGT1
ABO
SURF2
CACFD1
FAM163B
SARDH
SPACA9
SNORD141A
OBP2B
SURF6
SLC2A6
DBH
VAV2
TSC1
GTF3C5
MED22
TMEM8C
DBH−AS1
MIR548AW
CELP
RPL7A
ADAMTSL2
SNORD141B
SNORD24
LOC105376306
SNORD36B
MIR6877
SNORD36A
RALGDS
SNORD36C
SURF1
SURF4
3 genes omitted
135.8
136
136.2
136.4
136.6
Position on chr9 (Mb)
rs6602453
LOC101928322
SFTA1P
LINC00710
CELF2−AS2
CELF2
10.4
10.6
10.8
11
11.2
Position on chr10 (Mb)
rs1658436
IPMK
UBE2D1
BICC1
LINC00844
CISD1
TFAM
FAM133CP
60
60.2
60.4
60.6
60.8
Position on chr10 (Mb)
rs2637250
C10orf11
KCNMA1
KCNMA1−AS1
77.8
78
78.2
78.4
78.6
Position on chr10 (Mb)
rs10882167
HHEX
EXOC6
MYOF
FFAR4
CYP26C1
CEP55
PDE6C
CYP26A1
RBP4
FRA10AC1
94.6
94.8
95
95.2
95.4
Position on chr10 (Mb)

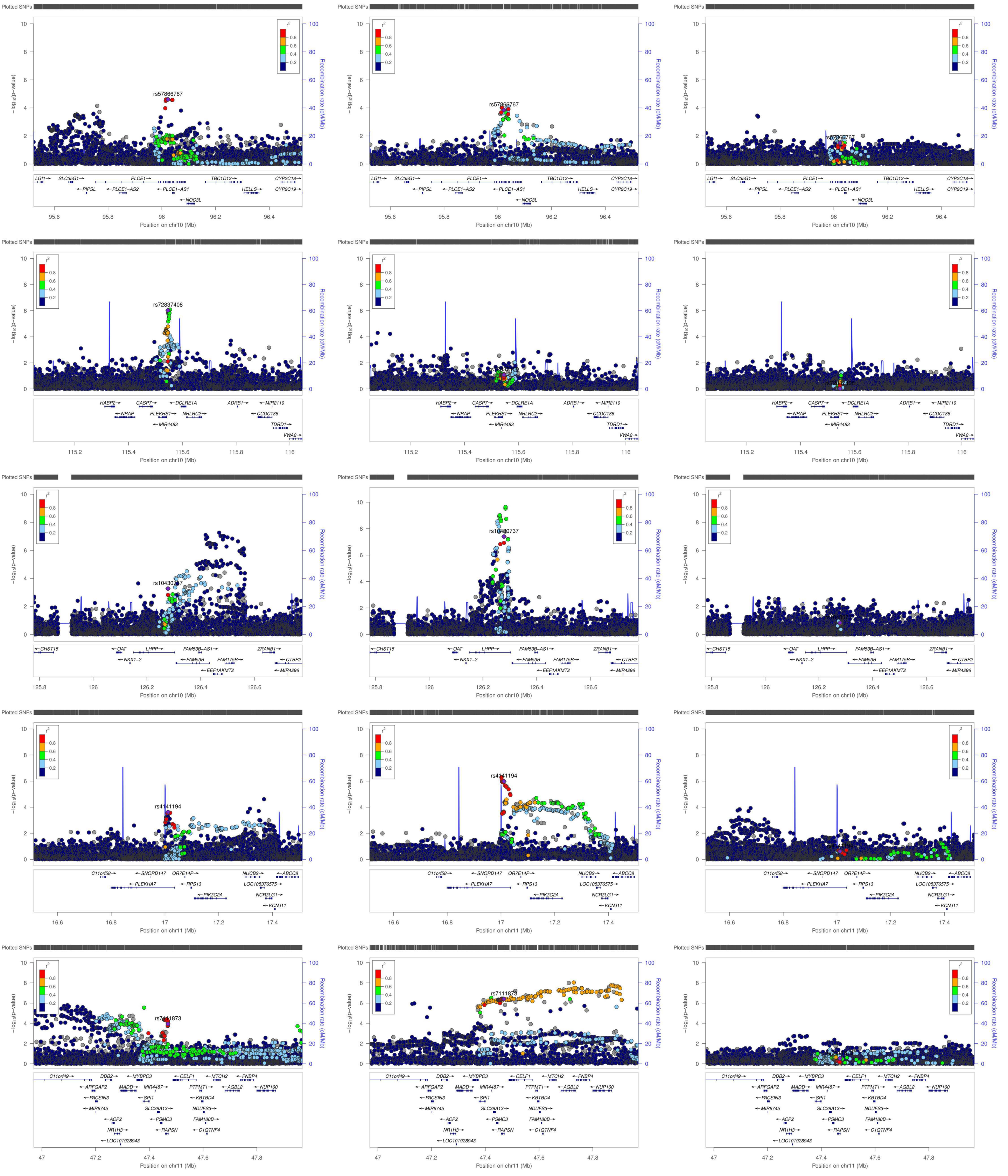
Plotted SNPs
−log10(p-value)
Recombination rate (cM/Mb)
r2
0.8
0.6
0.4
0.2
rs57866767
LGI1→
SLC35G1→
←PIPSL
←PLCE1−AS2
PLCE1→
←PLCE1−AS1
←NOC3L
TBC1D12→
HELLS→
CYP2C18→
CYP2C19→
Position on chr10 (Mb)
rs72837408
HABP2→
←NRAP
CASP7→
PLEKHS1→
←MIR4483
←DCLRE1A
NHLRC2→
ADRB1→
←MIR2110
←CCDC186
TDRD1→
VWA2→
rs10430737
←CHST15
←OAT
←NKX1−2
LHPP→
FAM53B−AS1→
←FAM53B
FAM175B→
←EEF1AKMT2
ZRANB1→
←CTBP2
←MIR4296
rs4141194
C11orf58→
←SNORD147
←PLEKHA7
OR7E14P→
←RPS13
←PIK3C2A
NUCB2→
←ABCC8
LOC105376575→
NCR3LG1→
←KCNJ11
Position on chr11 (Mb)
rs7111873
C11orf49→
←ARFGAP2
←PACSIN3
←MIR6745
DDB2→
←ACP2
NR1H3→
←LOC101928943
←MYBPC3
MADD→
←SPI1
MIR4487→
SLC39A13→
←PSMC3
←RAPSN
←CELF1
PTPMT1→
←KBTBD4
NDUFS3→
FAM180B→
←C1QTNF4
←MTCH2
AGBL2→
←FNBP4
←NUP160

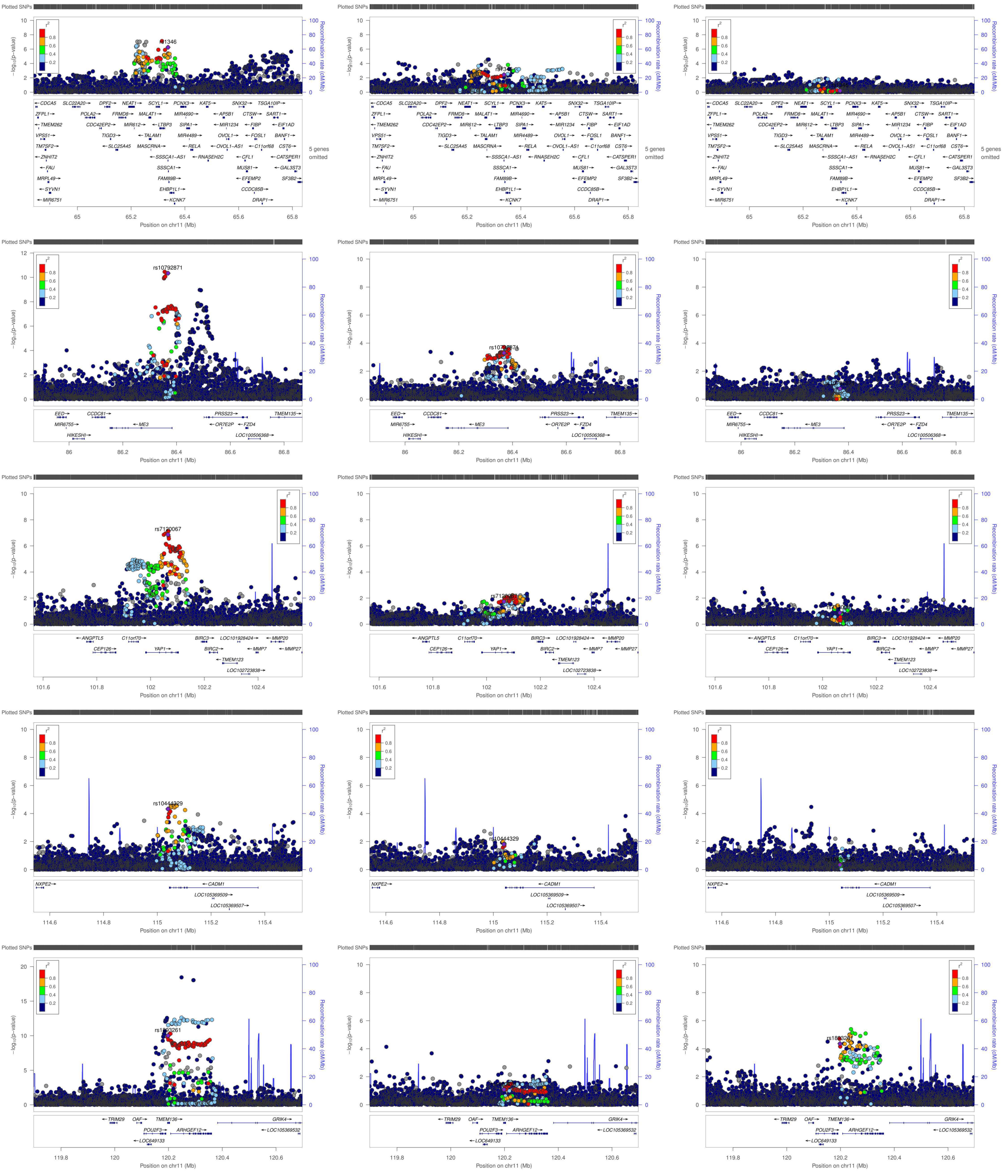
Plotted SNPs
−log10(p−value)
Recombination rate (cM/Mb)
r2
0.8
0.6
0.4
0.2
rs1346
CDCA5
SLC22A20
DPF2
NEAT1
SCYL1
PCNX3
KAT5
SNX32
TSGA10IP
ZFPL1
POLA2
FRMD8
MALAT1
MIR4690
AP5B1
CTSW
SART1
TMEM262
CDC42EP2
MIR612
LTBP3
SIPA1
MIR1234
FIBP
EIF1AD
VPS51
TIGD3
TALAM1
MIR4489
OVOL1
FOSL1
BANF1
TM7SF2
SLC25A45
MASCRNA
RELA
OVOL1−AS1
C11orf68
CST6
ZNHIT2
SSSCA1−AS1
RNASEH2C
CFL1
CATSPER1
FAU
SSSCA1
MUS81
GAL3ST3
MRPL49
FAM89B
EFEMP2
SF3B2
SYVN1
EHBP1L1
CCDC85B
MIR6751
KCNK7
DRAP1
5 genes omitted
Position on chr11 (Mb)
rs10792871
EED
CCDC81
PRSS23
TMEM135
MIR6755
ME3
OR7E2P
FZD4
HIKESHI
LOC100506368
rs7120067
ANGPTL5
C11orf70
BIRC3
LOC101928424
MMP20
CEP126
YAP1
BIRC2
MMP7
MMP27
TMEM123
LOC102723838
rs10444329
NXPE2
CADM1
LOC105369509
LOC105369507
rs1893261
TRIM29
OAF
TMEM136
GRIK4
POU2F3
ARHGEF12
LOC105369532
LOC649133

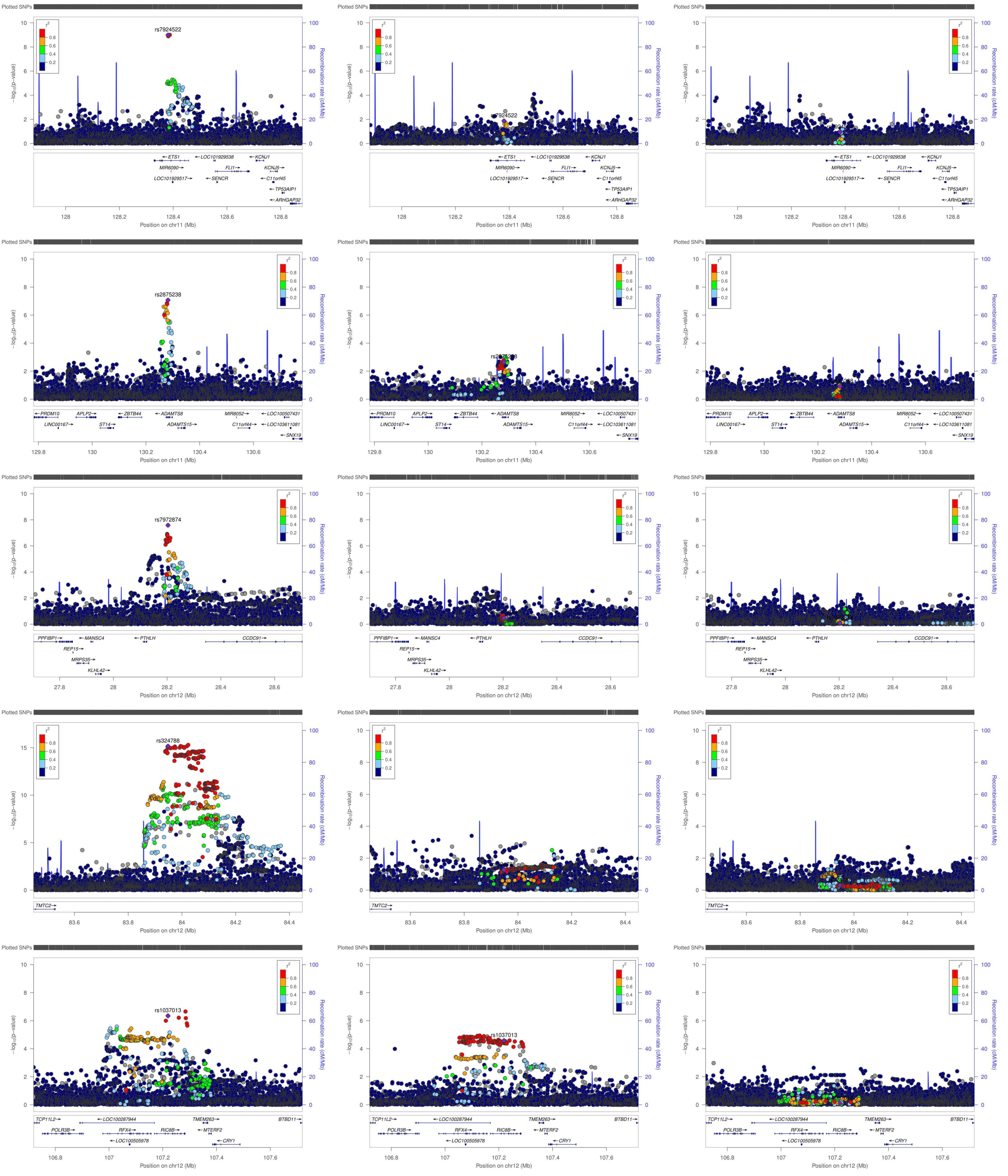

Plotted SNPs
rs7924522
ETS1
LOC101929538
KCNJ1
MIR6090
FLI1
KCNJ5
LOC101929517
SENCR
C11orf45
TP53AIP1
ARHGAP32
Position on chr11 (Mb)
rs2875238
PRDM10
APLP2
ZBTB44
ADAMTS8
MIR8052
LOC100507431
LINC00167
ST14
ADAMTS15
C11orf44
LOC103611081
SNX19
rs7972874
PPFIBP1
MANSC4
PTHLH
CCDC91
REP15
MRPS35
KLHL42
Position on chr12 (Mb)
rs324788
TMTC2
rs1037013
TCP11L2
LOC100287944
TMEM263
BTBD11
POLR3B
RFX4
RIC8B
MTERF2
LOC100505978
CRY1
−log10(p-value)
Recombination rate (cM/Mb)
r2

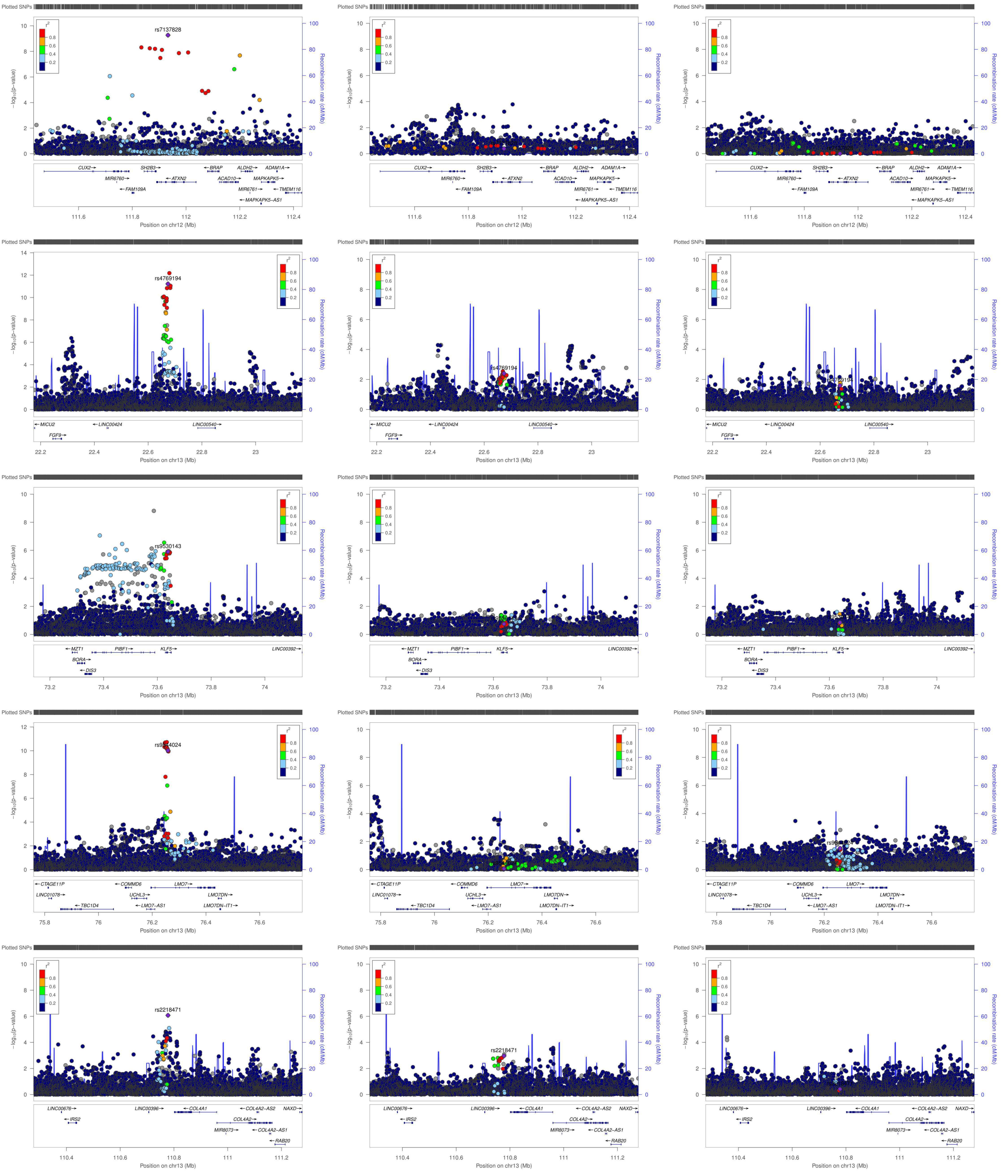
Plotted SNPs
−log10(p−value)
Recombination rate (cM/Mb)
r2
0.8
0.6
0.4
0.2
rs7137828
CUX2→
SH2B3→
←BRAP
ALDH2→
ADAM1A→
MIR6760→
←ATXN2
ACAD10→
MAPKAPK5→
←FAM109A
MIR6761→
←TMEM116
←MAPKAPK5−AS1
Position on chr12 (Mb)
rs4769194
←MICU2
←LINC00424
LINC00540→
FGF9→
Position on chr13 (Mb)
rs9530143
←MZT1
PIBF1→
KLF5→
LINC00392→
BORA→
←DIS3
rs9594024
←CTAGE11P
←COMMD6
LMO7→
LINC01078→
UCHL3→
LMO7DN→
←TBC1D4
←LMO7−AS1
LMO7DN−IT1→
rs2218471
LINC00676→
LINC00396→
←COL4A1
←COL4A2−AS2
NAXD→
←IRS2
COL4A2→
MIR8073→
←COL4A2−AS1
←RAB20

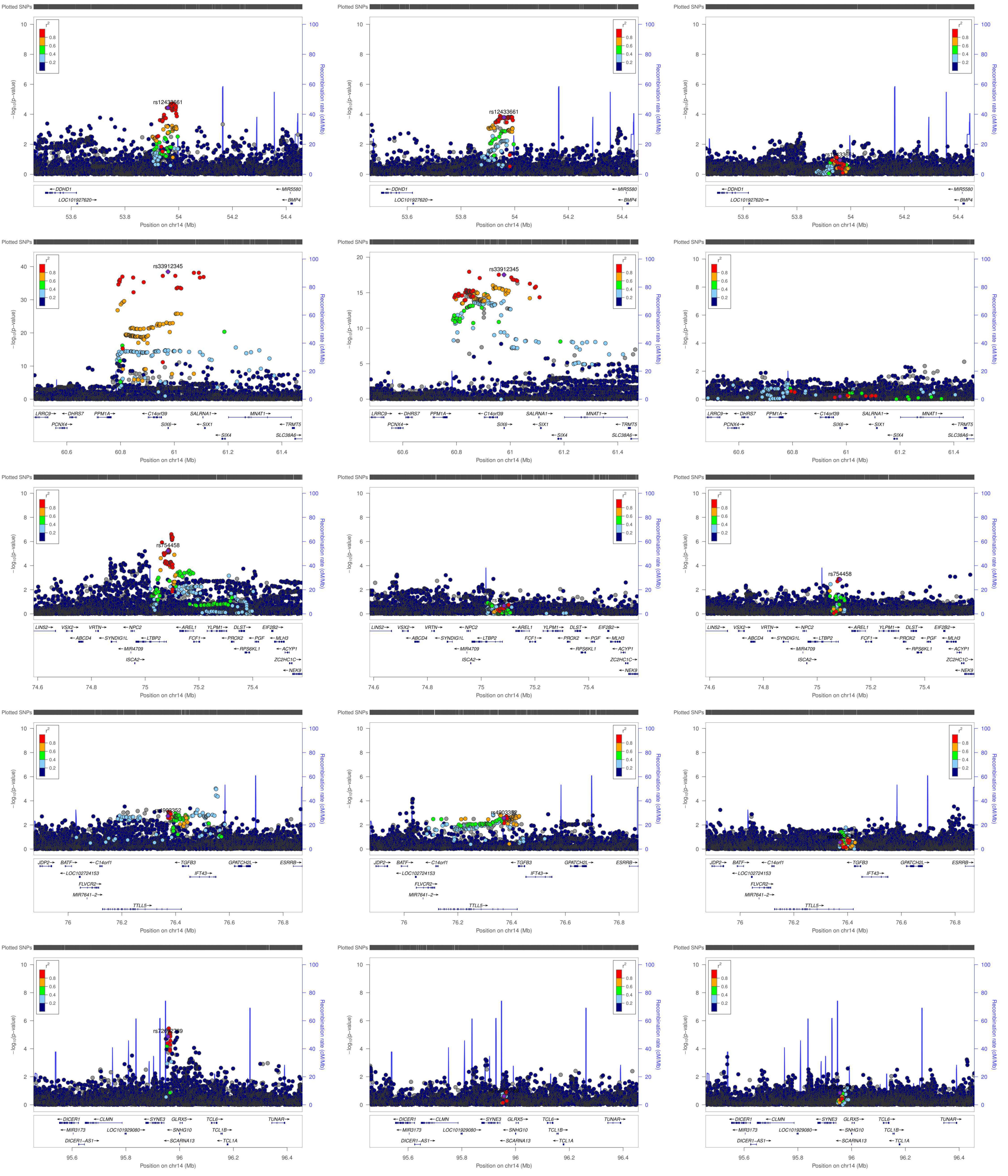
Plotted SNPs
r2
0.8
0.6
0.4
0.2
−log10(p−value)
Recombination rate (cM/Mb)
rs12433661
DDHD1
LOC101927620
MIR5580
BMP4
Position on chr14 (Mb)
rs33912345
LRRC9
DHRS7
PCNX4
PPM1A
C14orf39
SIX6
SALRNA1
SIX1
SIX4
MNAT1
TRMT5
SLC38A6
rs754458
LIN52
VSX2
VRTN
NPC2
ABCD4
SYNDIG1L
LTBP2
MIR4709
ISCA2
AREL1
FCF1
YLPM1
PROX2
DLST
RPS6KL1
PGF
EIF2B2
MLH3
ACYP1
ZC2HC1C
NEK9
rs4903352
JDP2
BATF
C14orf1
LOC102724153
FLVCR2
MIR7641−2
TTLL5
TGFB3
IFT43
GPATCH2L
ESRRB
rs72692739
DICER1
CLMN
MIR3173
DICER1−AS1
LOC101929080
SYNE3
GLRX5
SNHG10
SCARNA13
TCL6
TCL1B
TCL1A
TUNAR

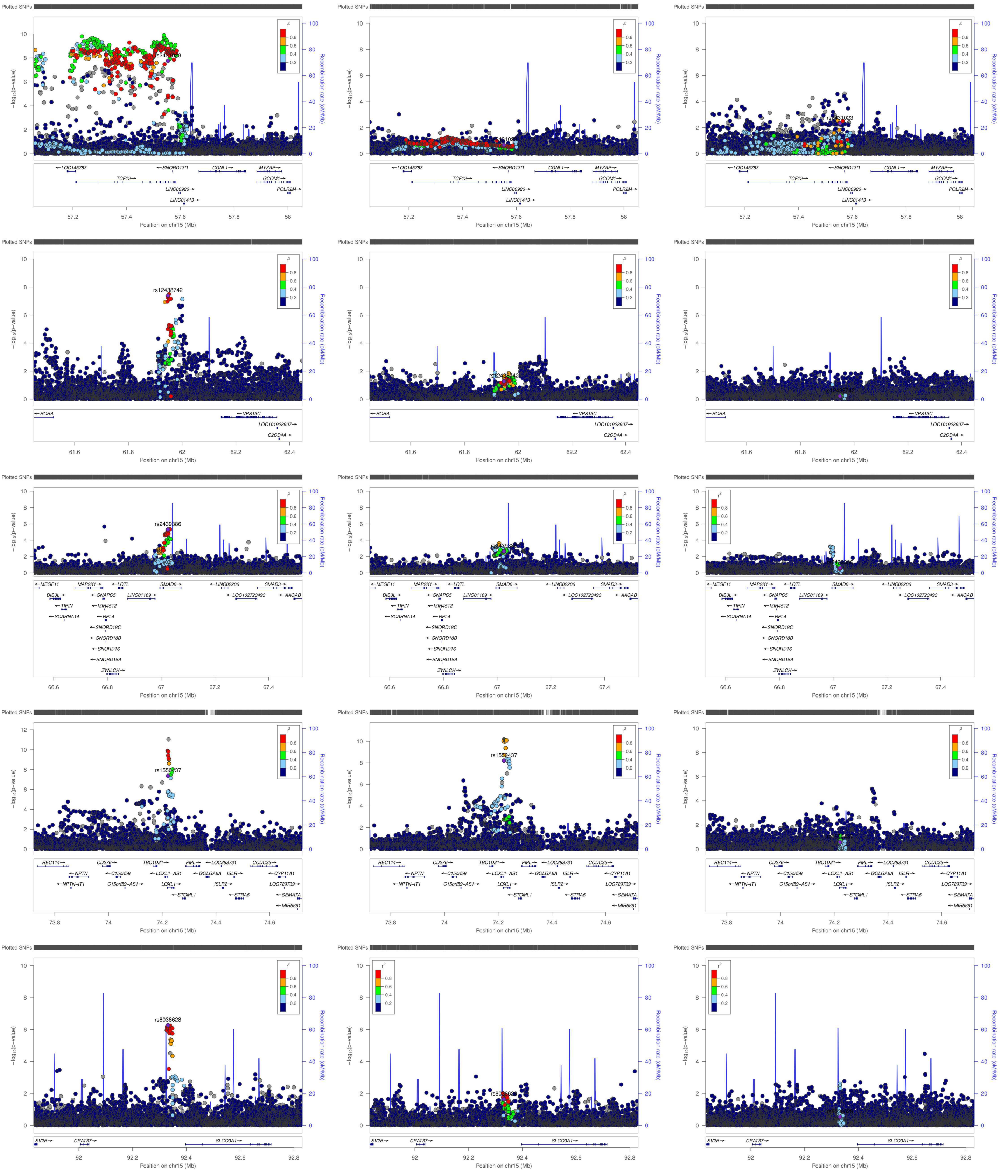

Plotted SNPs
−log10(p−value)
Recombination rate (cM/Mb)
r2
0.8
0.6
0.4
0.2
rs2431023
LOC145783
SNORD13D
CGNL1
MYZAP
TCF12
GCOM1
LINC00926
POLR2M
LINC01413
Position on chr15 (Mb)
rs12438742
RORA
VPS13C
LOC101928907
C2CD4A
rs2439386
MEGF11
MAP2K1
LCTL
SMAD6
LINC02206
SMAD3
DIS3L
SNAPC5
LINC01169
LOC102723493
AAGAB
TIPIN
MIR4512
SCARNA14
RPL4
SNORD18C
SNORD18B
SNORD16
SNORD18A
ZWILCH
rs1550437
REC114
CD276
TBC1D21
PML
LOC283731
CCDC33
NPTN
C15orf59
LOXL1−AS1
GOLGA6A
ISLR
CYP11A1
NPTN−IT1
C15orf59−AS1
LOXL1
ISLR2
LOC729739
STOML1
STRA6
SEMA7A
MIR6881
rs8038628
SV2B
CRAT37
SLCO3A1

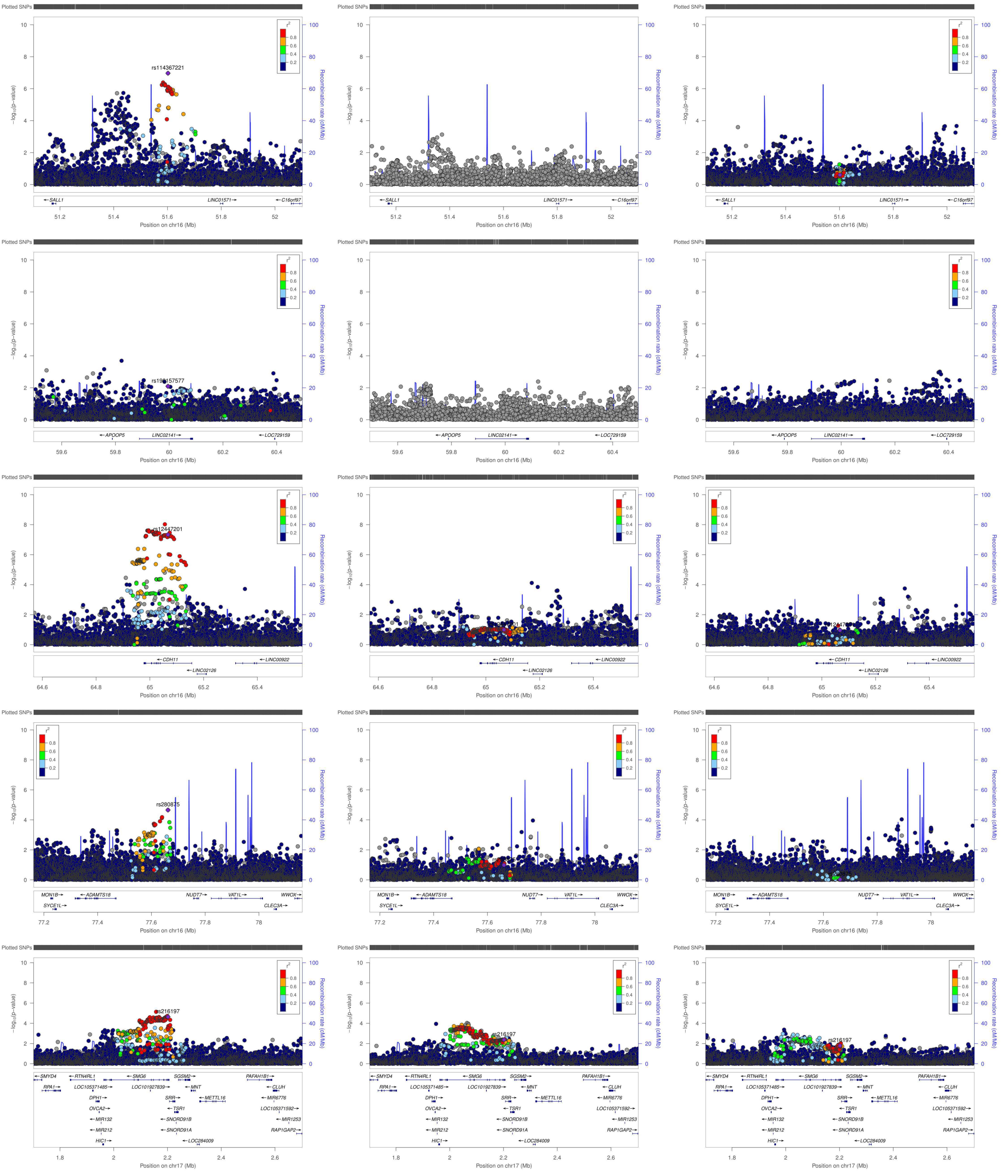

Plotted SNPs
−log10(p−value)
Recombination rate (cM/Mb)
r2
0.8
0.6
0.4
0.2
rs114367221
SALL1
LINC01571
C16orf97
Position on chr16 (Mb)
rs199157577
APOOP5
LINC02141
LOC729159
rs12447201
CDH11
LINC00922
LINC02126
rs280875
MON1B
SYCE1L
ADAMTS18
NUDT7
VAT1L
WWOX
CLEC3A
rs216197
SMYD4
RPA1
RTN4RL1
LOC105371485
DPH1
OVCA2
MIR132
MIR212
HIC1
SMG6
LOC101927839
SRR
TSR1
SNORD91B
SNORD91A
SGSM2
MNT
METTL16
LOC284009
PAFAH1B1
CLUH
MIR6776
LOC105371592
MIR1253
RAP1GAP2
Position on chr17 (Mb)

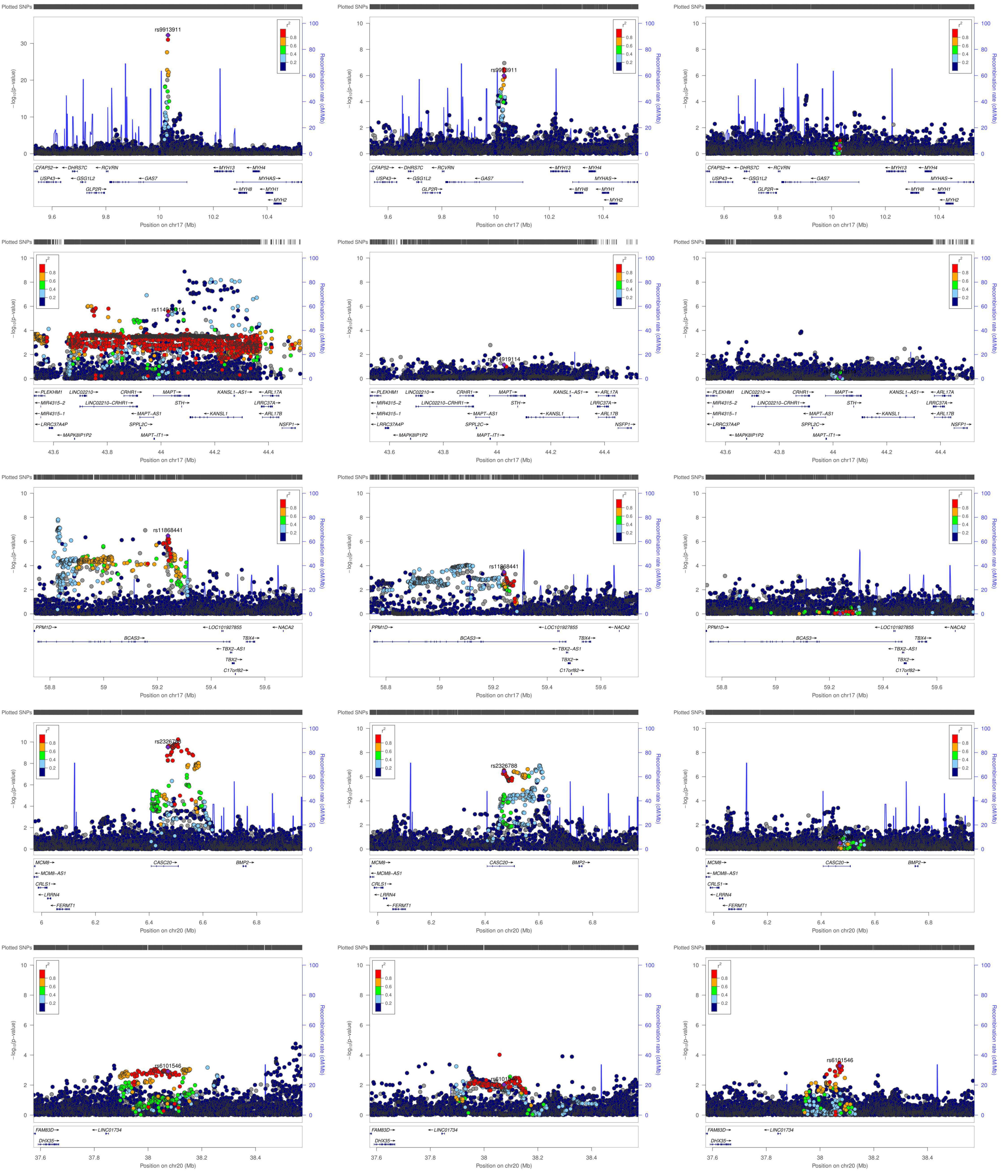
Plotted SNPs
rs9913911
−log10(p−value)
Recombination rate (cM/Mb)
r2
0.8
0.6
0.4
0.2
CFAP52
DHRS7C
RCVRN
MYH13
MYH4
USP43
GSG1L2
GAS7
MYHAS
GLP2R
MYH8
MYH1
MYH2
Position on chr17 (Mb)
rs11491914
PLEKHM1
LINC02210
CRHR1
MAPT
KANSL1−AS1
ARL17A
MIR4315−2
LINC02210−CRHR1
STH
LRRC37A
MIR4315−1
MAPT−AS1
KANSL1
ARL17B
LRRC37A4P
SPPL2C
NSFP1
MAPK8IP1P2
MAPT−IT1
rs11868441
PPM1D
LOC101927855
NACA2
BCAS3
TBX4
TBX2−AS1
TBX2
C17orf82
rs2326788
MCM8
CASC20
BMP2
MCM8−AS1
CRLS1
LRRN4
FERMT1
Position on chr20 (Mb)
rs6101546
FAM83D
LINC01734
DHX35

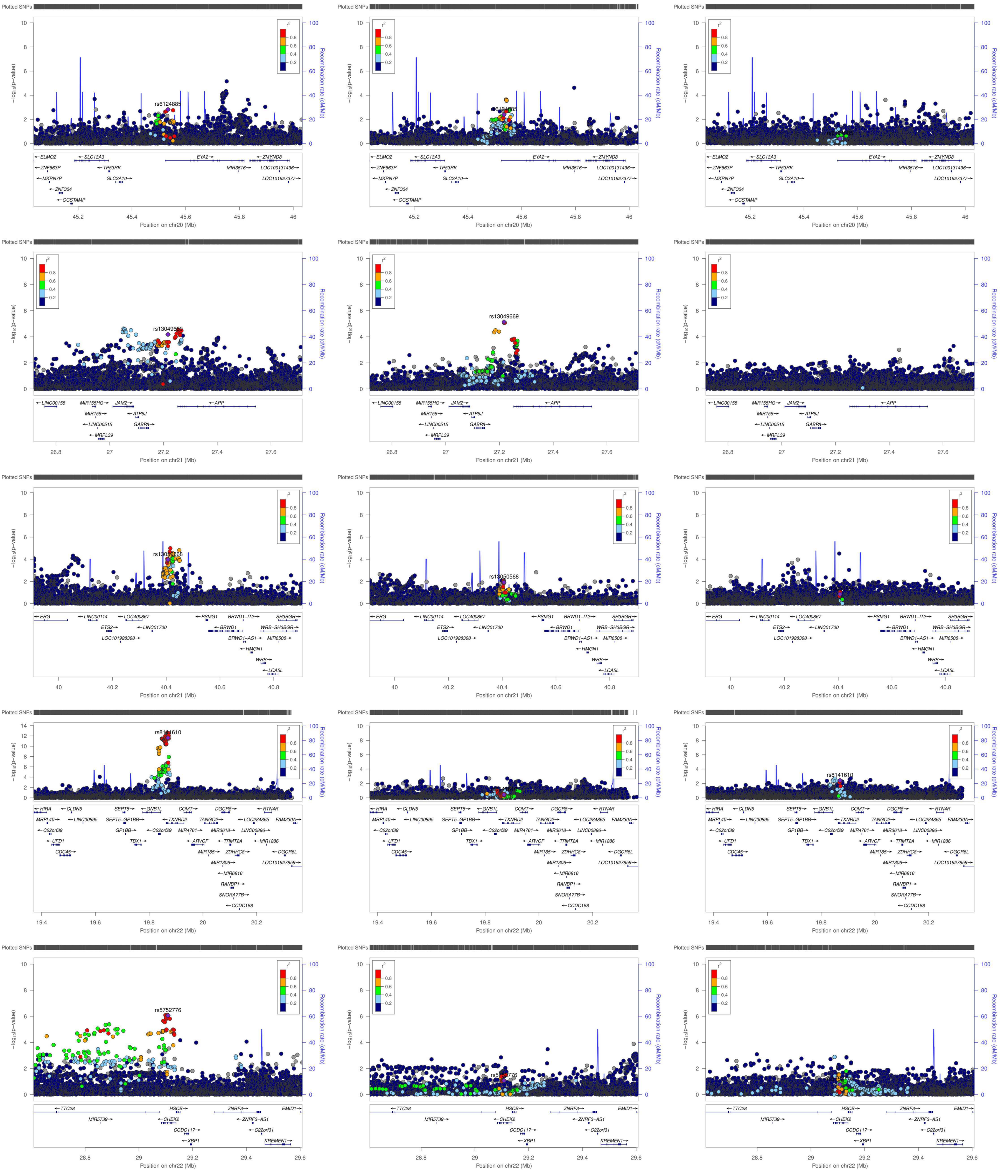

Plotted SNPs
-log10(p-value)
Recombination rate (cM/Mb)
r2
0.8
0.6
0.4
0.2
rs6124885
ELMO2
SLC13A3
EYA2
ZMYND8
ZNF663P
TP53RK
MIR3616
LOC100131496
MKRN7P
SLC2A10
LOC101927377
ZNF334
OCSTAMP
Position on chr20 (Mb)
rs13049669
LINC00158
MIR155HG
JAM2
APP
MIR155
ATP5J
LINC00515
GABPA
MRPL39
Position on chr21 (Mb)
rs13050568
ERG
LINC00114
LOC400867
PSMG1
BRWD1-IT2
SH3BGR
ETS2
LINC01700
BRWD1
WRB-SH3BGR
LOC101928398
BRWD1-AS1
MIR6508
HMGN1
WRB
LCA5L
rs8141610
HIRA
CLDN5
SEPT5
GNB1L
COMT
DGCR8
RTN4R
MRPL40
LINC00895
SEPT5-GP1BB
TXNRD2
TANGO2
LOC284865
FAM230A
C22orf39
GP1BB
C22orf29
MIR4761
MIR3618
LINC00896
UFD1
TBX1
ARVCF
TRMT2A
MIR1286
CDC45
MIR185
ZDHHC8
DGCR6L
MIR1306
LOC101927859
MIR6816
RANBP1
SNORA77B
CCDC188
Position on chr22 (Mb)
rs5752776
TTC28
HSCB
ZNRF3
EMID1
MIR5739
CHEK2
ZNRF3-AS1
CCDC117
C22orf31
XBP1
KREMEN1

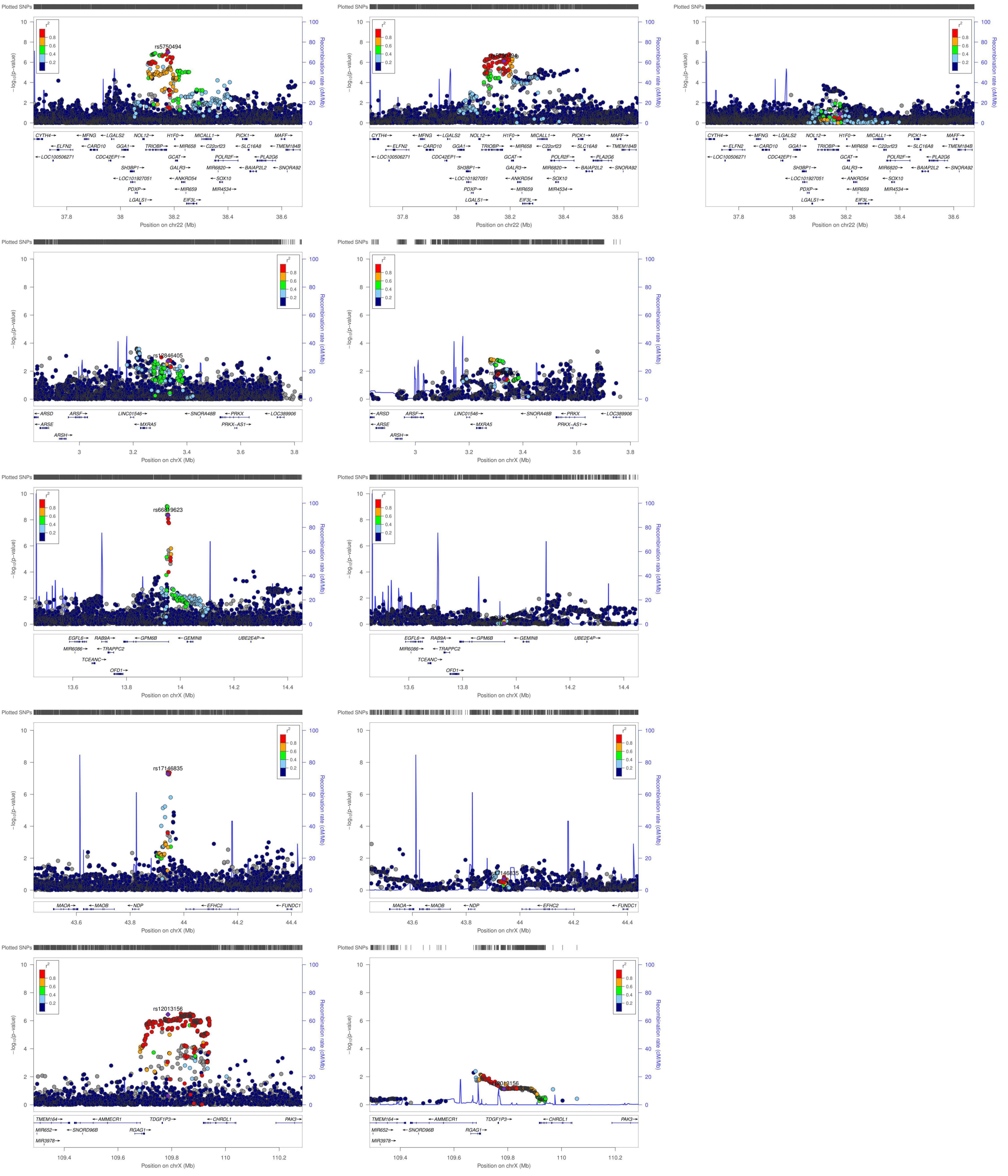

Plotted SNPs
−log10(p−value)
Recombination rate (cM/Mb)
r2
0.8
0.6
0.4
0.2
rs5750494
CYTH4→ ←MFNG ←LGALS2 NOL12→ H1F0→ MICALL1→ PICK1→ MAFF→
←ELFN2 ←CARD10 GGA1→ TRIOBP→ ←MIR658 ←C22orf23 ←SLC16A8 ←TMEM184B
←LOC100506271 CDC42EP1→ GCAT→ POLR2F→ ←PLA2G6
SH3BP1→ GALR3→ MIR6820→ ←BAIAP2L2 ←SNORA92
←LOC101927051 ←ANKRD54 ←SOX10
PDXP→ ←MIR659 MIR4534→
LGALS1→ EIF3L→
Position on chr22 (Mb)
rs12846405
←ARSD ARSF→ LINC01546→ ←SNORA48B ←PRKX ←LOC389906
←ARSE ←MXRA5 PRKX−AS1→
ARSH→
Position on chrX (Mb)
rs66819623
EGFL6→ RAB9A→ ←GPM6B ←GEMIN8 UBE2E4P→
MIR6086→ ←TRAPPC2
TCEANC→
OFD1→
rs17146835
MAOA→ ←MAOB ←NDP ←EFHC2 ←FUNDC1
rs12013156
TMEM164→ ←AMMECR1 TDGF1P3→ ←CHRDL1 PAK3→
MIR652→ ←SNORD96B RGAG1→
MIR3978→

Supplementary Figure 4. Heatmap of SNP effects on POAG, IOP, and VCDR. In this heatmap, SNPs have been clustered based on the Pearson correlation between their effect estimates on POAG, IOP, and VCDR. Three major clusters can be observed, separated by white space: 1) the largest cluster on the top, which includes POAG SNPs that affect IOP. 2) The bottom cluster, which includes POAG SNPs that affect VCDR. 3) The middle cluster, which includes POAG SNPs that do not appear to have a clear effect on IOP or VCDR. The magnitude of SNP effects are on Z-score scale, as shown by the color key on the right. To avoid the outlying effect of large values, Z scores > 10 were set to 10. All effect estimates refer to the risk-increasing alleles for POAG.

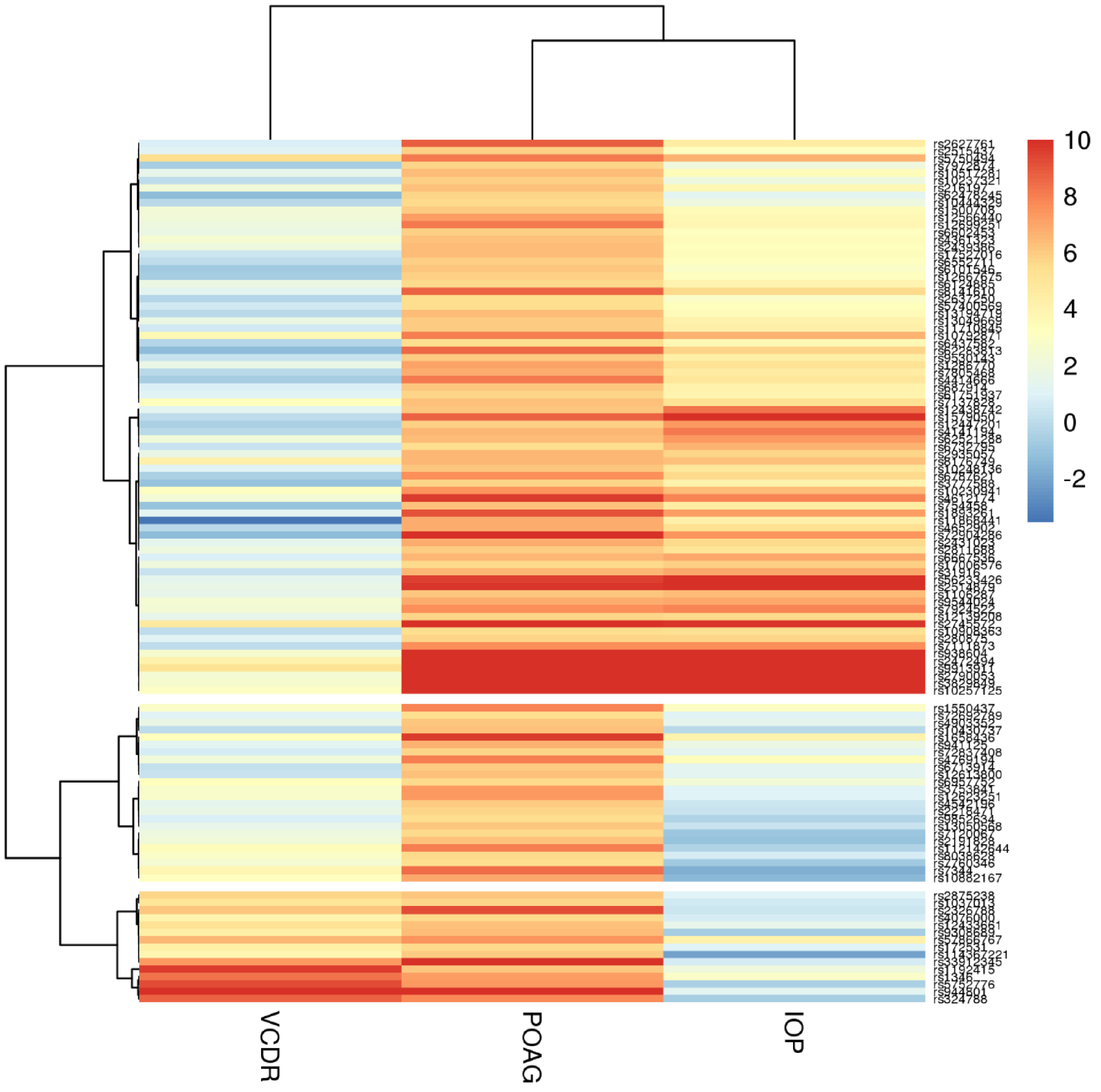

Supplementary Figure 5. Enrichment of the expression of the significant genes identified by MAGMA in GTEX tissues. The horizontal line indicates the significance threshold (P<1e-03), Bonferroni-corrected for 53 GTEX tissues tested.

a) Results from the European meta-analysis.

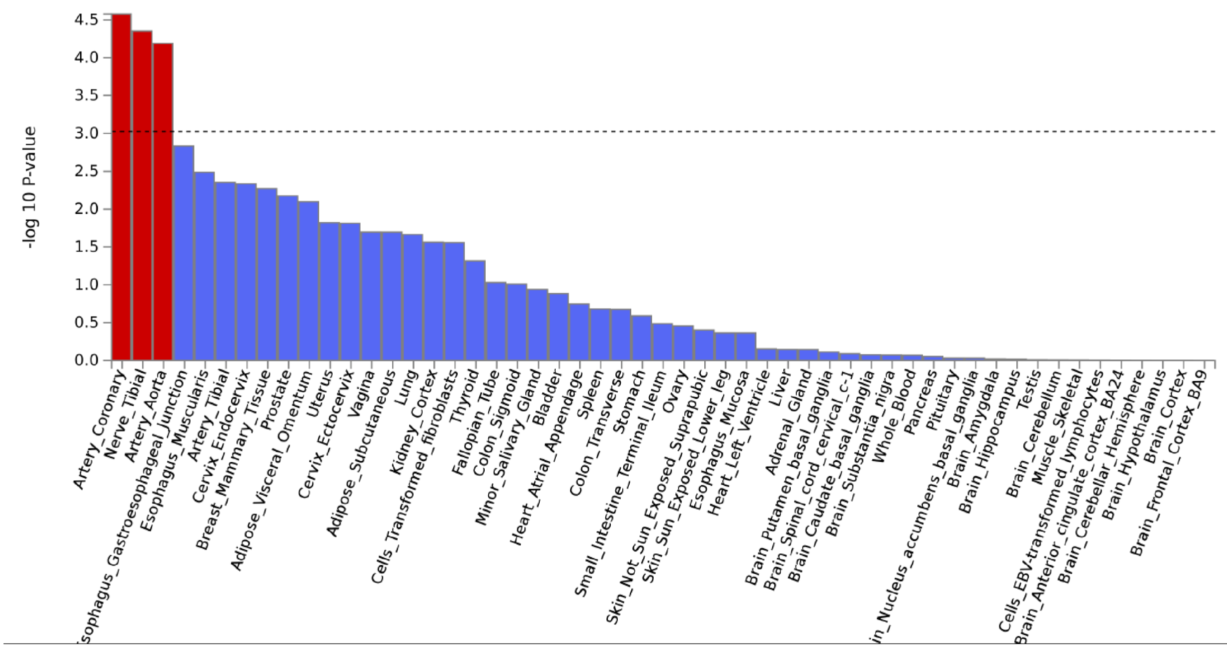

b) Results from the cross-ancestry meta-analysis.

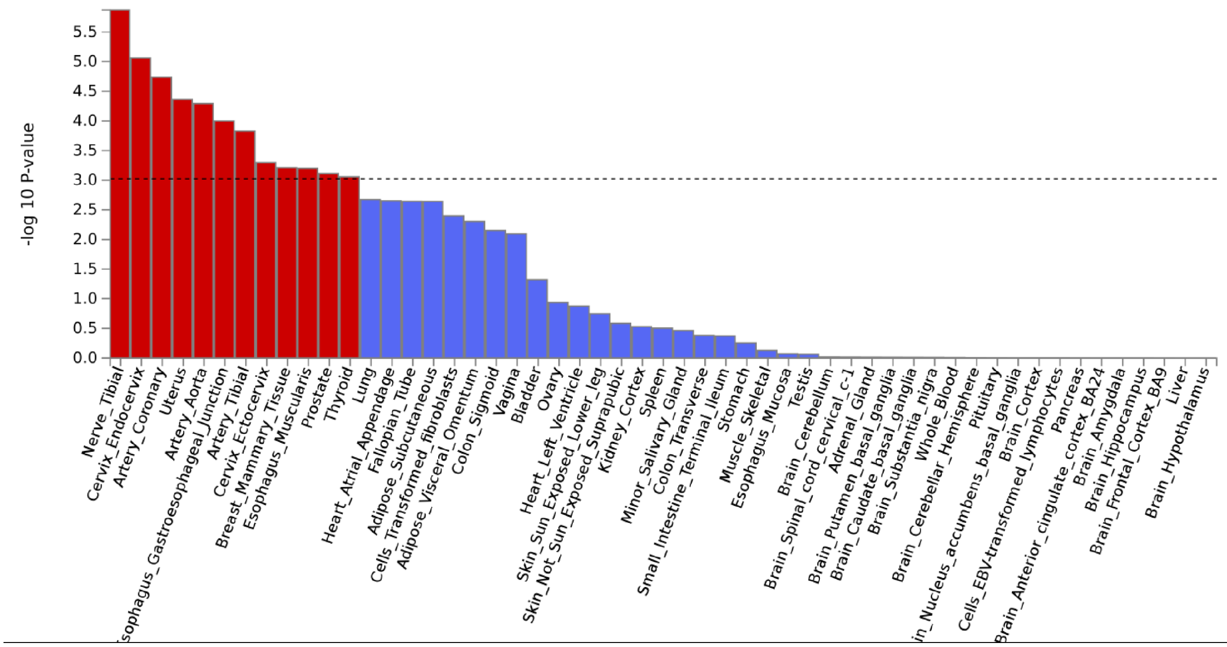

Supplementary Figure 6. Chromatin and eQTL interactions for the POAG risk loci. The interactions are shown for each chromosome separately. Orange lines show chromatin interactions and green lines eQTL interactions. Green genes are the eQTL-mapped genes, orange genes are mapped by chromatin interactions, and red genes by both. The outer layers are Manhattan plots with the most significant SNPs shown.

Chromosome 1

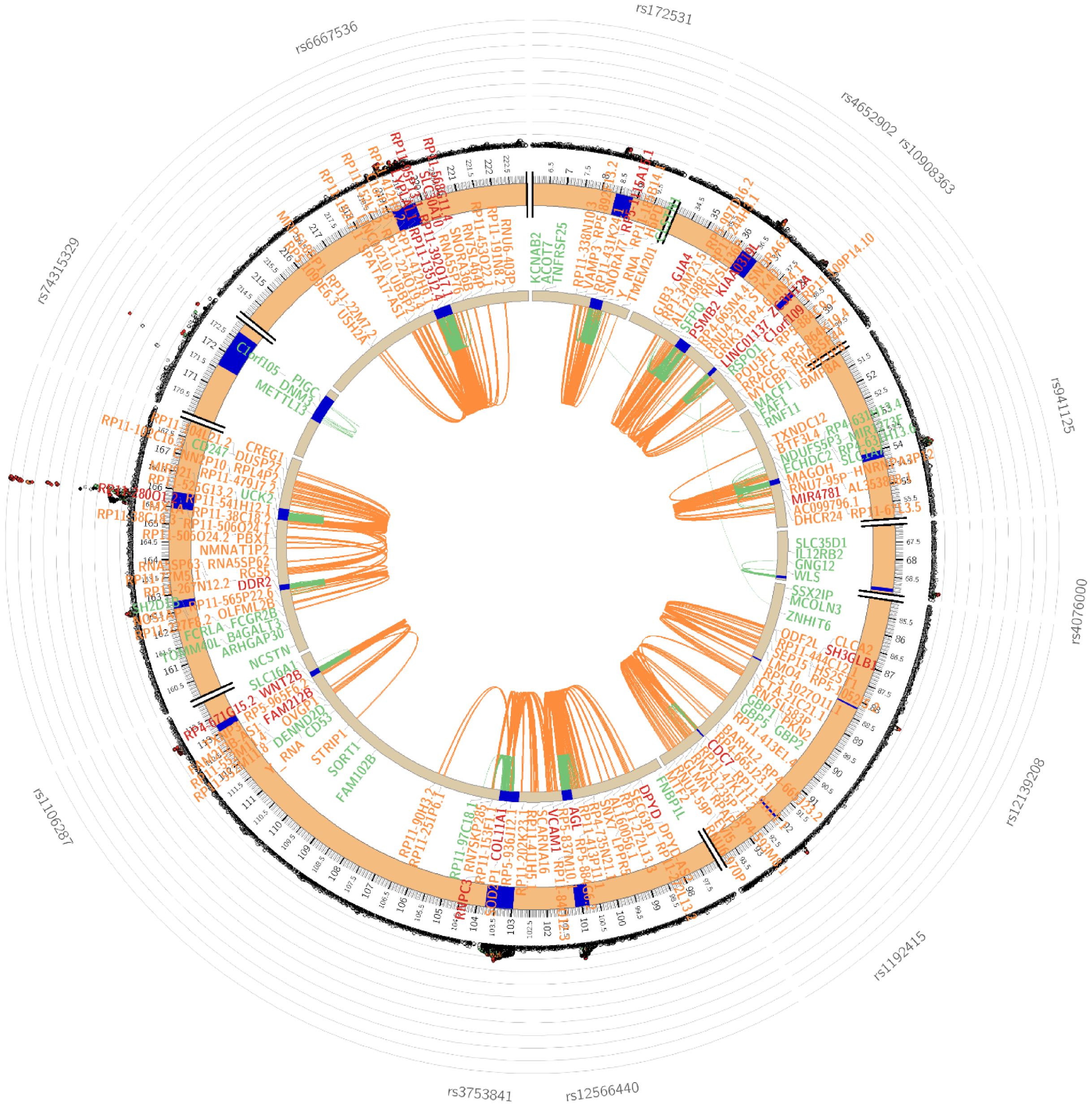

Chromosome 2

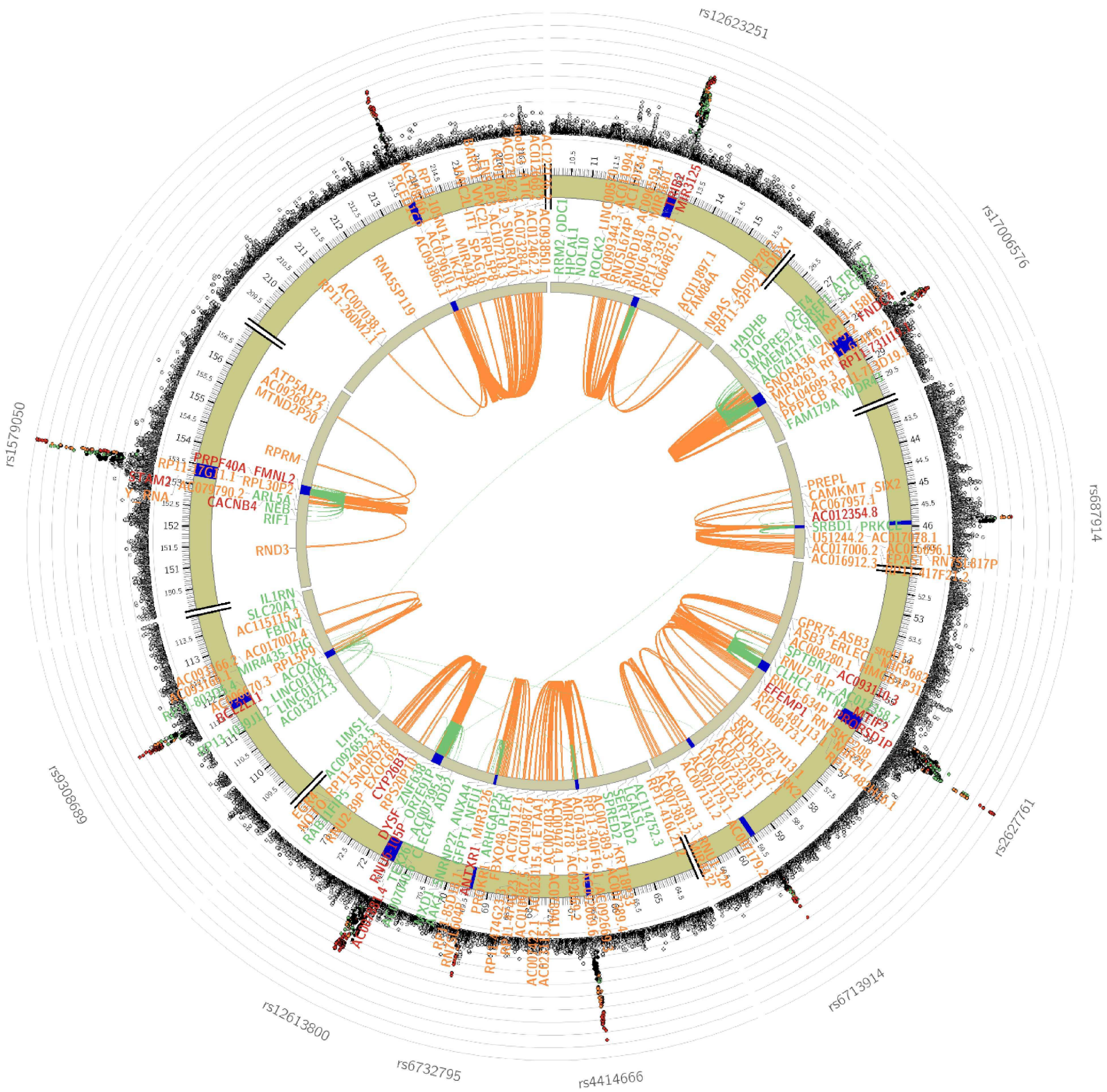

Chromosome 3

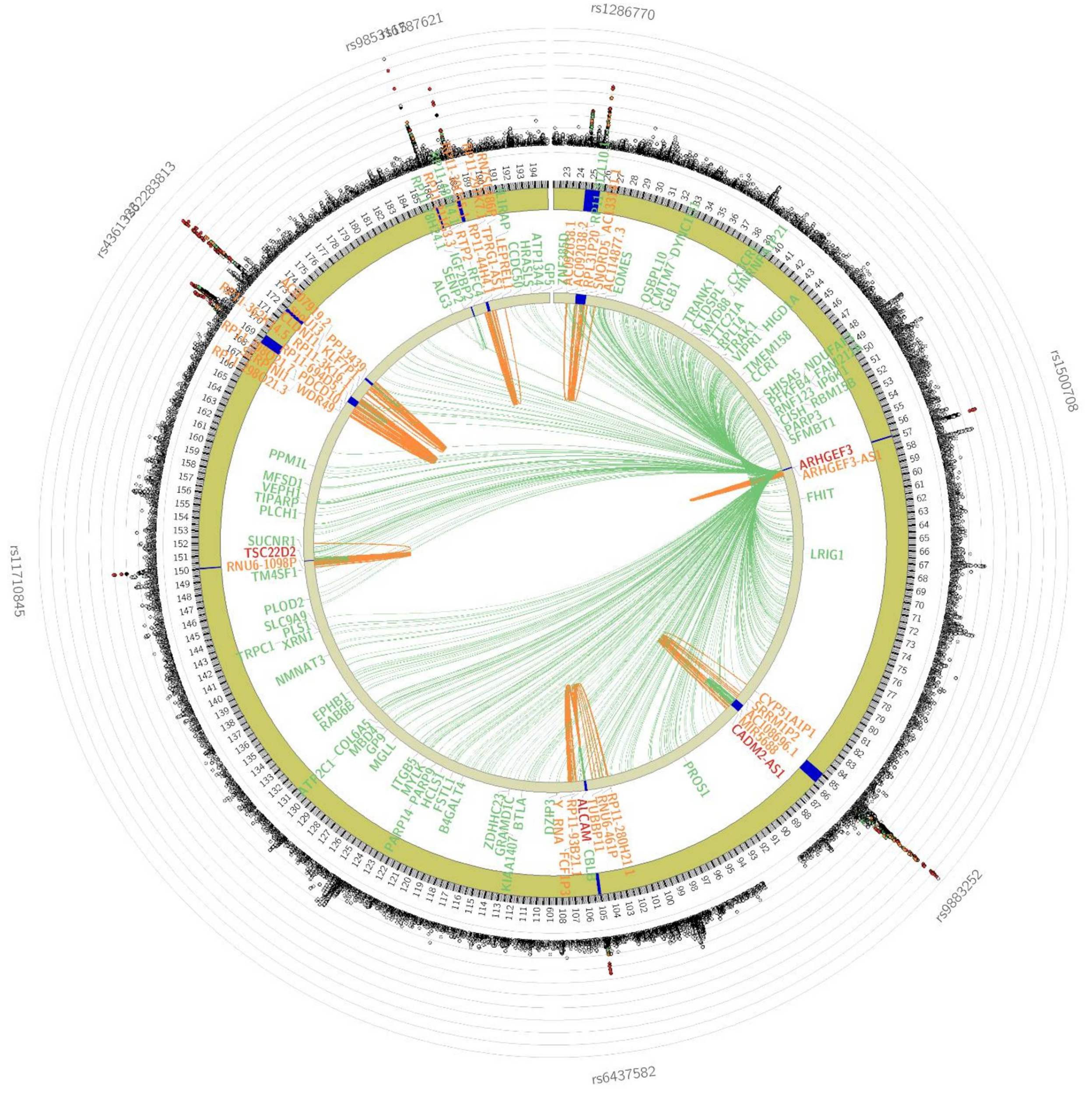

Chromosome 4

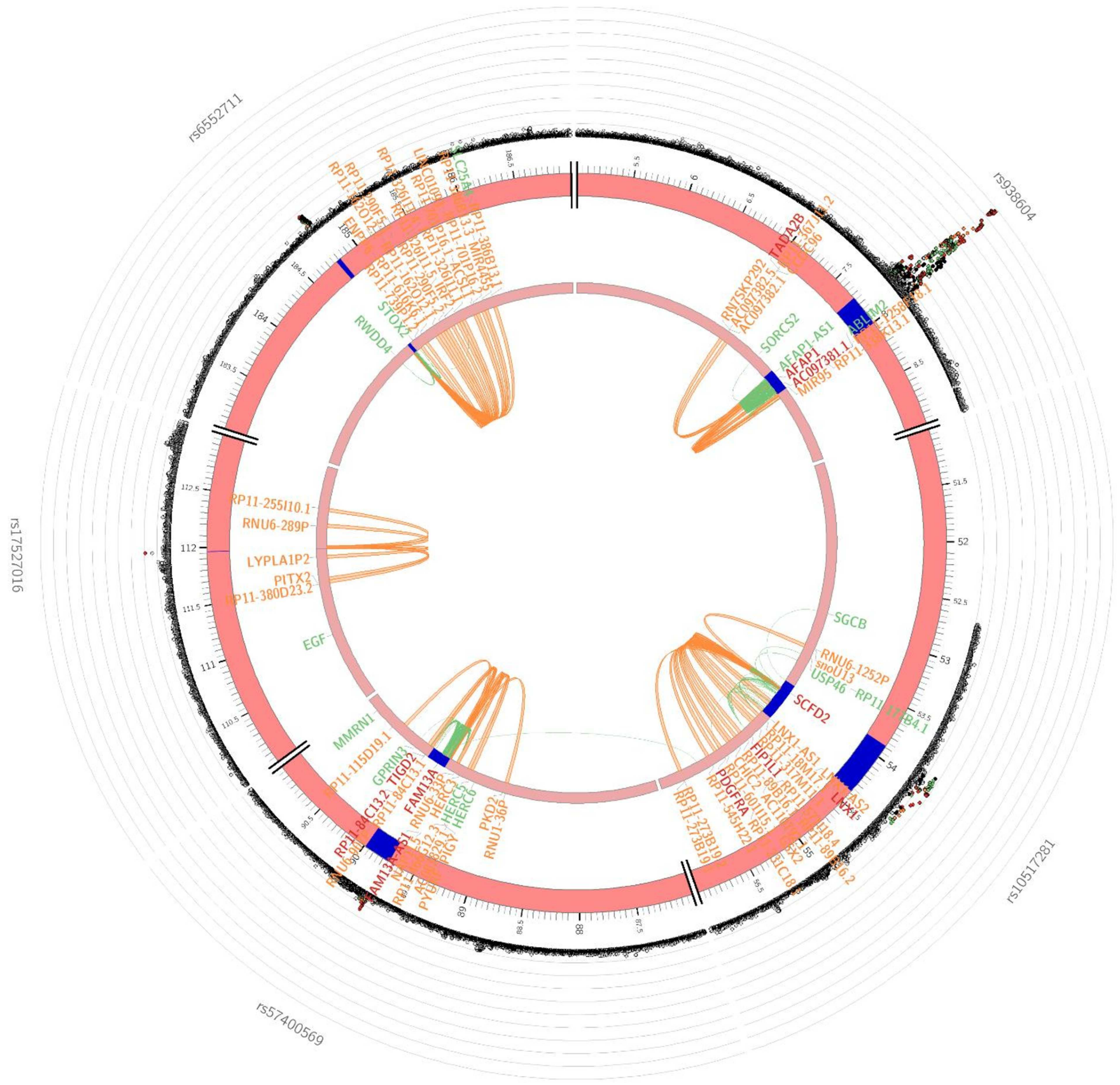

Chromosome 5

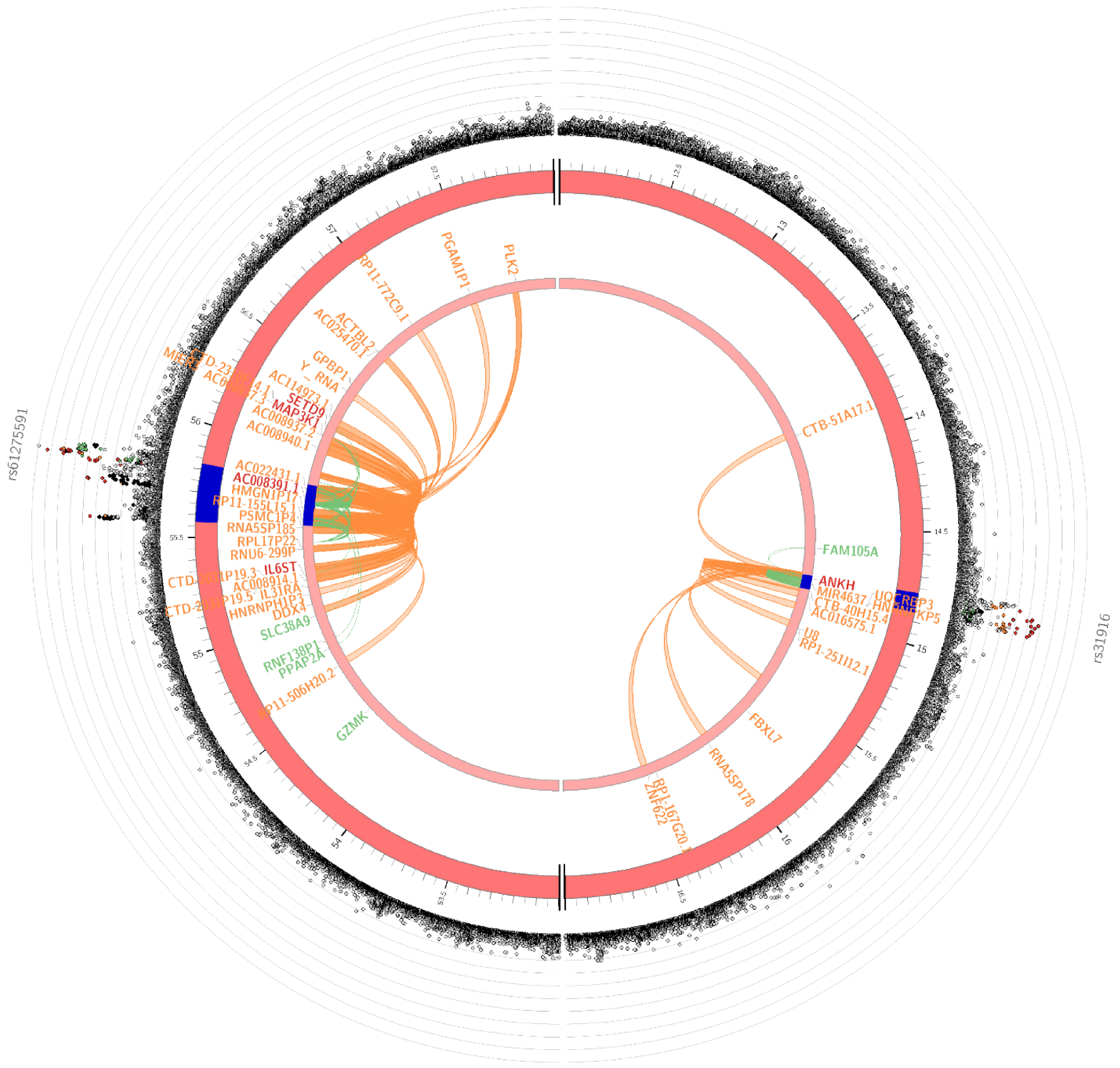

Chromosome 6

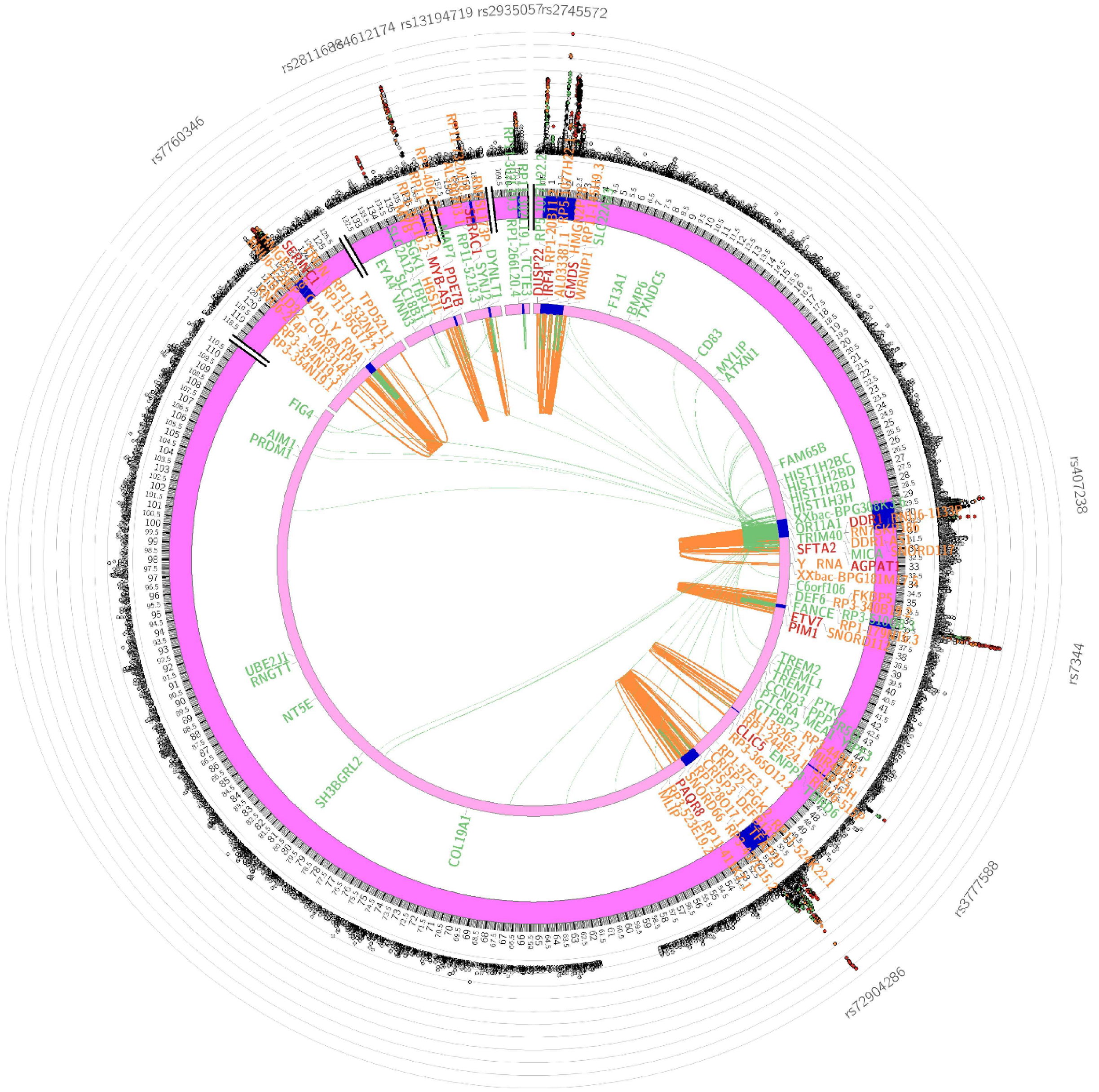

Chromosome 7

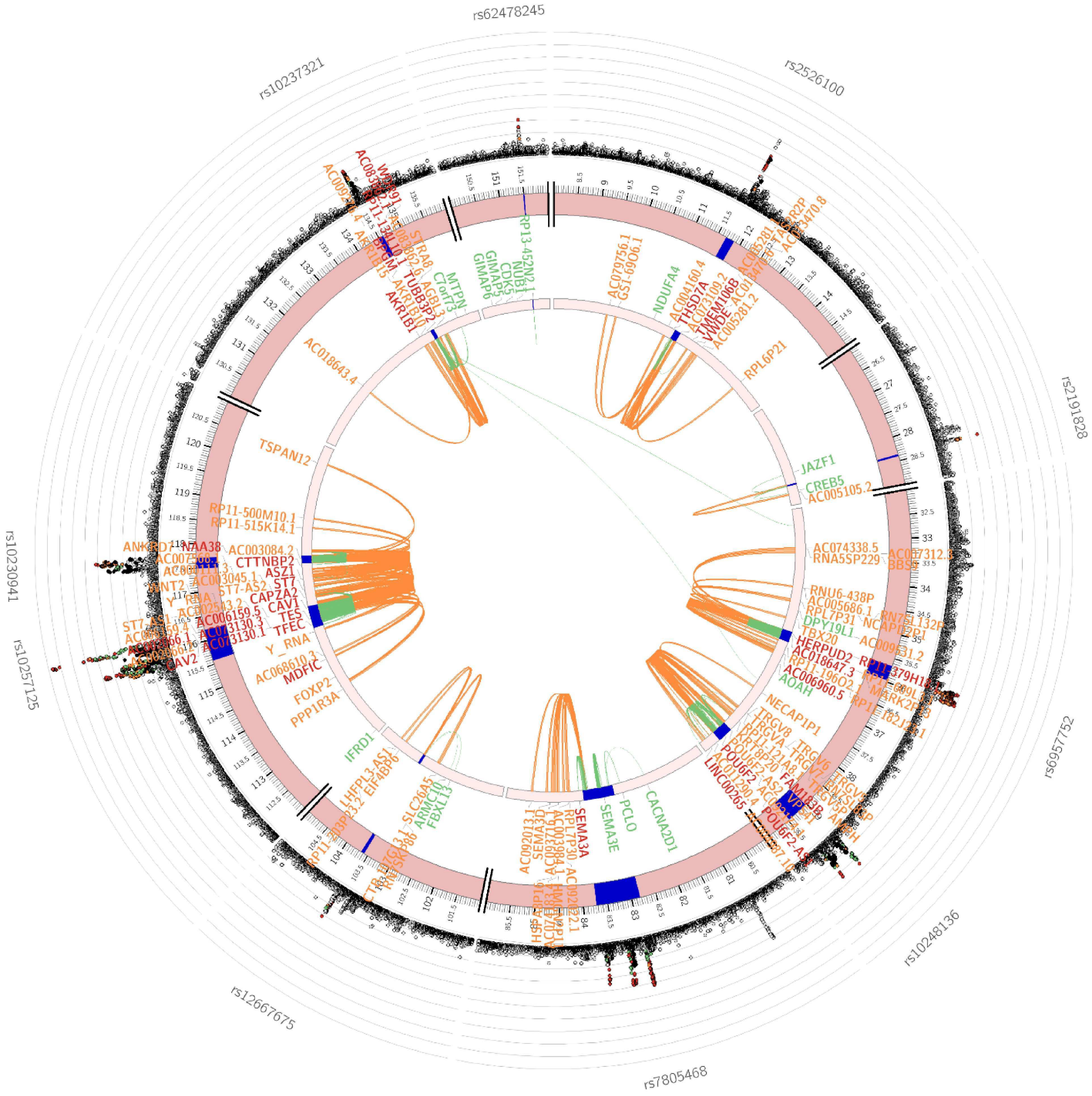

Chromosome 8

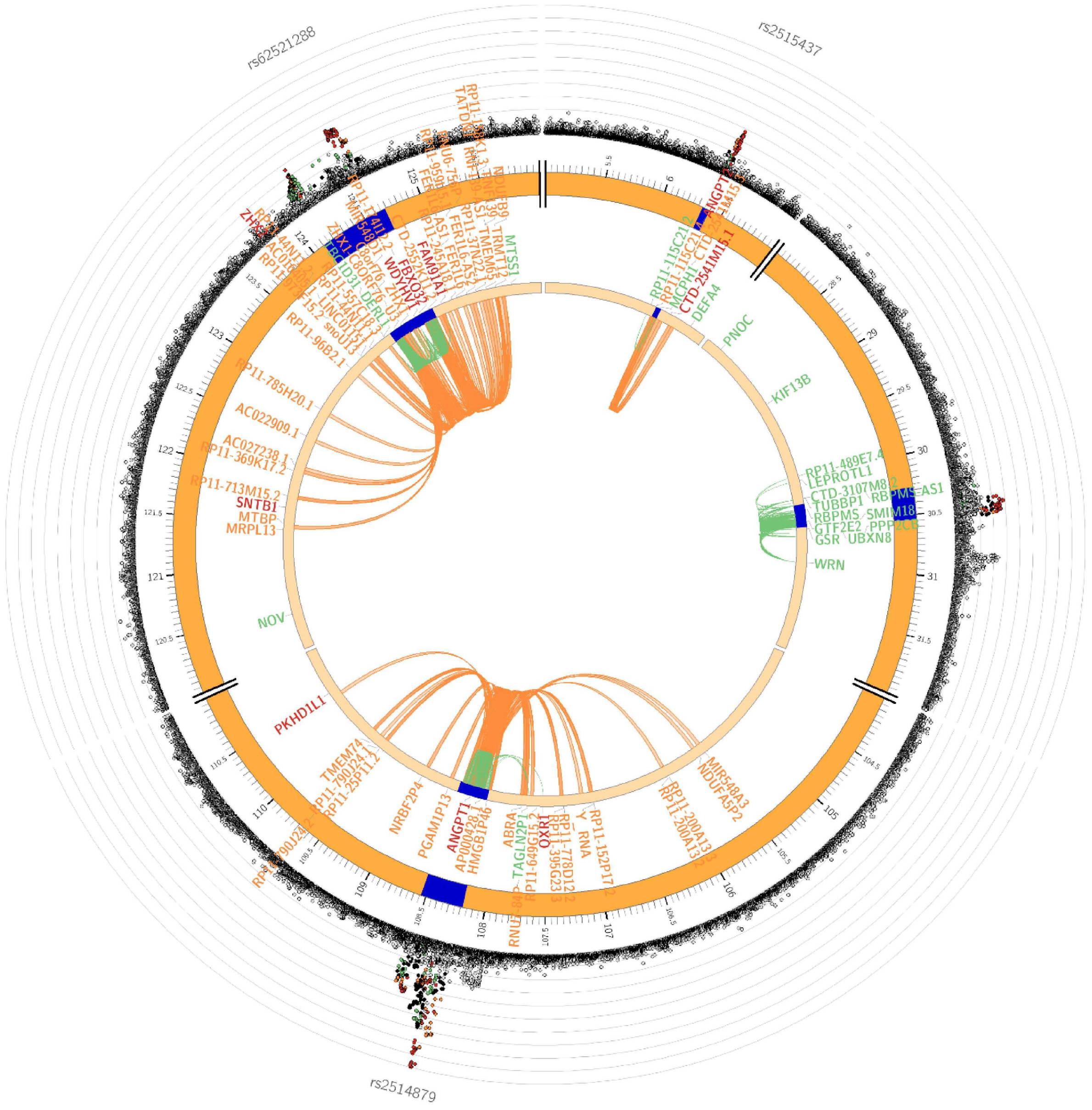

Chromosome 9

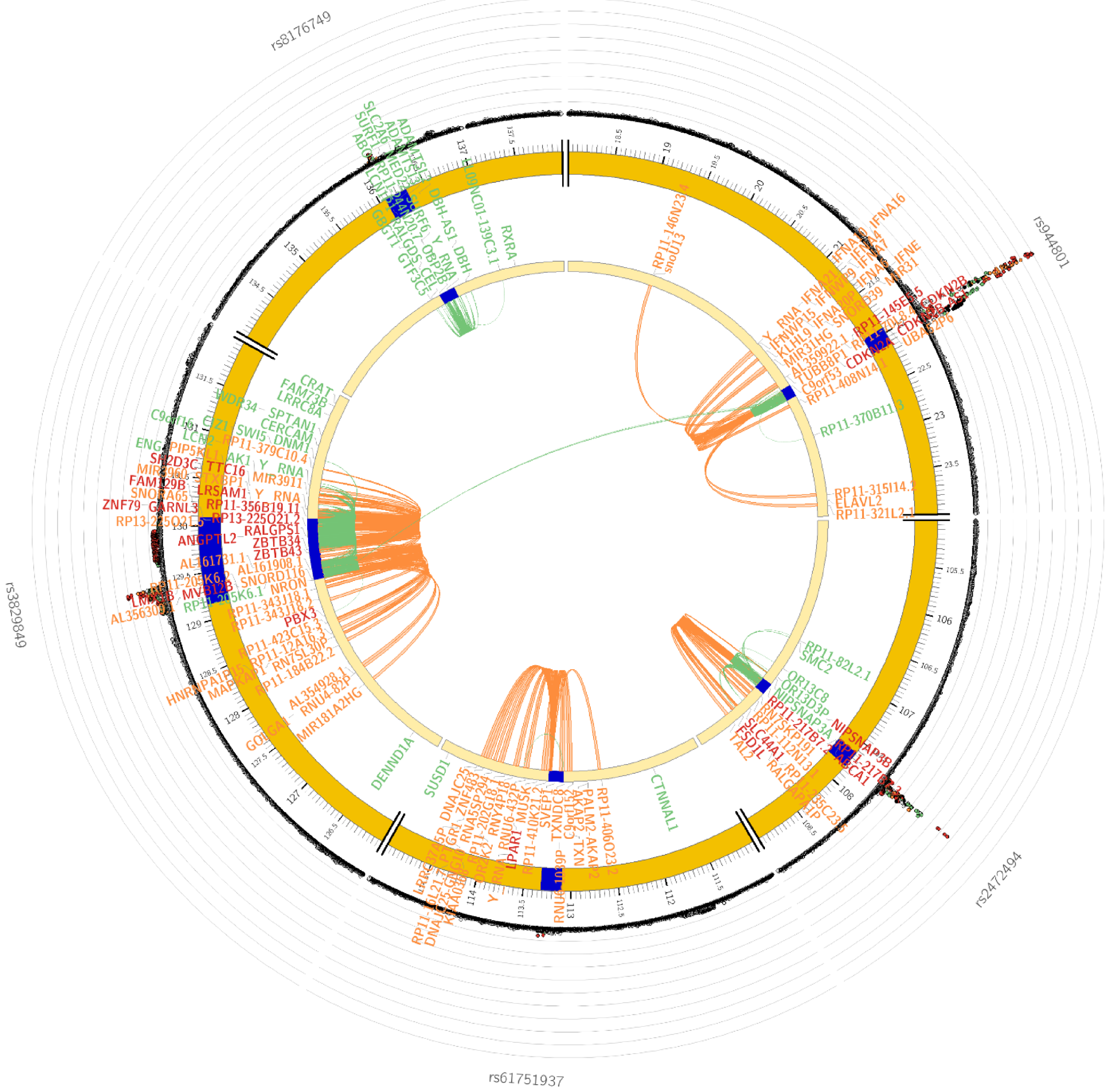

Chromosome 10

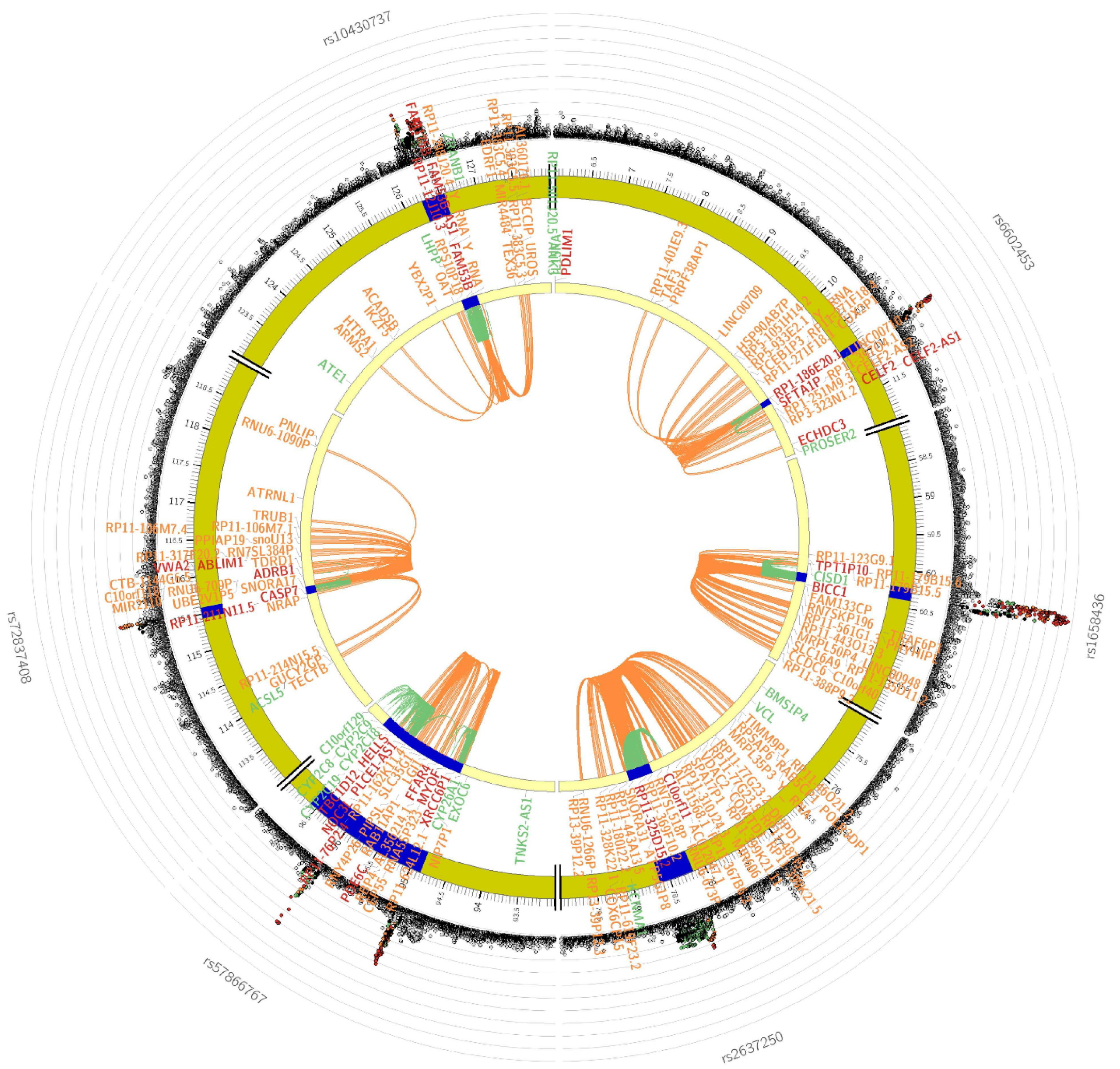

Chromosome 11

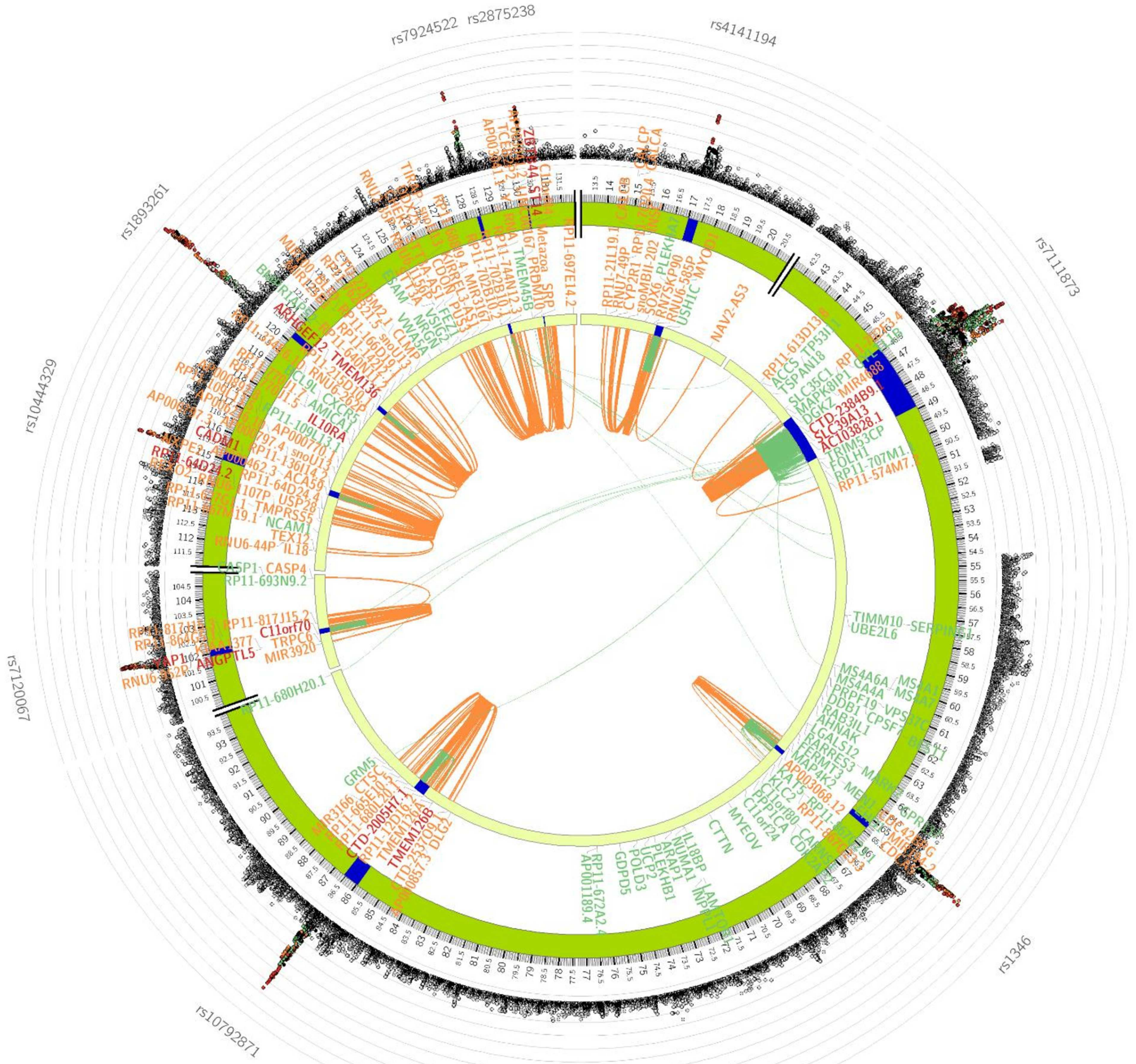

Chromosome 12

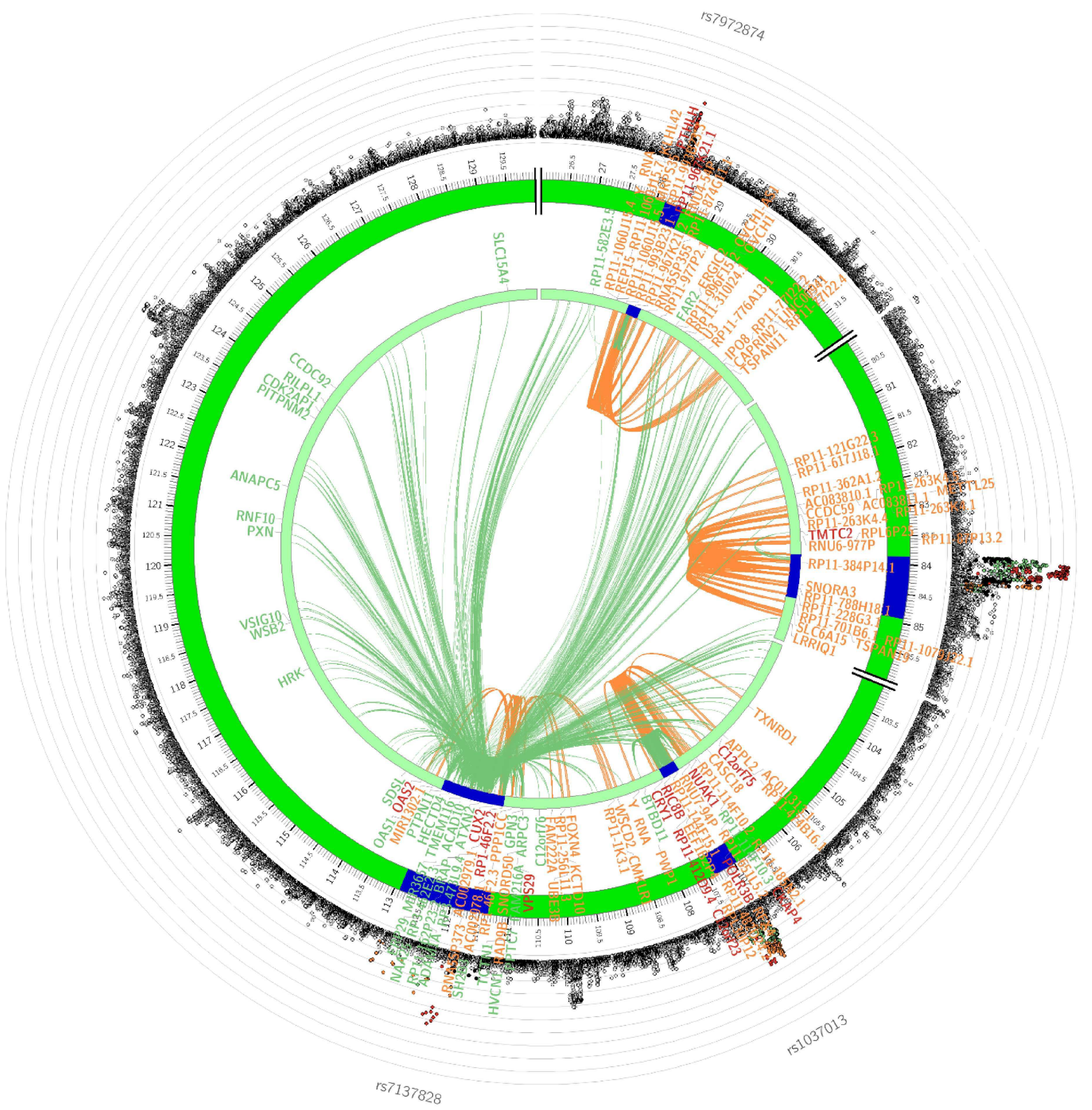

Chromosome 13

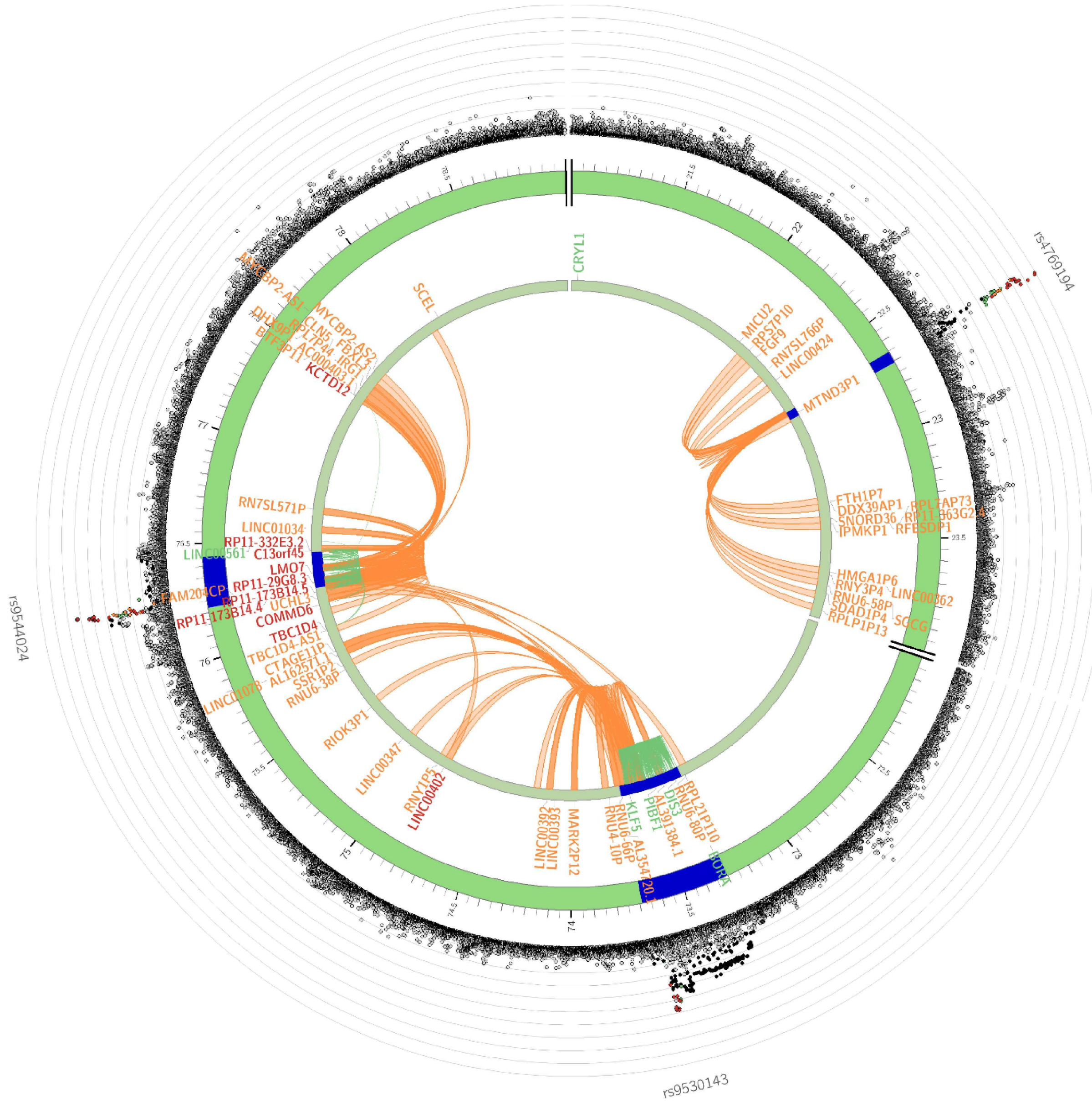

Chromosome 14

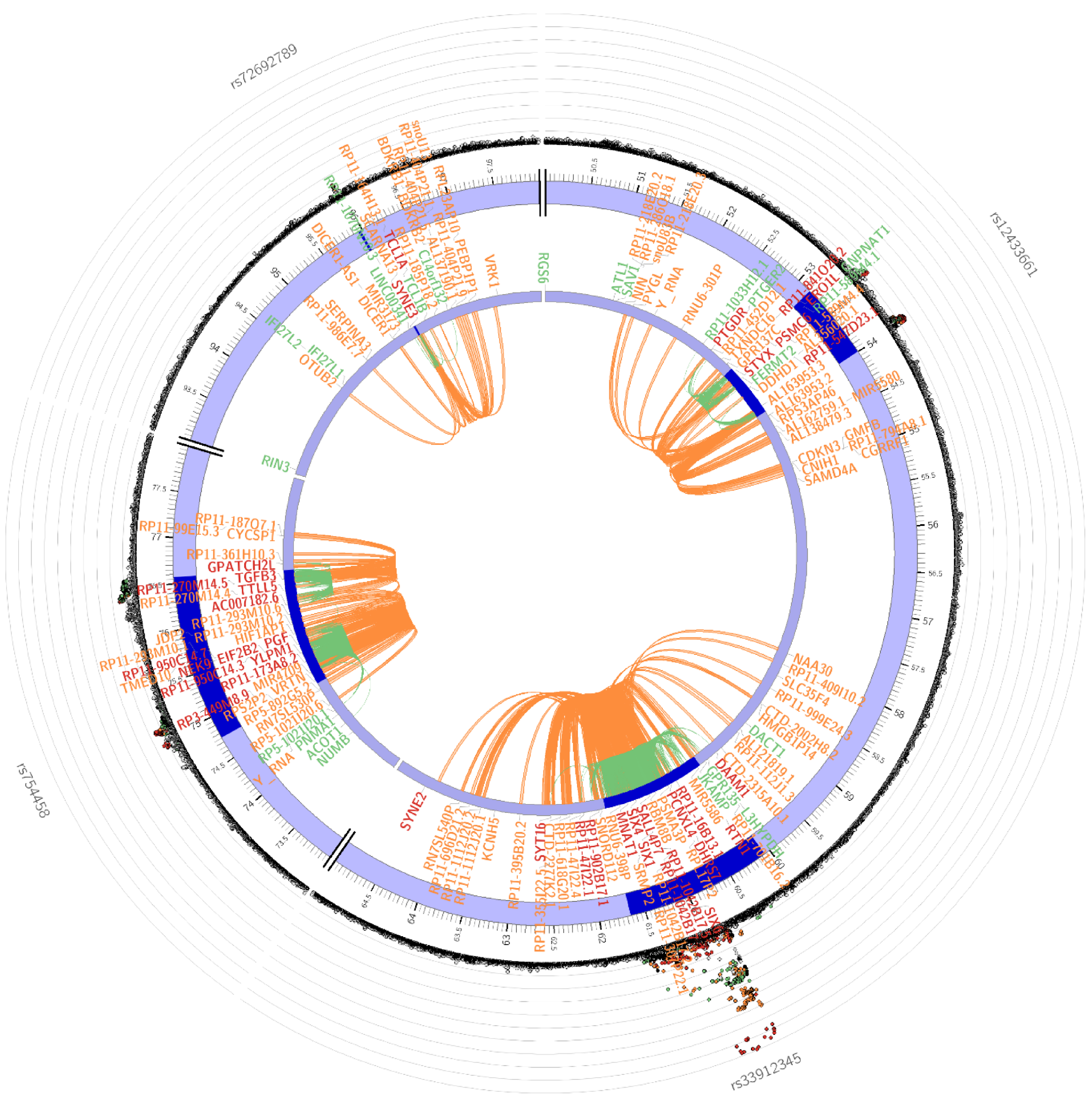

Chromosome 15

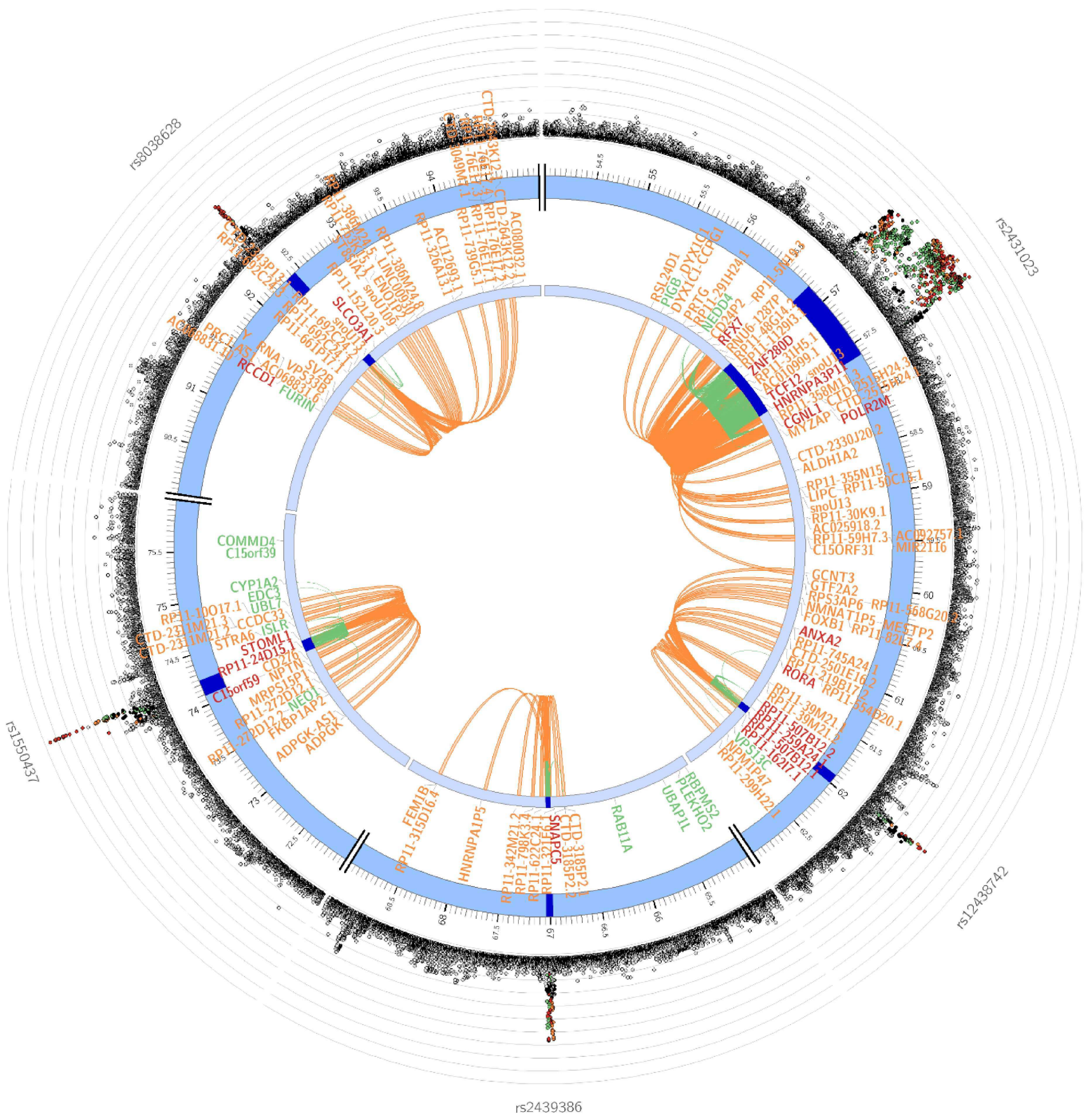

Chromosome 16

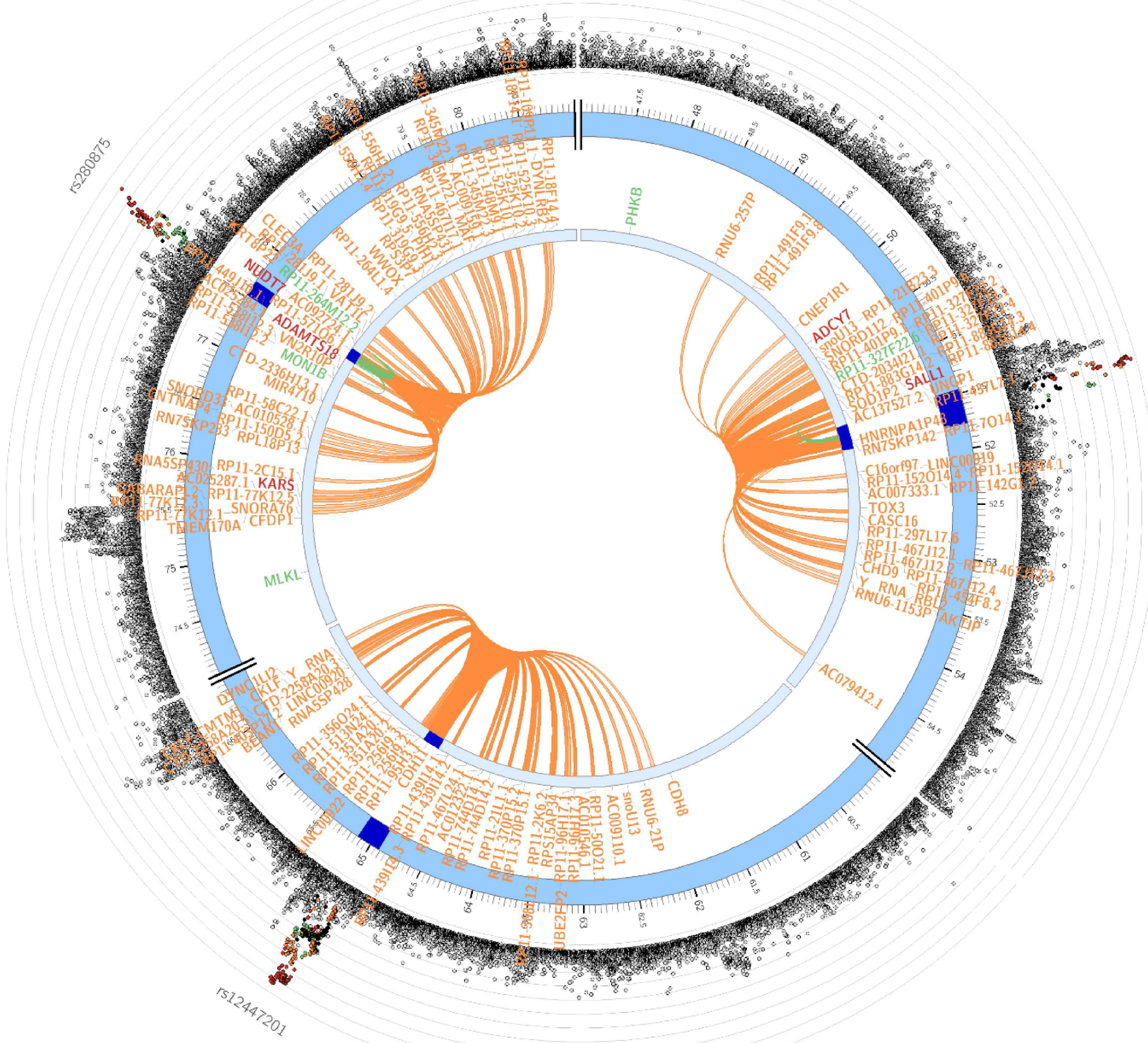

Chromosome 17

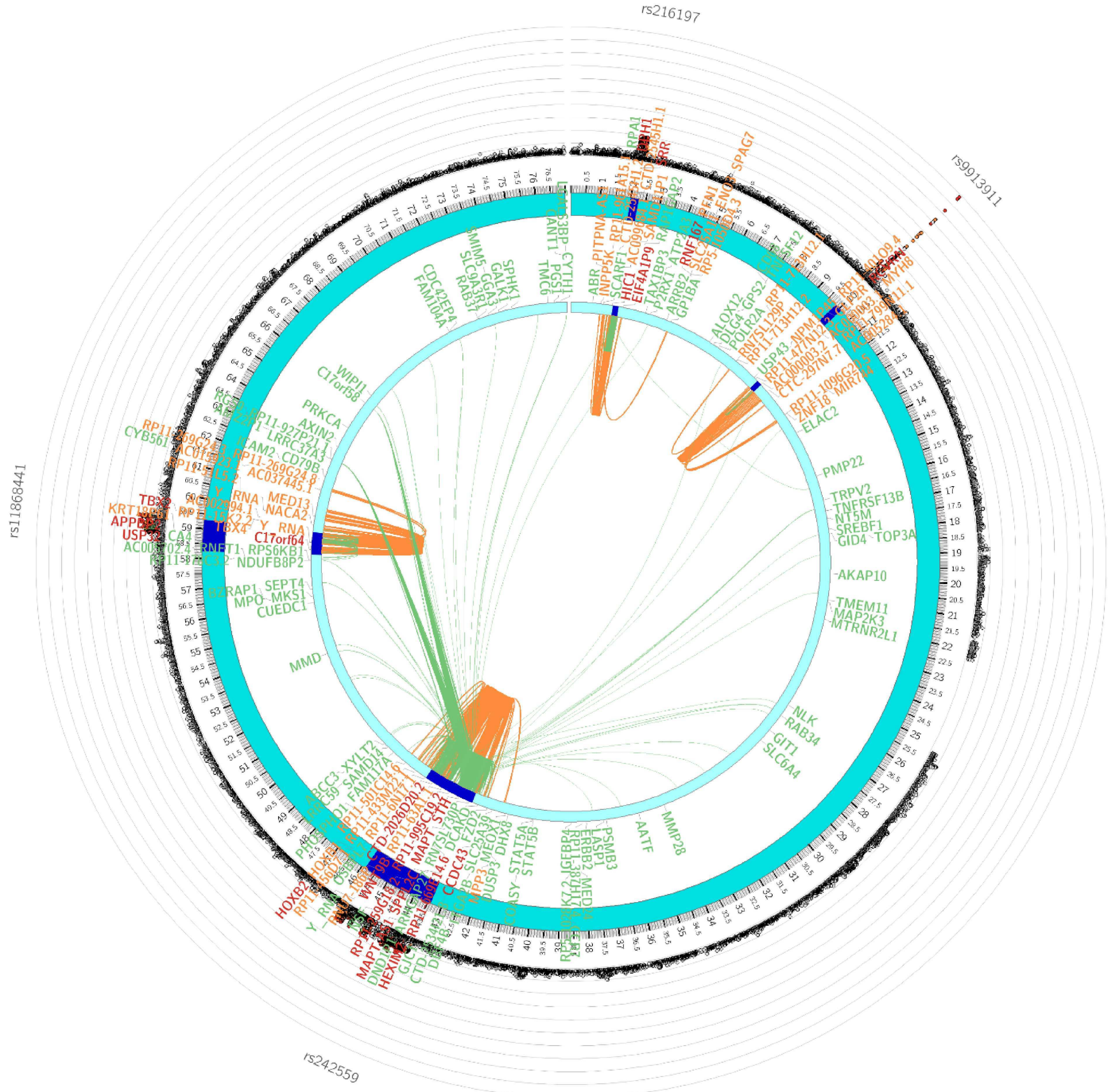

Chromosome 20

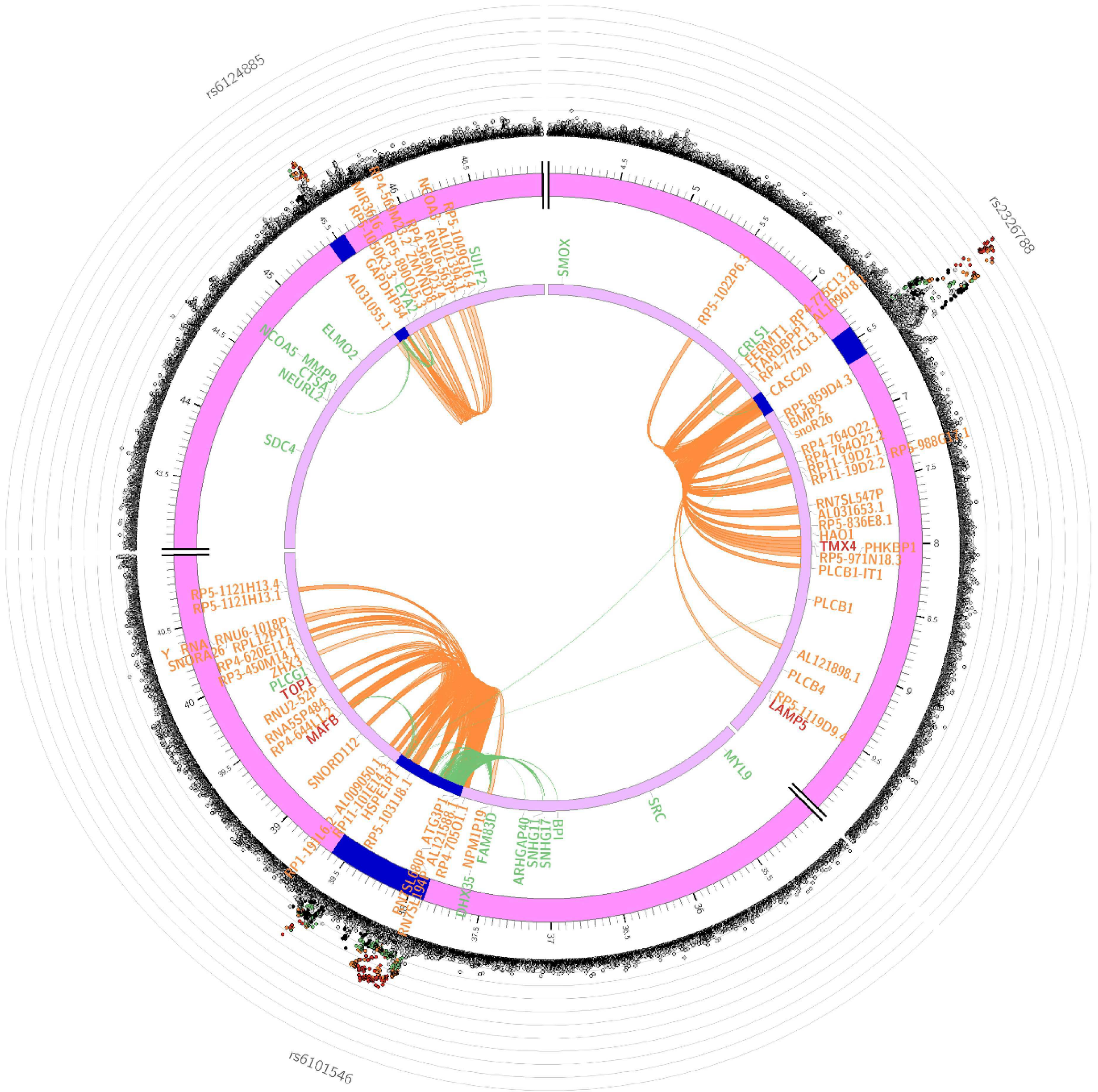

Chromosome 21

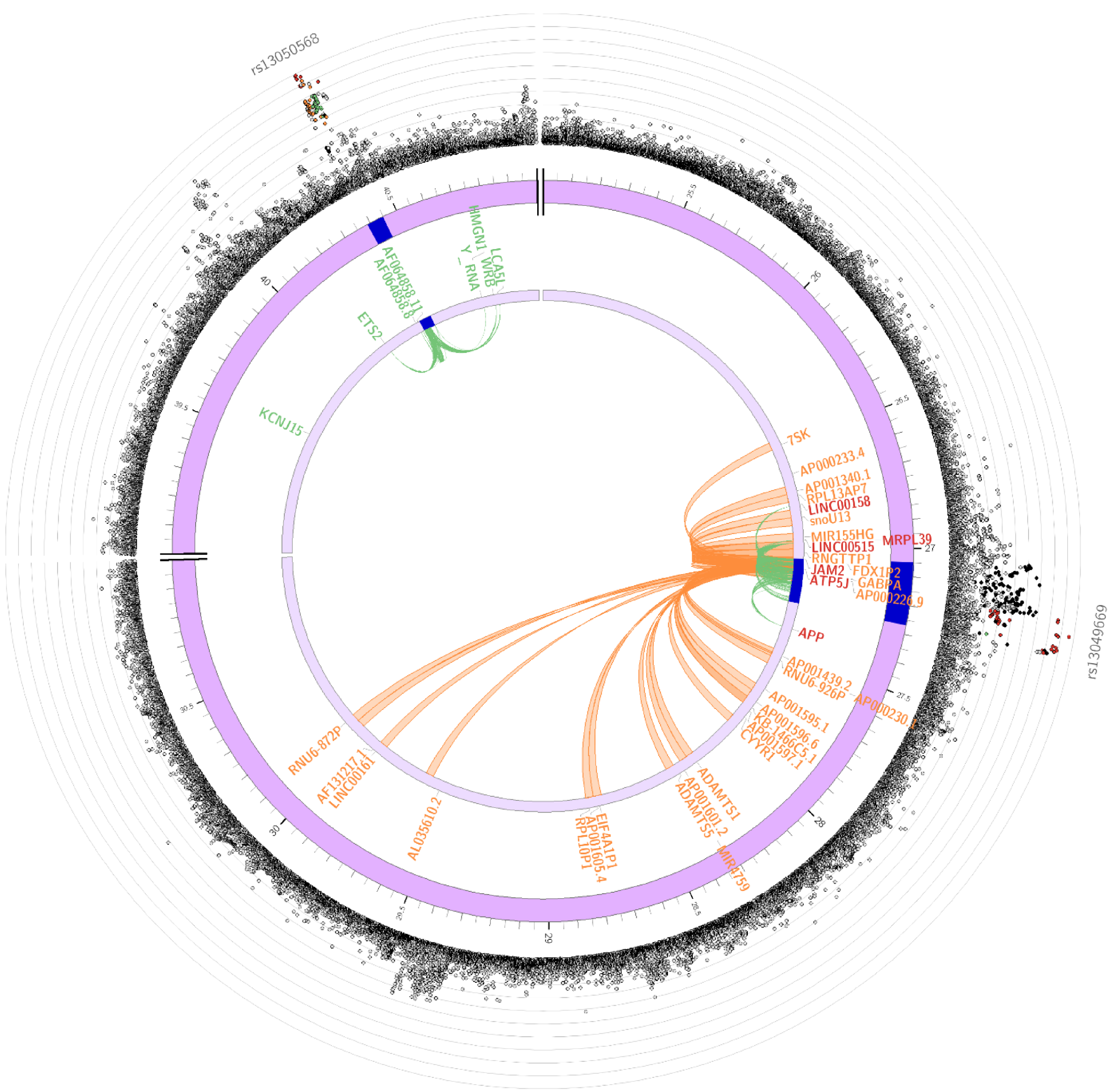

Chromosome 22

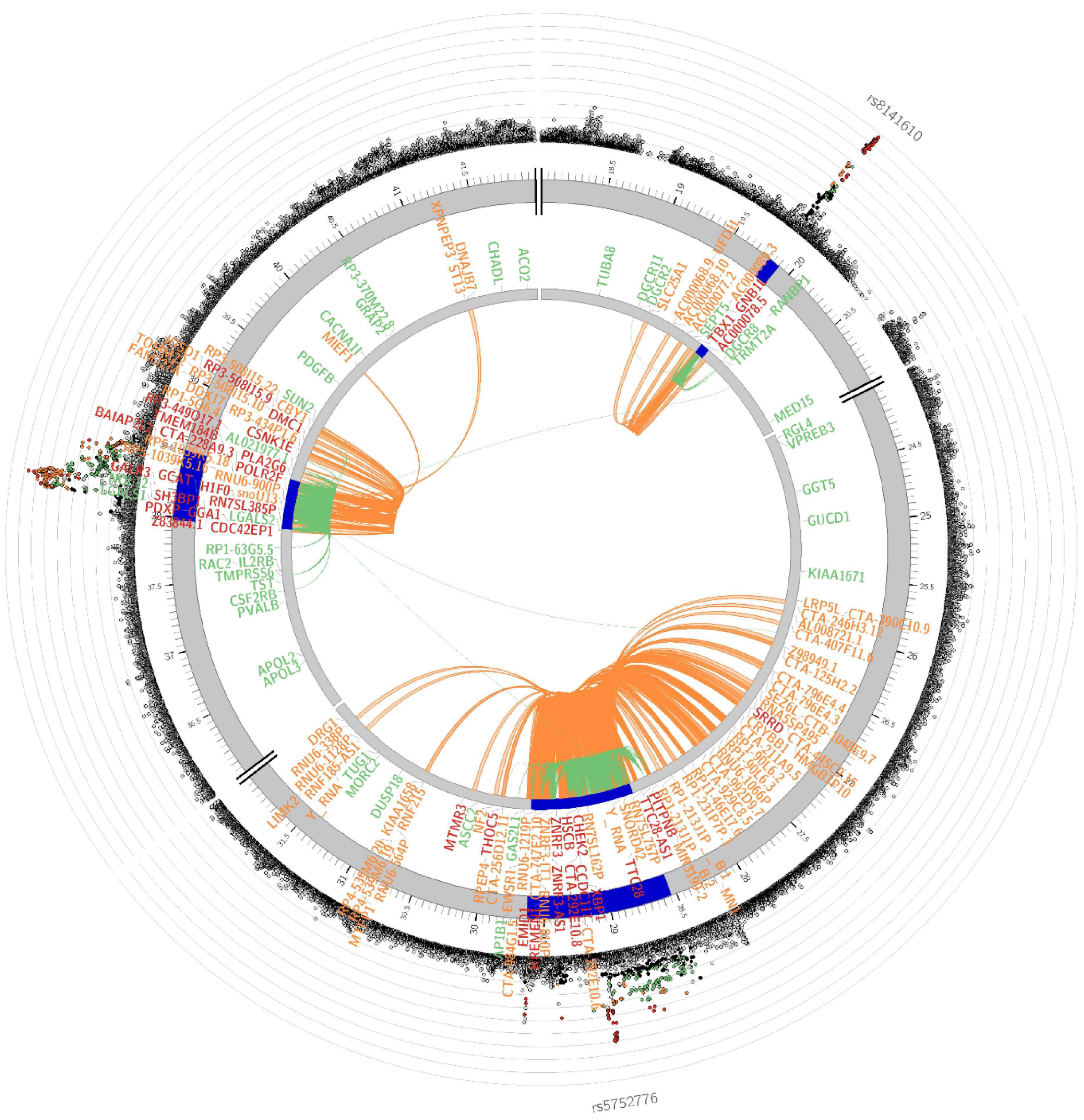

Chromosome 23

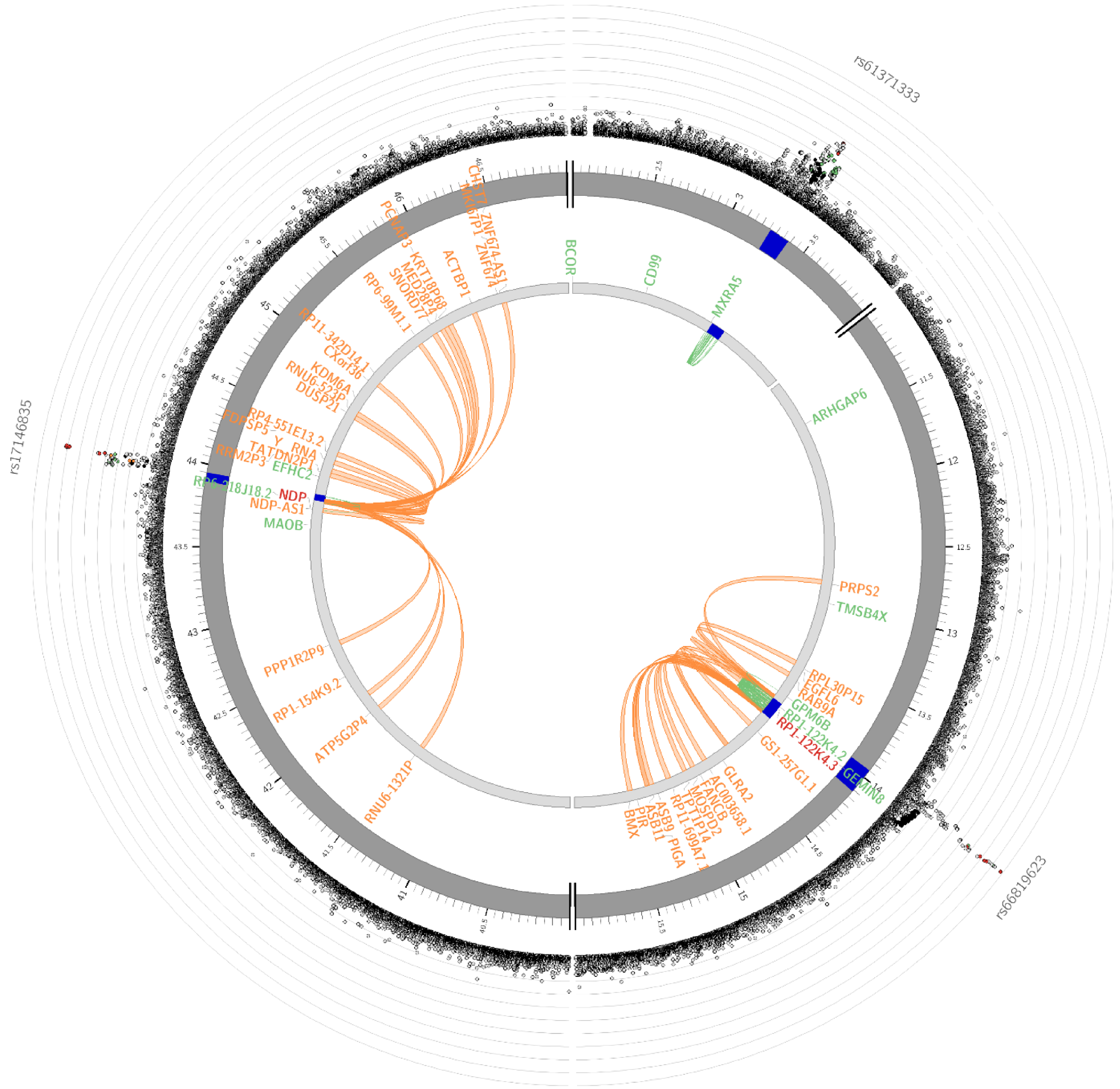

Supplementary Figure 7. Gene expression heatmaps in eye tissues.

a) Expression of the nearest genes to the most significant previously unknown risk SNPs.

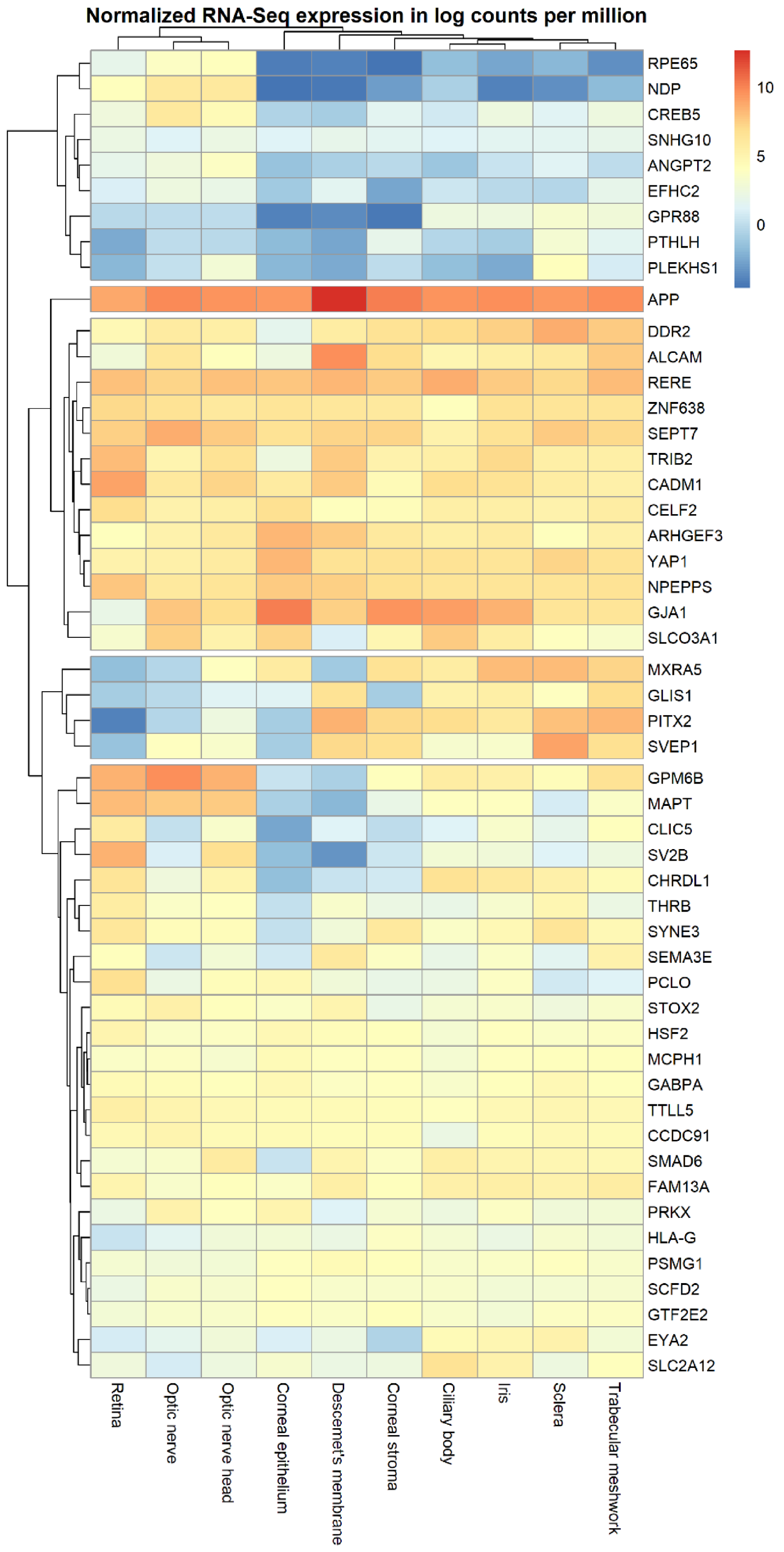

b) Differential gene expression of the nearest genes to the most significant previously unknown risk SNPs.

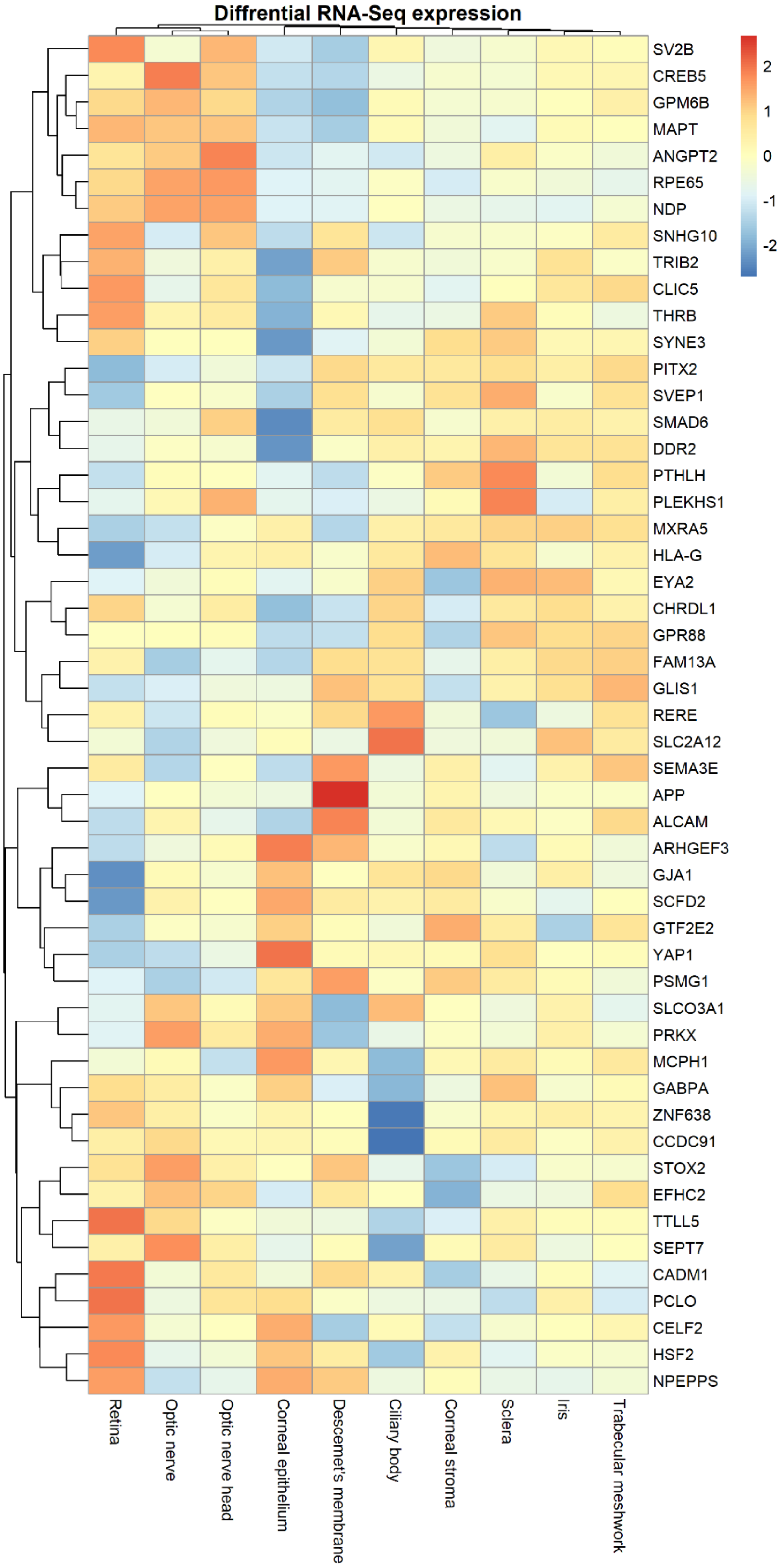

Supplementary Notes

Supplementary Notes 1: Study Cohorts

European Caucasian

Australian & New Zealand Registry of Advanced Glaucoma (ANZRAG)

In total, 3,071 cases and 6,750 unscreened controls of European descent were included from the Australian & New Zealand Registry of Advanced Glaucoma (ANZRAG)[1]. This dataset involves three phases of open-angle glaucoma (OAG) data collection. The first phase was previously published and comprises 1,155 advanced OAG cases and 1,992 controls genotyped on Illumina Omni1M or OmniExpress arrays (Illumina, San Diego, California, USA)[2]. The second phase includes a further 579 advanced OAG cases genotyped on Illumina HumanCoreExome array and 946 controls selected from parents of twins previously genotyped on the same array. The third phase comprises 1,337 OAG cases (11 advanced, 741 non-advanced, and 585 cases with visual field data unavailable) genotyped on Illumina HumanCoreExome array and 3,812 controls selected from a study of endometriosis previously genotyped on the same array. The diagnostic criteria have been described previously[2]. The Approval was obtained from the Human Research Ethics Committees of Southern Adelaide Health Service/Flinders University, University of Tasmania, QIMR Berghofer Medical Research Institute and the Royal Victorian Eye and Ear Hospital. Written informed consent was obtained from all participants. All the methods were carried out in accordance with relevant guidelines and regulations for human subject research, in accordance with the Declaration of Helsinki.

We used the same QC protocol for all the three phases of the ANZRAG OAG GWAS. Briefly, we performed QC using PLINK 1.9[3,4] by removing individuals with more than 3% missing genotypes, and SNPs with call rate less than 97%, minor allele frequency (MAF) < 0.01, and Hardy-Weinberg equilibrium $P < 0.0001$ in controls and $P < 5 \times 10{-}10$ in cases. The same QC protocol was used for case and control datasets before merging to avoid mismatches between the merged datasets. We used PLINK1.9 to compute identity by descent based on autosomal markers, with one of each pair of individuals with relatedness of greater than 0.2 removed within each phase of the ANZRAG data as well as between the three phases. PLINK 1.9 was used to compute principal components for all participants and reference samples of known northern European ancestry (1000 Genomes British, CEU, Finland participants). Participants with PC1 or PC2 values > 6 standard deviations from the mean of known northern European ancestry group were excluded.

Phasing of the genotyped SNPs was conducted using ShapeIT[5] and imputation was performed using Minimac3 through the Michigan Imputation Server[6], with the Haplotype Reference Consortium (HRC)[7] r1.1 as the reference panel. SNPs with imputation quality (r2) > 0.3 and MAF > 0.001 were carried forward for analysis.

We assessed associations between SNPs and OAG status adjusted for sex and the first six principal components under an additive genetic model using the dosage scores obtained from imputation. Association analysis was performed either using SNPTEST v2.5[8,9] or PLINK 1.9. The same approach was used to investigate the association of SNPs with NTG and HTG subsets within the ANZRAG dataset (821 NTG cases, 1,544 HTG cases, and 6,750 controls).

NEIGHBOR/MEEI/NHS/HPFS

All cases and controls met the clinical criteria used previously by the NEIGHBOR and GLAUGEN studies[10,11]. This study was approved by the Massachusetts Eye and Ear Infirmary (MEEI) institutional review board and all subjects signed consent forms approved by the local IRB prior to enrolling in the study. Briefly, POAG cases were defined as individuals for whom reliable visual field (VF) tests showed characteristic VF defects consistent with glaucomatous optic neuropathy. Individuals were classified as affected if the VF defects were reproduced on a subsequent test or if a single qualifying VF was accompanied by a cup-disc ratio (CDR) of 0.7 or more in at least one eye. The majority of cases (over 90%) met this definition, including 96% of the NEIGHBOR cases[11]; and all of the MEEI, NHS, HPFS, and WGHS cases. A small percentage (less than 10%) of the NEIGHBOR cases were defined by cup-to-disc ratio only because visual field data was not available, in some cases because of advanced disease (poor visual acuity) or other medical conditions. The CDR definition was > 0.7 in both eyes or CDR asymmetry between the two eyes of 0.2. Patients with signs of secondary causes for elevated IOP such as exfoliation syndrome or pigment dispersion syndrome or critically narrow filtration structures were excluded. The controls were selected to be representative of the age range and gender of the cases. Controls had IOP < 21 mmHg, as measured in a clinical setting, CDR of less than 0.6 and did not have a family history of glaucoma.

MEEI

A total of 829 samples that were previously collected and genotyped as part of the GLAUGEN study on the Illumina 660W_Quad_v1 array were included in this analysis[12]. QC was previously performed (SNP call rates >98%). All individuals had genotyping call

rate >99%, thus 485 POAG cases and 344 controls were analyzed at 495,160 SNPs. Principal components analysis was run using Eigenstrat and Eigenvectors were tested for association with disease status using a logistic regression model in Stata; Eigenvector 6 was significant. Imputation was performed using Minimac3 through the Michigan Imputation Server[6], with the Haplotype Reference Consortium (HRC)[7] r1.1 as the reference panel. SNPs with imputation quality (r2) > 0.3 and MAF > 0.001 were carried forward for analysis.

NEIGHBOR

A total of 4,422 samples that were previously collected and genotyped as part of the NEIGHBOR study on the Illumina 660W_Quad_v1 array[10] were included in this analysis. QC was previously performed (SNP and individual call rates >97%). Pairs of individuals with kinship coefficient >0.0312 were evaluated and the sample with lower genotyping efficiency from each pair removed. This resulted in 4383 individuals (2121 cases, 2262 controls) with genotype calls for 521,687 SNPs. Principal components analysis was run using Eigenstrat and Eigenvectors were tested for association with disease status using a logistic regression model in Stata; Eigenvectors 1,2,3, and 10 were significant. Imputation was performed using Minimac3 through the Michigan Imputation Server[6], with the Haplotype Reference Consortium (HRC)[7] r1.1 as the reference panel. SNPs with imputation quality (r2) > 0.3 and MAF > 0.001 were carried forward for analysis.

NHS/HPFS

POAG cases in NHS and HPFS have been described elsewhere[13]. This study was approved by the Partners institutional review board and the Harvard School of Public Health Institutional review board. Briefly participants completed surveys every two years

that included a question about receiving an eye care provider diagnosis of glaucoma. We retrieved the ophthalmic records from the diagnosing physician and reviewed them in a systematic fashion for POAG. We only included cases without evidence of secondary cause for elevated IOP, open filtration apparatus and reproducible visual field loss on reliable tests consistent with optic nerve disease that could not be explained by other causes.

Many of the participants in these cohorts have been genotyped as part of GWAS for particular diseases. POAG cases in particular were genotyped as part of The Gene, Environment Association Studies (GENEVA) Consortium under the acronym GLAUGEN (Glaucoma Gene and Environment) [14]. To maximize power, GWAS for different diseases were combined and imputed in a systematic manner to form a combined POAG case control set referred to in this study as NHS/HPFS. In order to decrease false positives in imputation, we used only SNPs that overlap when combining all the datasets. Since different platforms vary in SNP composition, we created two combined datasets based on the type of genotype platform: Affymetrix 6.0, and Illumina Hapmap Series. The details of combining and imputing the data have been previously published[15]. The Affymetrix dataset comprises four GWAS: NHS T2D (type 2 diabetes), HPFS T2D, NHS CHD (coronary heart disease), and HPFS CHD. The Illumina HapMap dataset is comprised of 12 datasets: NHS breast cancer, HPFS pancreatic cancer, NHS pancreatic cancer, NHS kidney stone, HPFS kidney stone, NHS2 kidney stone, NHS2 breast cancer, HPFS prostate cancer, NHS glaucoma, and HPFS glaucoma.

POAG cases were identified in both the Affymetrix and Illumina datasets (incident cases from 1986 – 2010). We implemented several exclusion criteria: we removed all GWAS

cancer cases (e.g., breast cancer GWAS cases), and all POAG cases diagnosed with cancer prior to their POAG diagnosis and all controls with prevalent cancer; we removed all controls without an eye exam in 2004-2010, non-Caucasians, and non-confirmed POAG cases (self-reports of glaucoma that were not confirmed by medical record review). The Affymetrix dataset is referred to as NHS/HPFS Affymetrix and the Illumina dataset is referred to as NHS/HPFS Illumina in this study.

We used KING[16] to calculate kinship coefficients for each pair of individuals both within the Affymetrix dataset, within the Illumina dataset and across the Affymetrix-Illumina datasets, using only the SNPs that overlapped between the two datasets (126,959 SNPs). Unresolved pairs with coefficient>0.0312 were removed (28 controls, 14 from Illumina, 14 from Affymetrix). After filtering, 80 cases and 3,289 controls were included in the Affymetrix dataset, and 382 cases and 2,219 controls in the Illumina dataset. Imputation was performed using Minimac3 through the Michigan Imputation Server[6], with the Haplotype Reference Consortium (HRC)[7] r1.1 as the reference panel. SNPs with imputation quality (r2) > 0.3 and MAF > 0.001 were carried forward for analysis.

We calculated PCs with EIGENSTRAT, using all of the SNPs available in the imputed dataset. We checked the top 10 PCs for association with POAG and included only PCs with p<0.05 in the analysis. In both datasets, we controlled for GWAS study (which inherently controlled for gender since each study only contained individuals of one gender or the other), and age at baseline. Since half of the GWAS studies contributing to the Affymetrix NHS/HPFS dataset were studies of type 2 diabetes, the Affymetrix dataset was enriched with diabetes cases. As there is evidence for an association between POAG and T2D[17], we controlled for diabetes status in the Affymetrix dataset. Association testing

was performed in a logistic regression model, adjusting for age, gender, BMI, diabetes status, and the top four PCs.

European Prospective Investigation into Cancer-Norfolk Eye Study (EPIC-Norfolk Eye Study)

The European Prospective Investigation into Cancer (EPIC) study is a pan-European prospective cohort study designed to investigate the etiology of major chronic diseases[18]. EPIC-Norfolk , one of the UK arms of EPIC, recruited and examined 25,639 participants between 1993 and 1997 for the baseline examination[19]. Recruitment was via general practices in the city of Norwich and the surrounding small towns and rural areas, and methods have been described in detail previously[20]. Since virtually all residents in the UK are registered with a general practitioner through the National Health Service, general practice lists serve as population registers. Ophthalmic assessment formed part of the third health examination and this has been termed the EPIC-Norfolk Eye Study[21]. In total, 8,623 participants were seen for the Eye Study, between 2004 and 2011. Ophthalmic examination included tonometry (Ocular Response Analyzer; Reichert, New York, USA; software V.3.01), optic disc photography (Nikon D80 camera; Nikon Corporation, Tokyo, Japan), scanning laser ophthalmoscopy (Heidelberg Retinal Tomograph 3; Heidelberg Engineering, Heidelberg, Germany) and nerve fiber layer assessment (Gdx-VCC; Zeiss, Dublin, California, USA). Participants meeting pre-defined criteria and an additional 1:10 participants underwent automated visual field testing (Humphrey 750i Visual Field Analyzer; Carl Zeiss Meditech Ltd, Welwyn Garden City, UK). The EPIC-Norfolk Eye Study was carried out following the principles of the Declaration of Helsinki and the Research Governance Framework for Health and Social Care. The study was approved

by the Norfolk Local Research Ethics Committee (05/Q0101/191) and East Norfolk & Waveney NHS Research Governance Committee (2005EC07L). All participants gave written, informed consent.

Ascertainment of POAG in the EPIC Norfolk third health examination has been described previously[22]. Participants with study results suspicious of glaucoma (using pre-defined criteria) were referred for further examination by a glaucoma specialist at the regional University Hospital[21]. Additionally, a diagnosis refinement process was undertaken by a second glaucoma specialist who independently reviewed the test results of all participants classified as glaucoma and a proportion of participants who were not classified as having glaucoma. POAG was defined as the presence of a glaucomatous optic disc together with either a corresponding visual field defect or otherwise unexplained non-specific visual field loss, open angles on gonioscopy, and absence of secondary causes of glaucoma. A glaucomatous disc was defined as one with focal or diffuse neuro-retinal rim thinning, and may possess, though not necessary for the definition, additional characteristic features such as bared circumlinear vessels, disc haemorrhages or nerve fiber layer defects. Pseudoexfoliative and pigmentary glaucoma were defined as secondary glaucoma in this study and therefore did not contribute to POAG cases. In addition to the POAG cases identified among the 8,623 participants attending the 3rd health examination, we included a further 397 POAG cases from the remainder of the cohort via linkage with hospital episode statistic data (ICD10 code H40.1). We defined controls as participants not meeting referral criteria for glaucoma on initial ophthalmic assessment and participants who attended the University Hospital for further examination and were not classified as having or being suspect for any type of glaucoma or ocular hypertension. Additionally, we

required controls to have intraocular pressure ≤ 21 mmHg and HRT vertical cup-to-disc ratio ≤ 0.5 to minimise the chance of false negatives.

Genotyping was undertaken using the Affymetrix UK Biobank Axiom Array. SNP exclusion criteria included: call rate < 95%, abnormal cluster pattern on visual inspection, plate batch effect evident by significant variation in minor allele frequency, and/or Hardy-Weinberg equilibrium P < 10-7. Sample exclusion criteria included: DishQC < 0.82 (poor fluorescence signal contrast), sex discordance, sample call rate < 97%, heterozygosity outliers (calculated separately for SNPs with minor allele frequency >1% and <1%), rare allele count outlier, and impossible identity-by-descent values. Following these exclusions, there were no ethnic outliers. After filtering, 664 cases and 5,630 controls were used in this study. Imputation was carried out using the Haplotype reference Consortium as a reference panel. Associations between SNPs and POAG were examined using logistic regression adjusted for age, sex and the first 5 principal components, assuming an additive model and using the imputed dosage data. Analyses were carried out using SNPTEST version 2.5.1.

UKBB POAG ICD10 code and self report cases

UK Biobank (UKBB) is a large-scale cohort study that included over 500,000 participants aged between 40-69 years in 2006-2010 from across the United Kingdom. The glaucoma cases definition and association analysis were described previously.[23] In this study, we defined UKBB POAG ICD-10 cases and self-reported glaucoma cases (exclude ICD-10 POAG cases) using updated phenotype data in April 2019. In brief, we identified ICD-10 POAG cases as those who had an ICD-10 diagnosis of ‘primary open angle glaucoma’, ‘other glaucoma’ or ‘glaucoma, unspecified’. We identified self-reported glaucoma cases

as those who (1) responded ‘glaucoma’ to the question ‘Has a doctor told you that you have any of the following problems with your eyes?’; or (2) responded ‘glaucoma’ to the question ‘In the touch screen you selected that you have been told by a doctor that you have other serious illnesses or disabilities, could you now tell me what they are? (non-cancer illness). In both ICD-10 POAG cases and self-reported glaucoma cases, we removed primary angle-closure glaucoma (PACG) cases. We then selected controls as those who reported that did not have any eye diseases. We used separate controls for ICD10 POAG cases and self-reported glaucoma cases in UKBB. With the ratio of 1:5 for ICD10 POAG cases and self-reported glaucoma cases, we spit the controls into two groups randomly with a ratio of 1:5. We then removed related participants ($\hat{\pi}$>0.2) within and between ICD-10 POAG GWAS and self-reported glaucoma GWAS. Finally, we included 1,448 ICD-10 POAG cases and 22,107 controls for ICD-10 POAG GWAS in UKBB, and 7,286 self-reported glaucoma cases and 107,362 controls for self-reported glaucoma GWAS. In the GWAS analysis, the dosage scores from imputation were used in the logistic regression models (PLINK version 2.0) adjusted for sex, age, and the first ten principal components. The study was approved by the National Research Ethics Service Committee North West – Haydock, and all study procedures were performed in accordance with the World Medical Association Declaration of Helsinki ethical principles for medical research. All participants provided informed written consent.

Kaiser Permanente GERA Cohort

The Genetic Epidemiology Research in Adult Health and Aging (GERA) cohort consists of 110,266 adults who consented to participate in the Research Program on Genes, Environment, and Health, established for members of the Kaiser Permanente Medical

Care Plan, Northern California Region (KPNC)[24,25]. The Institutional Review Board of the Kaiser Foundation Research Institute has approved all study procedures.

All GERA subjects included in this study had valid IOP measures, as previously described[26]. Briefly, non-numeric entries for IOP, extreme values (≤5 and >60 mm Hg), and measurements taken on a single eye were removed. Further, IOP measurements that were taken after initial prescription of IOP-lowering medications were excluded to avoid values influenced by treatment. Because IOP-lowering medications are almost always prescribed before IOP-lowering surgical interventions (i.e., laser trabeculoplasty, trabeculectomy, tube shunt procedures, etc.), we did not remove subjects who had these surgical interventions. Patients eligible for inclusion were identified from clinical diagnoses captured in the KPNC electronic health record (EHR) system. These clinical diagnoses were recorded in the EHR system as International Classification of Diseases, Ninth Revision (ICD-9) diagnosis codes. We defined glaucoma cases as having at least: (1) two diagnoses of POAG (ICD-9 codes 365.01, 365.1, 365.10, 365.11, and 365.15); or (2) two diagnoses of NTG (ICD-9 code 365.12); or (3) one diagnosis of POAG and one diagnosis of NTG. In all cases, at least one of the diagnoses was made by a Kaiser Permanente ophthalmologist. Further, the cases did not have any diagnosis of other subtypes of glaucoma (e.g., pseudoexfoliation, pigmentary, or PACG; ICD-9 codes 365.52, 365.13, and 365.2, respectively). After excluding subjects who have one or more diagnosis of any type of glaucoma (ICD-9 code, 365.xx), our control group included all the non-cases. Subjects who had no diagnosis of any type of glaucoma (any ICD-9 code 365.xx other than 365.04) but did have a diagnosis of OHTN (ICD-9 code 365.04), were included as controls. In total, 4,258 POAG and 51,986 controls from GERA (non-Hispanic white and

East Asian ethnic groups) were included in this study.

GERA individuals' DNA samples were extracted using Oragene kits (DNA Genotek Inc., Ottawa, ON, Canada) at KPNC and genotyped at the Genomics Core Facility of UCSF. DNA samples were genotyped at over 665 000 genetic markers on four race/ethnicity-specific Affymetrix Axiom arrays (Affymetrix, Santa Clara, CA, USA) optimized for European, Latino, East Asian, and African-American individuals[27,28]. We performed genotype quality control (QC) procedures for the GERA samples on an array-wise basis[25]. Briefly, we included genetic markers with initial genotyping call rate ≥ 97%, genotype concordance rate > 0.75 across duplicate samples, and allele frequency difference ≤ 0.15 between females and males for autosomal markers. Approximately 94% of samples and over 98% of genetic markers assayed reached QC procedures. Moreover, genetic markers with genotype call rates < 90% were excluded, as well as genetic markers with a MAF < 1%. Following the pre-phasing of genotypes with Shape-IT v2.r72719[5], imputation was performed using Minimac3 through the Michigan Imputation Server[6], with the Haplotype Reference Consortium (HRC)[7] r1.1 as the reference panel. SNPs with imputation quality (r2) > 0.3 and MAF > 0.001 were carried forward for analysis.

A logistic regression of POAG and each SNP was performed with the following covariates: age; sex; and ancestry principal components (PCs). The top 10 ancestry PCs were included as covariates for the non-Hispanic white ethnic group, whereas the top 6 ancestry PCs were included for the East Asian ethnic group. To adjust for genetic ancestry, the percentage of Ashkenazi (ASHK) ancestry was also included as a covariate for the non-Hispanic white ethnic group analysis.

King's College London (KCL)

The POAG case–control cohort consists of 576 cases and 287 controls of European ancestry and 298 cases and 194 controls of West African ancestry residing in the United Kingdom. European controls, high-tension glaucoma cases, and a small number of normal-tension cases were recruited in South London at St Thomas' Hospital and the Princess Royal University Hospital, UK. The majority of European normal-tension cases were patients at the University Eye Hospitals in Tuebingen and Wuerzburg, Germany. All African cases, and some of the controls were recruited in South London at St Thomas' Hospital, additional controls were used from the SOUL-D study (The South London Diabetes Study). Patients were included in this study if they had visual field loss in at least one eye attributed to glaucoma by a glaucoma specialist, had a VCDR of more than 0.6, were receiving intraocular-lowering medication (or had previous surgery), and had open drainage angles on gonioscopy. European controls were also examined by an ophthalmologist and were confirmed to be either free from any eye disease, or only had cataracts with no evidence of glaucoma but their visual field was not tested. African controls from St Thomas' followed the same clinical screening, while controls from the SOUL-D study were not screened but did not report glaucoma. Diagnostic criteria for HTG and NTG included a presence of both a VCDR>=0.6 and a visual field defect. In addition to this, HTG cases had a highest recorded, pre-treatment IOP(GAT)>21mmHg, and NTG cases had a highest recorded, pre-treatment IOP(GAT)<21mmHg. All subjects were genotyped in the same batch on the Illumina Human Omni Express Exome 8v1-2 chip. The African cohort was confirmed to be of West-African descent through principal component analysis with samples from the 1000G Nigerian, Gambian and Sierra Leonne populations. Imputation was performed using the Michigan Imputation Server. European

and African subjects were imputed separately. The HRC reference panel was used for Europeans and the 1000G phase 3 panel was used for Africans. SNPs were filtered using a minor allele frequency of >0.001 and imputation quality score >0.3. GWAS was conducted in Plink 2 using gene dosage in an additive model, logistic regression (Firth). Age, sex and the first 6 principal components were used as covariates (sex was excluded as a covariate for the sex-stratified analyses).

Blue Mountain Eye Study (BMES)

The BMES is a population-based eye assessment of a representative older Australian community sample. During the period 1992-1994, 3654 residents (57% male and 43% females) aged 49-97 were examined (BMES-1). The study was approved by the Western Sydney Area Health Service Human Ethics Committee, and written informed consent was obtained from all participants. Open-angle glaucoma was diagnosed when glaucomatous defects on the Humphrey 30-2 test matched the optic disc changes, without regard to the intraocular pressure level.

The BMES cohort included 107 BMES glaucoma cases and 600 population controls (from BMES) genotyped on Illumina Human 610 Quad Array and imputed against HRC reference panel. The methods used for imputation and statistical analysis of this cohort were the same as the ANZRAG cohort. After quality control (QC), 540,035 SNPs were used as the basis of imputation for the BMES cohort.

Southampton

Primary open-angle (POAG) and normal tension glaucoma patients were recruited from the Southampton University Hospital Trust Eye Clinic and satellite regional glaucoma

clinics. Ethical approval for the collection of patient information and blood samples was provided by the Southampton and South West Hampshire Local Research Ethics Committee (05/Q1702/8)and Cohort Recruitment commenced in August 2005.Each patient was examined by an experienced glaucoma specialist. Diagnoses were made on the basis of characteristic visual field loss/glaucomatous optic disc damage/increased IOP. Patients presenting with narrow-angle, developmental or secondary glaucoma or any other known abnormalities of the anterior segment were excluded. Patients with unambiguous glaucoma, but normal tension were included in sample collection later. Furthermore, to select for patients with typical POAG or normal-tension glaucoma (NTG), only patients diagnosed over the age of 40 years were included. Both conditions are rare before this age. DNA was extracted according to the standard methods, dissolved in TE buffer, and stored at-20℃.Primary open angle glaucoma patients were genotyped on the AffymetrixSNP6.0 array, all data were exported on the forward strand. These data were combined with the Affymetrix SNP 6.0 data publically available for the WTCCC2 controls. The Genome-wide association data was further filtered to ensure removal of individuals with more than 5% missing genotypes, SNPs with more than 3%missing samples, SNPs with a minor allele frequency of <1% and a Hardy-Weinberg p-value $<1x10^{-6}$.SNPs were all on the forward strand and locations were lifted over from hg18 to hg19 using the UCSC liftover tool. SNPs with complementary alleles were also excluded (A/T and G/C).

The data used for imputation included 941 cases and 1,557 controls, and 533,774 SNPs. Pre-phasing was carried out using Shapeit (v2, r790)and imputation was carried out using Minimac3 through the Michigan Imputation Server[6], with the Haplotype Reference Consortium (HRC)[7] r1.1 as the reference panel. SNPs with imputation quality (r2) > 0.3

and MAF > 0.001 were carried forward for analysis. Case-control analysis was carried out using logistic regressions with sex as a covariate, using PLINK (v1.90b3b 64-bit (15 Jan2015)).

Gutenberg Health Study (GHS)

The GHS is a population-based, prospective, observational cohort study in the Rhine-Main Region in midwestern Germany with a total of 15,010 participants and follow-up after five years. The study sample is recruited from subjects aged between 35 and 74 years at the time of the exam. The sample was drawn randomly from local governmental registry offices and stratified by gender, residence (urban and rural) and decade of age. Exclusion criteria were insufficient knowledge of the German language to understand explanations and instructions, and physical or psychic inability to participate in the examinations in the study center. The study was approved by the Medical Ethics Committee of the University Medical Center Mainz and by the local and federal data safety commissioners. Genotyping was carried out using Affymetrix Genome-Wide Human SNP 6.0 Array. Imputation was performed using Minimac3 through the Michigan Imputation Server[6], with the Haplotype Reference Consortium (HRC)[7] r1.1 as the reference panel. SNPs with imputation quality (r2) > 0.3 and MAF > 0.001 were carried forward for analysis. After QC, 47 cases and 2,731 controls were included in this study. POAG cases were defined based on the ISGEO classification. Association testing was performed using Plink2, assuming an additive genetic model with age, sex, and the top three PCs fitted as covariate.

Erasmus Rucphen Family (ERF) study

The ERF study is a family-based cohort in a genetically isolated population in the southwest of the Netherlands with over 3,000 participants aged between 18 and 86 years[29,30]. In the region of the ERF population, a total of 110 patients with glaucoma who did not participate in the ERF study were recruited in three local hospitals. Their visual fields were tested with standard automated perimetry (Humphrey Field Analyzer c24-2 SITA Standard test program) or the Octopus 101 (G2 program with TOP strategy) (Haag-Streit, Bern, Switzerland). The diagnosis of glaucoma was made by the patient's ophthalmologist and confirmed by a glaucoma specialist (HGL). It was based on a glaucomatous appearance of the optic disc (notching or thinning of the neuroretinal rim), combined with a matching glaucomatous visual field defect, and open-angles seen by gonioscopy. Participants from the ERF study were used as control group (n = 1999). Genotyping was performed with the 318K array of the Illumina Infinium II whole-genome genotyping assay (HumanHap300-2). Samples with low call rate (<97.5%), with excess autosomal heterozygosity (>0.336), or with sex-mismatch were excluded. A set of genotyped input SNPs with call rate >98%, with MAF >0.01, and with HWE p-value >10−6 was used for imputation. Imputation with HRC was facilitated by the Michigan Imputation server, file preparation was done using scripts provided online (HRC Imputation preparation and checking: http://www.well.ox.ac.uk/~wrayner/tools/; v4.2.1). Filtered genotypes were uploaded. The server uses SHAPEIT2 (v2.r790) to phase the data and Minimac 3 for imputation to the HRC reference panel (v1.0). We used the imputed dosages returned by the service. Association analyses were performed in RvTest --meta score option; to adjust for familial relationships in ERF, the kinship matrix estimated from the genotyped data was used, analyses were also adjusted for age and sex. The study

was approved by the medical ethics committee of the Erasmus Medical Center, Rotterdam, and written consent was obtained from all subjects.

Rotterdam Study I

The Rotterdam Study is a population-based study established in Rotterdam, the Netherlands[31]. It consists of three cohorts. The original cohort, Rotterdam Study I (RS-I), started in 1990 and includes 7,983 subjects aged 55 years and older. Participants visiting the research center underwent an extensive ophthalmic examination. Details of the eye examinations have been described elsewhere. POAG was defined as glaucomatous visual field loss (GVFL) with glaucomatous optic nerve neuropathy (GON)[32]. DNA was isolated from whole blood according to standard procedures. Genotyping of SNPs was performed using the Illumina Infinium II HumanHap550 array (RS-I). Samples with low call rate (<97.5%), with excess autosomal heterozygosity (>0.336), or with sex-mismatch were excluded, as were outliers identified by the identity-by-state clustering analysis (outliers were defined as being >3 standard deviation (s.d.) from population mean or having identity-by-state probabilities >97%). A set of genotyped input SNPs with call rate >98%, MAF >0.001 and Hardy-Weinberg Equilibrium (HWE) p-value >10-6 was used for imputation. Imputation with HRC was facilitated by the Michigan Imputation server, file preparation was done using scripts provided online (HRC Imputation preparation and checking: http://www.well.ox.ac.uk/~wrayner/tools/; v4.2.1). Filtered genotypes were uploaded. The server uses SHAPEIT2 (v2.r790) to phase the data and Minimac 3 for imputation to the HRC reference panel (v1.0). We used the imputed dosages returned by the service. GWAS analyses were performed using Rvtest --meta score option. The analyses were adjusted for age, sex, and the first five principal components. The

Rotterdam Study has been approved by the institutional review board (Medical Ethics Committee) of the Erasmus Medical Center and by the review board of The Netherlands Ministry of Health, Welfare and Sports.

Geisinger

Geisinger cohort consists of a sample size of 43,982 who consented to participate in the MyCode Community Health Initiative program. Within this dataset, the POAG case-control group consists of 664 cases and 5904 controls of European ancestry. All samples were genotyped on Illumina Human Omni Express genotyping platform and imputation was carried out using Michigan Imputation Server with HRC reference panel. Markers with imputation quality (r2) >= 0.3 and MAF >= 0.01 were carried forward for analysis. Additionally a HWE p-value < 1e-07, a marker call rate of 99% and a sample call rate of 90% were applied as a further filtration criteria. The analysis was run in unrelated samples who are 18 years or older and was adjusted for the covariates of first four principal components and age. The study was approved by Geisinger Institutional Review Board, and written consent was obtained from all subjects.

FinnGen

Patients and control subjects in FinnGen provided informed consent for biobank research, based on the Finnish Biobank Act. Alternatively, separate research cohorts, collected prior the start of FinnGen (August 2017), were collected based on study-specific consents and later transferred to the Finnish biobanks after approval by Fimea, the National Supervisory Authority for Welfare and Health. Recruitment protocols followed the biobank

protocols approved by Fimea. The Coordinating Ethics Committee of the Hospital District of Helsinki and Uusimaa (HUS) approved the FinnGen study protocol Nr HUS/990/2017.

The FinnGen project is approved by Finnish Institute for Health and Welfare (THL), approval number THL/2031/6.02.00/2017, amendments THL/1101/5.05.00/2017, THL/341/6.02.00/2018, THL/2222/6.02.00/2018, THL/283/6.02.00/2019), Digital and population data service agency VRK43431/2017-3, VRK/6909/2018-3, the Social Insurance Institution (KELA) KELA 58/522/2017, KELA 131/522/2018, KELA 70/522/2019 and Statistics Finland TK-53-1041-17.

The Biobank Access Decisions for FinnGen samples and data utilized in FinnGen Data Freeze 4 include: THL Biobank BB2017_55, BB2017_111, BB2018_19, BB_2018_34, BB_2018_67, BB2018_71, BB2019_7 Finnish Red Cross Blood Service Biobank 7.12.2017, Helsinki Biobank HUS/359/2017, Auria Biobank AB17-5154, Biobank Borealis of Northern Finland_2017_1013, Biobank of Eastern Finland 1186/2018, Finnish Clinical Biobank Tampere MH0004, Central Finland Biobank 1-2017, and Terveystalo Biobank STB 2018001.

For this study we used a sample of 1,824 POAG cases and 93,036 controls of European ancestry. POAG was defined based on the following ICD codes: ICD10 H401, ICD9 3651, and ICD8 37510,37512,37513. Individuals with the other types of glaucoma were excluded from controls. Samples were genotyped on various Affymetrix and Illumina platforms (FinnGen 1, HumanCoreExome, HumanOmniExpress, PsychChip, Global Screening Array, 610 Quad, and 250K) and imputation was carried out using 3,775 Finnish WGS samples as reference panel. Markers with imputation quality (r2) >= 0.3 and

MAF >= 0.001 were carried forward for analysis. Association testing was performed using SAIGE 0.29.4, assuming an additive genetic model with age, sex, the top 10 PCs, and genotyping batch fitted as covariate.

Asian

Singapore Chinese Eye Study (SCES)

POAG including NTG patients were from the Singapore National Eye Center glaucoma clinics. Ethical approval for the collection of patient information and blood samples was provided by the local institutional ethics review committee (CIRB) and study was conducted in accordance with revised Declaration of Helsinki. Patients with POAG over the age of 40 years old were recruited. Patients with POAG were defined by the following criteria: the presence of glaucomatous optic neuropathy (defined as a loss of neuroretinal rim with a vertical cup: disc ratio of >0.7 or an inter-eye asymmetry of >0.2, and/ or notching attributable to glaucoma) with compatible visual field loss, open angles on gonioscopy, and absence of secondary causes of glaucomatous optic neuropathy. POAG patients with a mean IOP without treatment that was consistently <21 mm Hg on diurnal testing were classified as having NTG, whereas those with a mean IOP without treatment that was consistently >21 mm Hg on diurnal testing were classified as having HTG. Diurnal IOP measurements were recorded hourly between 8.00 am to 5.00 pm with non-contact air puff tonometry (Topcon CT-80 Computerized Non-Contact Tonometer, Topcon). Patients were excluded if they were unable to give written informed consent or had either neurological/retinal disease that had visual field sequelae, or secondary glaucoma (such as pigmentary, uveitic, neovascular or post-traumatic).

1224 Singaporean Chinese POAG patients were recruited under the above criteria, and their genomic DNA samples were genotyped using the Illumina Human OmniExpress Beadchips. 1033 cases passed quality control filters and were brought forward for downstream analyses. The final sample cohort with clear sub-diagnosis comprised of 289 NTG and 709 HTG subjects. Controls were ascertained from an on-going population based study of Chinese persons aged 40 years and older (the Singapore Chinese Eye Study [SCES])[33]. The SCES is a population-based, cross-sectional study of Chinese adults residing in the south- western part of Singapore. 2587 SCES samples were genotyped with Illumina Human OmniExpress or Human610-Quad BeadChips. 2371 that passed quality check were used in subsequent genetic analyses. The imputation and phasing of genotypes were carried out using IMPUTE2 (http://mathgen.stats.ox.ac.uk/impute/impute_v2.html) with cosmopolitan population haplotypes based on data from 2535 individuals from 26 distinct populations around the world obtained from the 1000 Genomes project Phase 3 (Jun 2014) release for reference panel construction. SNPs with imputation quality (r2) > 0.3 and MAF > 0.001 were carried forward for analysis. Association testing was performed using EPACTS (https://genome.sph.umich.edu/wiki/EPACTS) in a logistic regression model with age, sex, and the first PC fitted as covariate.

The Chinese University of Hong Kong (CUHK)

Patients with POAG were defined using the same criteria as described for the Singaporean POAG collection. All subjects of the Hong Kong study population were recruited from the Chinese University of Hong Kong Eye Centre. The study protocol was approved by the Ethics Committee for Human Research, the Chinese University of Hong

Kong following the tenets of the Declaration of Helsinki. A total of 217 POAG patients and 397 controls of Han Chinese ancestry were used in this study. The Hong Kong control subjects were recruited in a hospital-based manner. They were all given complete ocular examinations, and confirmed to have no sign of glaucoma, angle closure or narrow angle, or other major eye diseases except for mild cataract and mild refractive errors. Control subjects were recruited from elderly people aged ≥ 60 years to ensure they were at least free of early-onset major eye diseases. They had IOP < 21 mmHg, and had no known family history of glaucoma. Genotyping was carried out using Illumina Humancnv370-quad V3.0. Imputation was performed using Minimac3 and 1000G phase 3 as the reference panel. SNPs with imputation quality (r2) > 0.3 and MAF > 0.001 were carried forward for analysis. Association testing was performed in a logistic regression model with age, sex, and the top six PCs fitted as covariate.

Japanese Tohoku

All the POAG patients were recruited at the Institutes related to Tohoku University. The study protocol followed the tenets of the Declaration of Helsinki and was approved by the Institutional Review Board of the Tohoku Graduate School of Medicine. All participants in this study were at the age of 35 years or older and of Japanese residents. All of the subjects with POAG were diagnosed by glaucoma specialists and fulfilled the following diagnostic criteria: presence of glaucomatous optic disk changes, including neuroretinal rim thinning, notching, or cupping; presence of visual field defect that could be attributed to the optic disk changes; and no history of secondary, angle closure, or congenital glaucoma. The control subjects recruited at the Tohoku Medical Megabank Organization

as a part of prospective cohort study were considered to have no POAG based on self-report.

A total of 1267 patients with POAG and 2517 healthy controls were recruited above the criteria. DNA samples from the participants were collected from whole blood according to standard procedures. Japonica array^{TM} (Toshiba, Tokyo, Japan) is a custom-designed array optimized for the Japanese population based on the information from the reference panel from 1,070 Japanese. Genome-wide genotype data set was obtained using this SNP array for 1267 patients with POAG according to the manufacturer's instructions. 38 case samples that did not satisfy the quality control criteria (DishQC > 0.82 and call rates > 0.970) and 38 case samples from pairs with a relatedness measure (pi-hat) value >0.2 were excluded, thus resulting in a dataset comprising 1191 cases. As a control, genotype data from 2434 healthy subjects collected previously through a prospective cohort study were used.

SNP quality control was applied for the imputation procedure. We used info score using IMPUTE2, and considered a SNP with info score greater than 0.9 as an acceptable well-imputed variant in this study. Autosomal SNPs that were assigned to 'Recommended' by the Ps_Classification program in the SNPolisher package (Affymetrix) were selected. We applied the following thresholds for quality control in further data cleaning: Hardy–Weinberg equilibrium with a P value <0.0001 for control samples, call rate for each SNP > 0.970, and minor allele frequencies <0.005. As a result, a total of 608329 SNPs on autosomal chromosomes passed the quality control filters and were used for whole genome imputation. Prephasing was first conducted with these SNPs by SHAPEIT (v2.r837). Genotype imputation was performed on the phased genotypes with IMPUTE2

(ver. 2.2.2) by using a phased reference panel of the 1000 Genomes project Phase3. SNPs with imputation quality (r2) > 0.3 and MAF > 0.001 were carried forward for analysis. Association analysis was performed in logistic regression model with sex and top six PCs as covariates.

Biobank Japan

The Biobank Japan (BBJ; http://biobankjp.org) is a large, patient-based biobank which started at the Institute of Medical Science, the University of Tokyo in 2003, consisted of approximately 267,000 individuals with 51 target diseases including glaucoma. The definition of POAG, NTG, and HTG are fully described in our previous reports[34,35]. As the controls of present study, we randomly selected 30,000 samples from the participants of the BBJ using '--thin-indiv-count' option implemented by PLINK1.9, and integrated them with genotype data of general Japanese subjects obtained from Pharma SNP consortium (N=946). After the quality controls, 3,979 cases and 30,278 controls were included in the association analysis. All of these participants were genotyped by (a) a combination of Illumina Human OmniExpress BeadChip and Infinium HumanExome BeadChip or (b) Infinium OmniExpressExome. We phased genotypes using Eagle2, and imputed using 1000 Genomes Project Phase 3 (N = 2,504) using minimac3 as we described elsewhere[36,37]. Association analysis was performed in logistic regression model by PLINK2 using age, sex, top six PCs as covariates. The study was approved by the ethical committees at the Institute of Medical Science, the University of Tokyo, and the Center for Genomic Medicine, RIKEN, Yokohama Institute, Japan. Written consent was obtained from all subjects.

African

GIGA (TZ, SAC, SAB)

The Genetics In Glaucoma patients from African descent study (GIGA) is a multicenter case–control study comprising POAG patients and controls from South Africa and Tanzania. Participants from Black African and South African Coloured ancestry were ascertained from the ophthalmology outpatient department of the Groote Schuur Hospital in Cape Town, South Africa (N_{cases} = 327; $N_{controls}$= 194), and from hospitals in Tanzania: Muhimbili National Hospital and CCBRT Disability Hospital in Dar es Salaam (N_{cases} = 395; $N_{controls}$ = 382). The study was conducted according to the guidelines for human research by the National Institute for Medical Research in Tanzania. Ethical approval was obtained from the institutional review boards at each study site, and written informed consent was provided by each participant.

POAG cases met category 1 or 2 of the ISGEO classification for open-angle glaucoma[38]. In brief, cases had either a definite visual field defect and Vertical Cup Disc Ratio (VCDR) ≥ 0.7, or VCDR > 0.8 in the absence of a visual field test. Other inclusion criteria were an open angle on gonioscopy and age of onset older than 35 years. Glaucoma patients diagnosed with secondary causes were excluded from this study. Controls were recruited at the same ophthalmology clinics and underwent identical examinations as the POAG cases. Inclusion criteria were: no signs of glaucoma, IOP ≤ 21 mmHg; VCDR< 0.5, and a VCDR inter-eye asymmetry < 0.2, no family history of glaucoma, and age> 55 years. Case and control status was adjudicated by two experienced ophthalmologists.

1162 participants were genotyped using either the Illumina HumanOmniExpressExome Beadchip (964,193 variants; Illumina, Inc., San Diego, CA, USA; n = 999) or the Illumina

HumanOmni2.5Exome Beadchip (2,406,945 variants; Illumina, Inc., San Diego, CA, USA; n = 163). Extensive quality control (QC) was performed on the genotyped data in PLINK v1.07[3]. Variants with a call rate < 95%, as well as variants showing an extreme deviation from Hardy–Weinberg equilibrium ($P < 1 \times 10^{-6}$), or MAF < 0.01 were excluded. All SNPs were mapped to genome build hg19/GRCh37. Individual level QC was performed by exclusion of individuals with a call rate < 95%, discordant sex in self-report versus genetically determined sex, excess or reduced heterozygosity, relatedness (PI-HAT > 0.25) or duplicative samples based on identity by descent (IBD) sharing calculations. The final dataset consisted of 663 and 476 successfully genotyped POAG cases and controls, respectively.

Imputation of unknown genetic variation was performed by means of the "cosmopolitan" approach of using all available populations in a reference panel. The 1000 Genomes Project phase III version 5 was used as an imputation reference panel for GIGA[39]. The pipeline implemented at the Michigan Imputation Server (https://imputationserver.sph.umich.edu) was used for prephasing and imputation (Minimac) of GIGA genotypes[6]. Association testing was performed using EPACTS (https://genome.sph.umich.edu/wiki/EPACTS) in a Firth bias-corrected logistic likelihood ratio test with age, sex, and the top five PCs fitted as covariate.

Eyes of Africa Genetic Consortium (EAGC)

The Eyes of Africa Genetic consortium is a Pan-African study of genetic determinants of POAG, and comprises studies recruited from Ghana, Nigeria, South Africa and the USA, totaling a sample size of 2320 POAG cases and 2121 controls. The methods of ascertaining POAG cases has been described in detail elsewhere[40]. In brief, POAG cases

met the following inclusion criteria: glaucomatous optic neuropathy (VCDR > 0.7 or notch in the neuroretinal rim), and visual field loss (examined by frequency doubling technology or standard automated perimetry) consistent with optic nerve damage in at least one eye. Controls were participants with no known first-degree relative with glaucoma, IOP less than 21 mmHg in both eyes without treatment, and no evidence of glaucomatous optic neuropathy in either eye. Genotyping of cases and controls was performed on the Illumina OmniExpressExome array. Imputation was carried out using IMPUTE2 and 1000G phase1 version 3 as the reference panel. SNPs with imputation quality (r2) > 0.3 and MAF > 0.001 were carried forward for analysis. The association between individual SNP genotypes and primary open-angle glaucoma risk was modeled additively for each copy of the minor allele using logistic regression adjusted for the top 3 principal components of population stratification using PLINK, version 1.9. For imputed genotypes, the information content for allele dosage (range, 0-1; 1 indicates perfect information) were included into the association test model to account for, and average across imputation uncertainty[41]. This study adhered to the tenets of the Declaration of Helsinki. Written informed consent was obtained from all participating individuals. The research was reviewed and approved by the institutional review board from all participating institutions, including Duke University Medical Center (Durham, NC), the Massachusetts Eye and Ear Infirmary (Boston, MA), the University of Michigan (Ann Arbor, MI), New York Eye and Ear Infirmary (New York, NY), the University of Alabama at Birmingham (Birmingham, AL), and, for Ghanaian subjects, Noguchi Memorial Institute of Medical Research of the College of Health Sciences, University of Ghana in Accra.

Supplementary Notes 2: Acknowledgements

The UK Biobank was established by the Wellcome Trust medical charity, Medical Research Council (UK), Department of Health (UK), Scottish Government, and Northwest Regional Development Agency. It also had funding from the Welsh Assembly Government, British Heart Foundation, and Diabetes UK. The eye and vision dataset has been developed with additional funding from The NIHR Biomedical Research Centre at Moorfields Eye Hospital and the UCL Institute of Ophthalmology, Fight for Sight charity (UK), Moorfields Eye Charity (UK), The Macula Society (UK), The International Glaucoma Association (UK) and Alcon Research Institute (USA).

Following biobanks are acknowledged for collecting the FinnGen project samples: Auria Biobank (www.auria.fi/biopankki), THL Biobank (www.thl.fi/biobank), Helsinki Biobank (www.helsinginbiopankki.fi), Biobank Borealis of Northern Finland (https://www.ppshp.fi/Tutkimus-ja-opetus/Biopankki/Pages/Biobank-Borealis-briefly-in-English.aspx), Finnish Clinical Biobank Tampere (www.tays.fi/en-US/Research_and_development/Finnish_Clinical_Biobank_Tampere), Biobank of Eastern Finland (www.ita-suomenbiopankki.fi/en), Central Finland Biobank (www.ksshp.fi/fi-FI/Potilaalle/Biopankki), Finnish Red Cross Blood Service Biobank (www.veripalvelu.fi/verenluovutus/biopankkitoiminta) and Terveystalo Biobank (www.terveystalo.com/fi/Yritystietoa/Terveystalo-Biopankki/Biopankki/). All Finnish Biobanks are members of BBMRI.fi infrastructure (www.bbmri.fi).

We thank the research participants and employees of 23andMe who contributed to this study. We thank Scott Wood, John Pearson and Scott Gordon from QIMR Berghofer for support. We are grateful to all the participants of the EPIC-Norfolk study who have been

part of the project and to the many members of the study teams at the University of Cambridge who have enabled this research.

Data for the AD GWAS study was prepared, archived, and distributed by the National Institute on Aging Alzheimer's Disease Data Storage Site (NIAGADS) at the University of Pennsylvania (U24-AG041689-01), funded by the National Institute on Aging.

References

1. Souzeau, E. et al. Australian and New Zealand Registry of Advanced Glaucoma: methodology and recruitment. Clin. Experiment. Ophthalmol. **40**, 569–575 (2012).

2. Gharahkhani, P. et al. Common variants near ABCA1, AFAP1 and GMDS confer risk of primary open-angle glaucoma. Nat. Genet. **46**, 1120–1125 (2014).

3. Purcell, S. et al. PLINK: a tool set for whole-genome association and population-based linkage analyses. Am. J. Hum. Genet. **81**, 559–575 (2007).

4. Chang, C. C. et al. Second-generation PLINK: rising to the challenge of larger and richer datasets. Gigascience **4**, 7 (2015).

5. Delaneau, O., Marchini, J. & Zagury, J.-F. A linear complexity phasing method for thousands of genomes. Nat. Methods **9**, 179–181 (2011).

6. Das, S. et al. Next-generation genotype imputation service and methods. Nat. Genet. **48**, 1284–1287 (2016).

7. McCarthy, S. et al. A reference panel of 64,976 haplotypes for genotype imputation. Nat. Genet. **48**, 1279–1283 (2016).

8. Marchini, J. & Howie, B. Genotype imputation for genome-wide association studies.

Nat. Rev. Genet. **11**, 499–511 (2010).

9. Wellcome Trust Case Control Consortium. Genome-wide association study of 14,000 cases of seven common diseases and 3,000 shared controls. Nature **447**, 661–678 (2007).

10. Wiggs, J. L. et al. Common variants at 9p21 and 8q22 are associated with increased susceptibility to optic nerve degeneration in glaucoma. PLoS Genet. **8**, e1002654 (2012).

11. Wiggs, J. L. et al. The NEIGHBOR consortium primary open-angle glaucoma genome-wide association study: rationale, study design, and clinical variables. J. Glaucoma **22**, 517–525 (2013).

12. Wiggs, J. L. et al. Common variants near CAV1 and CAV2 are associated with primary open-angle glaucoma in Caucasians from the USA. Hum. Mol. Genet. **20**, 4707–4713 (2011).

13. Pasquale, L. R., Willett, W. C., Rosner, B. A. & Kang, J. H. Anthropometric measures and their relation to incident primary open-angle glaucoma. Ophthalmology **117**, 1521–1529 (2010).

14. Cornelis, M. C. et al. The Gene, Environment Association Studies consortium (GENEVA): maximizing the knowledge obtained from GWAS by collaboration across studies of multiple conditions. Genet. Epidemiol. **34**, 364–372 (2010).

15. Sinnott, J. A. & Kraft, P. Artifact due to differential error when cases and controls are imputed from different platforms. Hum. Genet. **131**, 111–119 (2012).

16. Manichaikul, A. et al. Robust relationship inference in genome-wide association

studies. Bioinformatics **26**, 2867–2873 (2010).

17. Pasquale, L. R. et al. Prospective study of type 2 diabetes mellitus and risk of primary open-angle glaucoma in women. Ophthalmology **113**, 1081–1086 (2006).

18. Riboli, E. & Kaaks, R. The EPIC Project: rationale and study design. European Prospective Investigation into Cancer and Nutrition. Int. J. Epidemiol. **26 Suppl 1**, S6–14 (1997).

19. Day, N. et al. EPIC-Norfolk: study design and characteristics of the cohort. European Prospective Investigation of Cancer. Br. J. Cancer **80 Suppl 1**, 95–103 (1999).

20. Hayat, S. A. et al. Cohort profile: A prospective cohort study of objective physical and cognitive capability and visual health in an ageing population of men and women in Norfolk (EPIC-Norfolk 3). Int. J. Epidemiol. **43**, 1063–1072 (2014).

21. Khawaja, A. P. et al. The EPIC-Norfolk Eye Study: rationale, methods and a cross-sectional analysis of visual impairment in a population-based cohort. BMJ Open **3**, (2013).

22. Chan, M. P. Y. et al. Glaucoma and intraocular pressure in EPIC-Norfolk Eye Study: cross sectional study. BMJ **358**, j3889 (2017).

23. MacGregor, S. et al. Genome-wide association study of intraocular pressure uncovers new pathways to glaucoma. Nat. Genet. (In press).

24. Banda, Y. et al. Characterizing Race/Ethnicity and Genetic Ancestry for 100,000 Subjects in the Genetic Epidemiology Research on Adult Health and Aging (GERA) Cohort. Genetics **200**, 1285–1295 (2015).

25. Kvale, M. N. et al. Genotyping Informatics and Quality Control for 100,000 Subjects in the Genetic Epidemiology Research on Adult Health and Aging (GERA) Cohort. Genetics **200**, 1051–1060 (2015).

26. Choquet, H. et al. A large multi-ethnic genome-wide association study identifies novel genetic loci for intraocular pressure. Nat. Commun. **8**, 2108 (2017).

27. Hoffmann, T. J. et al. Next generation genome-wide association tool: design and coverage of a high-throughput European-optimized SNP array. Genomics **98**, 79–89 (2011).

28. Hoffmann, T. J. et al. Design and coverage of high throughput genotyping arrays optimized for individuals of East Asian, African American, and Latino race/ethnicity using imputation and a novel hybrid SNP selection algorithm. Genomics **98**, 422–430 (2011).

29. Pardo, L. M., MacKay, I., Oostra, B., van Duijn, C. M. & Aulchenko, Y. S. The effect of genetic drift in a young genetically isolated population. Ann. Hum. Genet. **69**, 288–295 (2005).

30. Axenovich, T. I. et al. Linkage analysis of adult height in a large pedigree from a Dutch genetically isolated population. Hum. Genet. **126**, 457–471 (2009).

31. Ikram, M. A. et al. The Rotterdam Study: 2018 update on objectives, design and main results. Eur. J. Epidemiol. **32**, 807–850 (2017).

32. Springelkamp, H. et al. Incidence of glaucomatous visual field loss after two decades of follow-up: the Rotterdam Study. Eur. J. Epidemiol. **32**, 691–699 (2017).

33. Lavanya, R. et al. Methodology of the Singapore Indian Chinese Cohort (SICC) eye

study: quantifying ethnic variations in the epidemiology of eye diseases in Asians. Ophthalmic Epidemiol. **16**, 325–336 (2009).

34. Osman, W., Low, S.-K., Takahashi, A., Kubo, M. & Nakamura, Y. A genome-wide association study in the Japanese population confirms 9p21 and 14q23 as susceptibility loci for primary open angle glaucoma. Hum. Mol. Genet. **21**, 2836–2842 (2012).

35. Shiga, Y. et al. Genome-wide association study identifies seven novel susceptibility loci for primary open-angle glaucoma. Hum. Mol. Genet. **27**, 1486–1496 (2018).

36. Horikoshi, M. et al. Elucidating the genetic architecture of reproductive ageing in the Japanese population. Nat. Commun. **9**, 1977 (2018).

37. Suzuki, K. et al. Identification of 28 new susceptibility loci for type 2 diabetes in the Japanese population. Nat. Genet. **51**, 379–386 (2019).

38. Foster, P. J., Buhrmann, R., Quigley, H. A. & Johnson, G. J. The definition and classification of glaucoma in prevalence surveys. Br. J. Ophthalmol. **86**, 238–242 (2002).

39. 1000 Genomes Project Consortium et al. A global reference for human genetic variation. Nature **526**, 68–74 (2015).

40. Liu, Y. et al. Investigation of known genetic risk factors for primary open angle glaucoma in two populations of African ancestry. Invest. Ophthalmol. Vis. Sci. **54**, 6248–6254 (2013).

41. Genetics of Glaucoma in People of African Descent (GGLAD) Consortium, Hauser MA et al. Association of genetic variants with Primary Open-angle Glaucoma

among Individuals with African Ancestry. JAMA. 2019 Nov 5;322(17):1682-1691.

doi: 10.1001/jama.2019.16161. PMID: 31688885
