# Supplementary material for: Genome-wide meta-analysis identifies 127 open-angle glaucoma loci with consistent effect across ancestries
Source: Nat Commun. 2021 Feb 24;12:1258. doi: 10.1038/s41467-020-20851-4 (PMC7904932; doi:10.1038/s41467-020-20851-4)
Supplement: Supplementary file 3 — Description of Additional Supplementary Files [file 41467_2020_20851_MOESM3_ESM.pdf]

Description of Additional Supplementary Files

File Name: Supplementary Data 1.

Description: Study overview of the meta-analysis. This table summarizes the sample size for the overall POAG, sex-stratified, and subtype-stratified analyses, as well as age distribution for each contributing site.

File Name: Supplementary Data 2.

Description: Independent genome-wide significant loci in Europeans. This table provides summary statistics for the most significant genome-wide associated SNPs from the GWAS meta-analysis of POAG in Europeans. Independent loci were identified through the Conditional and Joint (COJO) analysis in GCTA v1.26. The corresponding GWAS summary statistics in UKBB (glaucoma self-report), Asian, and African studies have also been provided. Two loci (*PKHD1* and *TFEC-TES*) had suggestive association in the meta-analysis (P<5e-06) but became significant after performing the COJO analysis. CHR, chromosome; EUR, European; P_COJO, P-value from the Conditional and Joint analysis; unknown: risk loci that were not genome-wide significant (P<5e-08) for POAG in previous studies (some of these loci were genome-wide significant for IOP or VCDR previously, but did not reach genome-wide significance for POAG risk).

File Name: Supplementary Data 3.

Description: Genome-wide significant Loci in Asians and Africans. ASN, Asian; EUR, European; AFR, African.

File Name: Supplementary Data 4.

Description: Independent genome-wide significant loci in the GWAS meta-analysis of POAG across ancestries. This table provides summary statistics for the most significant SNPs from the GWAS meta-analysis of POAG in Europeans, Asians, and Africans. The corresponding GWAS summary statistics for each subgroup (Europeans, UKBB self-reports, Asians, and Africans) as well as 23andMe (the replication data; GWAS of 43,254 glaucoma cases vs 1,471,118 controls) have also been provided. CHR, chromosome; meta, meta-analysis; EUR, European; unknown: risk loci that were not genome-wide significant (P<5e-08) for POAG in previous studies (some of these loci were genome-wide significant for IOP or VCDR previously, but did not reach genome-wide significance for POAG risk).

File Name: Supplementary Data 5.

Description: The association of the POAG risk SNPs with IOP and VCDR. The results are from GWAS meta-analysis of N=133,492 IOP and N=90,939 VCDR. CHR, chromosome; meta, meta-analysis. *rs6755023 in LD r2=0.98 with rs200621439. **rs327736 in LD r2=0.90 with rs59101260. ***rs35220810 in LD r2=1.0 with rs35740987. ****rs62063276 in LD r2=0.95 with rs114919114.

File Name: Supplementary Data 6.

Description: The association of the POAG risk SNPs with macular thickness. Freq, frequency of the effect allele; info, imputation quality score; GCIPL, ganglion cell-inner plexiform layer; RNFL, retinal nerve fiber layer.

File Name: Supplementary Data 7.

Description: Top colocalization results between all POAG loci and AD using the Kunkle *et al.* GWAS meta-analysis. This table presents the top colocalization results using eCAVIAR for each of the 123 and 66 autosomal POAG variants found with the cross-ancestry and European POAG GWAS meta-analysis, respectively, relative to the Kunkle *et al.*, 2019 AD GWAS meta-analysis. For each POAG variant, we listed all significant variants per linkage disequilibrium (LD)-based locus (defined in Methods) at CLPP>0.01, and if no variants passed this cutoff, the variant with the highest CLPP value was listed. The effect alleles of the POAG and AD GWAS were aligned to allele 1 (A1) in 1000 Genomes Project (1KG), which was used as the reference panel for computing local pairwise linkage disequilibrium (LD) inputted into eCAVIAR. If the lead POAG variant was not found in 1KG, an LD proxy variant at $r^2>0.8$ was found in GTEx release v7 (https://www.gtexportal.org/home/datasets). The beta and standard eroro CLPP, Colocalizaiton Posterior Probability; A1, Effect allele in 1KG used for eCAVIAR analysis; A2, Other allele in 1KG; Effect, beta; StdErr, standard error of beta.

File Name: Supplementary Data 8.

Description: Top colocalization results between all POAG loci and AD using the Jansen *et al.* GWAS meta-analysis. Similar to Supplementary Data 7, just using summary statistics from the Jansen et al., 2019 AD GWAS meta-analysis for the colocalization analysis with POAG summary statistics.

File Name: Supplementary Data 9.

Description: Genetic correlation between POAG and the other traits. These results were obtained based on the LD score regression approach through the LD Hub platform. rg, genetic correlation estimate; se, standard error of the genetic correlation

estimate; h2_obs, heritability estimate on the observed scale; h2_obs_se, standard error of the heritability estimate on the observed scale; h2_int, intercept from the heritability analysis; h2_int_se, standard error of the intercept from the heritability analysis; gcov_int, intercept from the genetic correlation analysis; gcov_int_se, standard error of the intercept from the genetic correlation analysis.

File Name: Supplementary Data 10.

Description: Cross-ancestry fine mapping of the POAG risk loci. This table presents the most plausible causal visitants identified using the fine-mapping approach implemented in the software PAINTOR. The posterior probability of a SNP being causal has been provided for the European, Asian, and African meta-analyses, separately, as well as after integrating GWAS data across ancestries. Color codes have been provided at the bottom of the spreadsheet.

File Name: Supplementary Data 11.

Description: Previously unreported significant MAGMA gene-based results. The genome-wide significant (P<2.5e-06) gene-based results obtained from MAGMA, which are independent of the risk loci identified in the single variant-based tests (i.e., located at least >1Mb apart from the risk loci identified in the meta-analysis), and were not previously reported for POAG. The corresponding results for each subgroup (Europeans, UKBB self-reports, Asians, and Africans) have also been provided. *COL4A3* and *FLNB* were previously reported for IOP and VCDR, respectively, but did not reach genome-wide significance for POAG risk. CHR, chromosome.

File Name: Supplementary Data 12.

Description: Significant MAGMA gene-set results. The genome-wide significant (P<4.7e-06) pathways obtained from MAGMA. The corresponding results for each subgroup (Europeans, UKBB self-reports, Asians, and Africans) have also been provided.

File Name: Supplementary Data 13.

Description: The most significant POAG genes within the significant pathways. Genes within the significant pathways identified by MAGMA that are at least suggestively associated (P<5e-05) with POAG in the MAGMA gene-based test.

File Name: Supplementary Data 14.

Description: Annotation and functional relevance of the POAG risk loci identified in this study. This is an expanded version of Figure 5 which provides additional information such as the name of genes that were prioritized in each locus in gene-based tests, name of genes that are within significant pathways, SNP IDs that were prioritized by fine mapping in PAINTOR, and whether the prioritized SNPs are eQTL/sQTL in GTEX.

File Name: Supplementary Data 15.

Description: eQTL results for the POAG risk SNPs. This table shows eQTL results for the most significant POAG risk SNPs or those in high LD (r2>0.8) with the most significant SNPs. These results were obtained from GTEX eQTL v6 (all tissues), Blood eQTL browser, BIOS QTL Browser, and BRAINEAC.

File Name: Supplementary Data 16.

Description: CADD and RegulomeDB pathogenicity scores for the POAG risk SNPs.

File Name: Supplementary Data 17.

Description: The significant POAG genes identified by MetaXcan. eQTL data from retina (EyeGEx study, N = 406) and 44 GTEX tissues were used for this analysis. Var_g, variance of genes expression; pred_perf_r2, R2 of the predicted gene expression to gene's measured transcriptome (prediction performance); pred_perf_pval, P-value of the prediction performance; pred_perf_qval, q-value of the prediction performance; n_snps_used, number of SNPs used from GWAS; n_snps_in_cov, number of SNPs in the covariance matrix; n_snps_in_model; number of SNPs used in the model.

File Name: Supplementary Data 18.

Description: The significant POAG genes identified by SMR. The POAG meta-analysis results in Europeans were integrated with eQTL data from blood (CAGE study, N=2,765) and retina (EyeGEx study, N = 406) using the SMR approach. A1, Effect allele; A2, Other allele; Freq, frequency of the effect allele.

File Name: Supplementary Data 19.

Description: Gene prioritization by FOCUS. Tx_start, transcription start site; tx_stop, transcription end site; cv.R2, cross-validation predictive R Squared; cv.R2.pval, P-value of cross-validation; twas_z, marginal Z score from TWAS; pip, marginal posterior inclusion probability; in_cred_set, whether or not model is included in the credible set.

File Name: Supplementary Data 20.

Description: Expression of the previously unreported POAG risk genes in eye tissues. Presented genes are the nearest genes to the GWAS lead SNPs identified in the meta-analysis.

File Name: Supplementary Data 21.

Description: Candidate drugs for the POAG risk genes. pLI, this is the pLI score from the ExAC database showing the probability of being loss-of-function intolerant. The higher the score, the more intolerant the variant; ncRVIS, this is the non-coding residual variation intolerance score. The higher the score, the more intolerant the variant; posMapSNPs, this shows the number of SNPs which were mapped to genes based on FUMA positional mapping; posMapMaxCADD, the maximum CADD score of SNPs which were mapped to genes based on FUMA positional mapping; eqtlMapSNPs, the number of SNPs which were mapped to genes based on FUMA eQTL mapping; eqtlMapminP, the minimum eQTL P-value of the mapped SNPs by FUMA eQTL mapping; eqtlMapminQ, the minimum eQTL FDR of the mapped genes by FUMA eQTL mapping; ciMap, this indicates whether or not the gene is mapped by FUMA chromatin interaction mapping; ciMapts, this shows which tissues or cell types if there is a “yes” to ciMap column.

File Name: Supplementary Data 22.

Description: The top genome-wide significant SNPs for the male-stratified analysis. The corresponding statistics for the female-stratified analysis as well as the overall POAG meta-analysis has also been shown. The SNP presented in bold was not genome-wide significant in the meta-analysis. CHR, chromosome; meta, meta-analysis.

File Name: Supplementary Data 23.

Description: The top genome-wide significant SNPs for the female-stratified analysis. The corresponding statistics for the male-stratified analysis as well as the overall POAG meta-analysis has also been shown. The SNPs presented in bold were not genome-wide significant in the meta-analysis. CHR, chromosome; meta, meta-analysis.

File Name: Supplementary Data 24.

Description: The top genome-wide significant SNPs for the NTG-stratified analysis. The corresponding statistics for the HTG-stratified analysis as well as the overall POAG meta-analysis has also been shown. The SNP presented in bold was not genome-wide significant in the meta-analysis. CHR, chromosome; meta, meta-analysis; P_diff, P-value for the difference between the estimated effect sizes for NTG vs HTG.

File Name: Supplementary Data 25.

Description: The top genome-wide significant SNPs for the HTG-stratified analysis. The corresponding statistics for the NTG-stratified analysis as well as the overall POAG meta-analysis has also been shown. CHR, chromosome; meta, meta-analysis; P_diff, P-value for the difference between the estimated effect sizes for NTG vs HTG.
