# Supplementary material for: Genome-wide meta-analysis identifies 127 open-angle glaucoma loci with consistent effect across ancestries
Source: Nat Commun. 2021 Feb 24;12:1258. doi: 10.1038/s41467-020-20851-4 (PMC7904932; doi:10.1038/s41467-020-20851-4)
Supplement: Supplementary file 5 — Reporting Summary [file 41467_2020_20851_MOESM5_ESM.pdf]

nature research

Corresponding author(s): Puya Gharahkhani

Last updated by author(s): Oct 29, 2020

Reporting Summary

Nature Research wishes to improve the reproducibility of the work that we publish. This form provides structure for consistency and transparency in reporting. For further information on Nature Research policies, see our Editorial Policies and the Editorial Policy Checklist.

Statistics

For all statistical analyses, confirm that the following items are present in the figure legend, table legend, main text, or Methods section.

n/a	Confirmed	
☐	☒	The exact sample size (*n*) for each experimental group/condition, given as a discrete number and unit of measurement
☐	☒	A statement on whether measurements were taken from distinct samples or whether the same sample was measured repeatedly
☐	☒	The statistical test(s) used AND whether they are one- or two-sided<br>*Only common tests should be described solely by name; describe more complex techniques in the Methods section.*
☐	☒	A description of all covariates tested
☐	☒	A description of any assumptions or corrections, such as tests of normality and adjustment for multiple comparisons
☐	☒	A full description of the statistical parameters including central tendency (e.g. means) or other basic estimates (e.g. regression coefficient) AND variation (e.g. standard deviation) or associated estimates of uncertainty (e.g. confidence intervals)
☐	☒	For null hypothesis testing, the test statistic (e.g. *F*, *t*, *r*) with confidence intervals, effect sizes, degrees of freedom and *P* value noted<br>*Give P values as exact values whenever suitable.*
☒	☐	For Bayesian analysis, information on the choice of priors and Markov chain Monte Carlo settings
☒	☐	For hierarchical and complex designs, identification of the appropriate level for tests and full reporting of outcomes
☐	☒	Estimates of effect sizes (e.g. Cohen's *d*, Pearson's *r*), indicating how they were calculated

Our web collection on statistics for biologists contains articles on many of the points above.

Software and code

Policy information about availability of computer code

Data collection	No software was used for data collection.
Data analysis	Imputation: Minimac3 through the Michigan Imputation Server<br>Association testing: PLINK2, SNPTEST v2.5.1, SAIGE v0.36.3, EPACTS v3.2.6, RVtests v2.0.3, METAL (the version released on 2011-03-25), GCTA v1.26, R 3.2.2, and LocusZoom v1.4<br>Genetic correlation: ldsc 1.0.0 and Popcorn v0.9.9<br>Genetic co-localization: eCAVIAR v2.0.0<br>Fine-mapping: PAINTOR v3.0<br>Gene expression: Trimgalore (v0.4.0) and edgeR v3.22.5<br>Post-GWAS (gene-based tests): MAGMA v1.07b, MetaXcan v0.3.3, SMR v0.69, and FOCUS v0.5<br>Post-GWAS (eQTL, chromatic interaction, and bioinformatic functional analyses): RegulomeDB v1.1, CADD v1.4, and ANNOVAR (the version released on 2016Feb01) through the FUMA platform v1.3.5 (https://fuma.ctglab.nl/)

For manuscripts utilizing custom algorithms or software that are central to the research but not yet described in published literature, software must be made available to editors and reviewers. We strongly encourage code deposition in a community repository (e.g. GitHub). See the Nature Research guidelines for submitting code & software for further information.

Data

Policy information about availability of data

All manuscripts must include a data availability statement. This statement should provide the following information, where applicable:

- Accession codes, unique identifiers, or web links for publicly available datasets
- A list of figures that have associated raw data
- A description of any restrictions on data availability

The GWAS summary statistics generated in this study will be available upon publication of this paper via the GWAS Catalog website https://www.ebi.ac.uk/gwas/downloads/summary-statistics under study accession identifiers GCST90011766, GCST90011767, GCST90011768, GCST90011769, and GCST90011770.
UK Biobank data including POAG, VCDR, IOP, RNFL, and GCIPL GWASs are available by request through the UK Biobank Access Management System https://www.ukbiobank.ac.uk/. The GWAS result from 23andMe are available by request from https://www.23andme.com/. Restrictions apply to the availability of these data, which were used under license for the current study, and so are not publicly available.
The GWAS results for Alzheimer's disease that we used for this study are available from https://ctg.cncr.nl/software/summary_statistics, and by request from https://www.niagads.org/datasets/ng00075.
The Haplotype Reference Consortium (HRC) r1.1 is accessible by request from http://www.haplotype-reference-consortium.org/. We used HRC r1.1 for imputation through the Michigan Imputation server (https://imputationserver.sph.umich.edu/index.html#!). The 1000G Genomes phase 3 data is available at https://www.internationalgenome.org/.
The datasets we used for the functional analyses in this study are available through: GTEX eQTL v6 (https://gtexportal.org/home/), Blood eQTL (https://genenetwork.nl/bloodeqtlbrowser/), BIOS QTL (https://genenetwork.nl/biosqtlbrowser/), EyeGEx data (https://www.ncbi.nlm.nih.gov/geo/query/acc.cgi?acc=GSE115828), BRAINEAC (http://www.braineac.org/), Hi-C data from 21 tissue/cell types from (https://www.ncbi.nlm.nih.gov/; GEO accession GSE87112), PsychENCODE (https://www.nimhgenetics.org/resources/psychencode), Giusti-Rodriguez et al 2019 (https://www.biorxiv.org/content/10.1101/406330v2), and FANTOM5 Human Enhancer Tracks (http://slidebase.binf.ku.dk/human_enhancers/presets). These datasets were used through the FUMA platform (https://fuma.ctglab.nl/)
The drug target data was obtained through the Open Targets platform (https://genetics.opentargets.org/)

Field-specific reporting

Please select the one below that is the best fit for your research. If you are not sure, read the appropriate sections before making your selection.

- [x] Life sciences
- [ ] Behavioural & social sciences
- [ ] Ecological, evolutionary & environmental sciences

For a reference copy of the document with all sections, see nature.com/documents/nr-reporting-summary-flat.pdf

Life sciences study design

All studies must disclose on these points even when the disclosure is negative.

Sample size	Rather than performing a power calculation, we collected the largest possible GWAS for POAG to date to identify novel risk loci.We collected 34,179 POAG cases and 349,321 controls including participants of European, Asian, and African descent from 21 independent studies across the world.
Data exclusions	Pre-imputation QC: Study-specific QC and imputation details have been provided in Supplementary Information. Overall, SNPs with >5% missing genotypes, minor allele frequency (MAF) < 0.01, and evidence of significant deviations from Hardy-Weinberg equilibrium (HWE) were excluded. In addition, individuals with >5% missing genotypes, one of each pair of related individuals (detected based on a p-hat>0.2 from identity by descent calculated from autosomal markers), and ancestry outliers from each study (detected based on principal component analysis including study participants and reference samples of known ancestry) were excluded from further analysis (for more details please see Supplementary Information).<br>Post-imputation QC: SNPs with MAF > 0.001 and imputation quality scores (INFO or r2)> 0.3 were taken forward for association analysis.
Replication	We validated the association of the genome-wide significant SNPs from our cross-ancestry meta-analysis in a dataset comprising 43,254 participants with self-reported POAG and 1,471,118 controls from 23andMe, Inc. Of the 127 loci, the association results for 125 SNPs were available in 23andMe, 120 of which (96%) were replicated at P<0.05, and 106 (85%) after Bonferroni correction for 125 independent tests (Supplementary Data 4). The correlation of the effect size was r=0.98 (95% CIs 0.977-0.989).
Randomization	Samples were derived from existing studies and were not randomized. This is a case-control study where cases were those diagnosed with glaucoma, and controls were unscreened population controls. Association testing adjusted for age, sex, and principal components as covariates.
Blinding	GWAS QCs including the removal of ancestry outliers and individuals with high genotype missing rates, as well as SNPs with low minor allele frequencies, were performed blind to the case-control status of the participants. However, filtering SNPs based on the Hardy-Weinberg Equilibrium (HWE) was not blind to the case-control status. This is because some of the disease risk variants may not follow the HWE in diseased populations due to the reasons such as selection pressure. Hence, HWE filtering was applied to the control sets only. Another QC

step which was not blind to the phenotypes was the removal of SNPs with differential genotype missing rates between cases and controls to avoid artefact findings.

Reporting for specific materials, systems and methods

We require information from authors about some types of materials, experimental systems and methods used in many studies. Here, indicate whether each material, system or method listed is relevant to your study. If you are not sure if a list item applies to your research, read the appropriate section before selecting a response.

Materials & experimental systems

n/a	Involved in the study	
x		Antibodies
x		Eukaryotic cell lines
x		Palaeontology and archaeology
x		Animals and other organisms
	x	Human research participants
x		Clinical data
x		Dual use research of concern

Methods

n/a	Involved in the study	
x		ChIP-seq
x		Flow cytometry
x		MRI-based neuroimaging

Human research participants

Policy information about studies involving human research participants

Population characteristics

The population characteristics for each contributing study has been provided in the Supplementary Information and Supplementary Data 1. We also investigated the expression of the genes nearest to the lead SNP for the novel loci in 21 healthy eye tissues. The average age for these individuals was 71 (SD=15) years, majority (%92) were male, all were Caucasian, and they did not have any glaucoma diagnosis. Causes of death for these individuals were cancer, brain injuries, and heart attack.

Recruitment

This study recruited 34,179 primary open-angle glaucoma (POAG) cases and 349,321 controls including participants of European, Asian, and African descent from 21 independent studies across the world. Number of cases and controls, and distribution of age and sex for each study are summarized in Supplementary Data 1. The phenotype definition and additional details such as genotyping platforms for each study are provided in Supplementary Information. For most of the studies, we restricted glaucoma to POAG based on the ICD9/ICD10 criteria. However, considering that POAG constitutes the majority of glaucoma cases in Europeans, we also included 7,286 glaucoma self-reports from UK Biobank (UKBB) to replicate findings from the ICD9/ICD10 POAG meta-analysis in Europeans and to maximize the statistical power of the final stage meta-analysis.

Recruitment biases:
Self-reports: the self-reported glaucomas in UKBB is unlikely to bias the results of this study as we observed a very high concordance between the GWAS results for clinically validated cases versus self-reports in UKBB. Additionally, the vast majority of our results replicated in self-report data from 23andMe.

Age of participants: although where possible each participating study adjusted for the effect of age in their association testing prior to the meta-analysis, in a subset of studies, cases and controls were not matched for age and future studies should fully investigate the effect of the identified risk loci across different age strata, particularly for loci where certain alleles are strongly associated with other age-related conditions.

Ethics oversight

There was no ethics oversight in this study. Informed consent was obtained from all the participants, and ethics approval was obtained from the ethics committee of all the participating institutions as follows:
ANZRAG - the Human Research Ethics Committees of Southern Adelaide Health Service/Flinders University, University of Tasmania, QIMR Berghofer Medical Research Institute, and the Royal Victorian Eye and Ear Hospital.
NEIGHBOR/MEEI - the Massachusetts Eye and Ear Infirmary (MEEI) institutional review board.
NHS/HPFS - the Partners institutional review board and the Harvard School of Public Health Institutional review board.
EPIC-Norfolk Eye Study - the Norfolk Local Research Ethics Committee (05/Q0101/191) and East Norfolk & Waveney NHS Research Governance Committee (2005EC07L).
UKBB - the National Research Ethics Service Committee North West – Haydock.
Kaiser Permanente GERA Cohort - the Institutional Review Board of the Kaiser Foundation Research Institute.
King’s College London (KCL) - the St. Thomas' Hospital Local Research Ethics Committee.
Blue Mountain Eye Study (BMES) - the Western Sydney Area Health Service Human Ethics Committee.
Southampton - the Southampton and South West Hampshire Local Research Ethics Committee (05/Q1702/8).
Gutenberg Health Study (GHS) - the Medical Ethics Committee of the University Medical Center Mainz and the local and federal data safety commissioners.
Erasmus Rucphen Family (ERF) study - the medical ethics committee of the Erasmus Medical Center, Rotterdam.
Rotterdam Study I - the institutional review board (Medical Ethics Committee) of the Erasmus Medical Center and the review board of The Netherlands Ministry of Health, Welfare and Sports.
Geisinger - the Geisinger Institutional Review Board.
FinnGen - the Coordinating Ethics Committee of the Hospital District of Helsinki and Uusimaa (HUS; Nr HUS/990/2017).
Singapore Chinese Eye Study (SCES) - The local institutional ethics review committee (CIRB).

The Chinese University of Hong Kong (CUHK) - the Ethics Committee for Human Research, the Chinese University of Hong Kong.
Japanese Tohoku - Institutional Review Board of the Tohoku Graduate School of Medicine.
Biobank Japan - the ethical committees at the Institute of Medical Science, the University of Tokyo, and the Center for Genomic Medicine, RIKEN, Yokohama Institute, Japan.
GIGA - The study was conducted according to the guidelines for human research by the National Institute for Medical Research in Tanzania. Ethical approval was obtained from the institutional review boards at each study site.
Eyes of Africa Genetic Consortium (EAGC) - the institutional review board from all participating institutions, including Duke University Medical Center (Durham, NC), the Massachusetts Eye and Ear Infirmary (Boston, MA), the University of Michigan (Ann Arbor, MI), New York Eye and Ear Infirmary (New York, NY), the University of Alabama at Birmingham (Birmingham, AL), and, for Ghanaian subjects, Noguchi Memorial Institute of Medical Research of the College of Health Sciences, University of Ghana in Accra.

Note that full information on the approval of the study protocol must also be provided in the manuscript.
